# Can fruit and vegetable aggregation systems better balance improved producer livelihoods with more equitable distribution?

**DOI:** 10.1016/j.worlddev.2021.105678

**Published:** 2021-12

**Authors:** G.S. Cooper, B. Shankar, K.M. Rich, N.N. Ratna, M.J. Alam, N. Singh, S. Kadiyala

**Affiliations:** a Institute for Sustainable Food and Department of Geography, University of Sheffield, Sheffield, United Kingdom; b Ferguson College of Agriculture, Oklahoma State University, Stillwater, OK, USA; c International Livestock Research Institute (ILRI), West Africa Regional Office, Dakar, Senegal; d Department of Global Value Chain & Trade, Faculty of Agribusiness and Commerce, Lincoln University, Christchurch, New Zealand; e Department of Agribusiness and Marketing, Bangladesh Agricultural University (BAU), Mymensingh, Bangladesh; f Digital Green, North India Office, New Delhi, India; g Department for Population Health, London School of Hygiene and Tropical Medicine (LSHTM), London, United Kingdom

**Keywords:** Horticulture, Markets, Nutrition-sensitive, Trade-offs, South Asia, India

## Abstract

The need for food systems to generate sustainable and equitable benefits for all is a global imperative. However, whilst ample evidence exists linking smallholder farmer coordination and aggregation (i.e. the collective transport and marketing of produce on behalf of multiple farmers) to improved market participation and farmer incomes, the extent to which interventions that aim to improve farmer market engagement may co-develop equitable consumer benefits remains uncertain. This challenge is pertinent to the horticultural systems of South Asia, where the increasing purchasing power of urban consumers, lengthening urban catchments, underdeveloped rural infrastructures and inadequate local demands combine to undermine the delivery of fresh fruits and vegetables to smaller, often rural or semi-rural markets serving nutritionally insecure populations. To this end, we investigate the potential for aggregation to be developed to increase fruit and vegetable delivery to these neglected smaller markets, whilst simultaneously improving farmer returns. Using an innovative system dynamics modelling approach based on an aggregation scheme in Bihar, India, we identify potential trade-offs between outcomes relating to farmers and consumers in smaller local markets. We find that changes to aggregation alone (i.e. scaling-up participation; subsidising small market transportation; mandating quotas for smaller markets) are unable to achieve significant improvements in smaller market delivery without risking reduced farmer participation in aggregation. Contrastingly, combining aggregation with the introduction of market-based cold storage and measures that boost demand improves fruit and vegetable availability significantly in smaller markets, whilst avoiding farmer-facing trade-offs. Critically, our study emphasises the benefits that may be attained from combining multiple nutrition-sensitive market interventions, and stresses the need for policies that narrow the fruit and vegetable cold storage deficits that exist away from more lucrative markets in developing countries. The future pathways and policy options discovered work towards making win–win futures for farmers and disadvantaged consumers a reality.

## Highlights

• Aggregation boosts farmer revenues but risks diverting fruit and vegetable supply away from consumers in small rural markets.
• Via a systems model set in Bihar, India, we identify levers to make horticultural aggregation more relevant to small markets.
• Market-based cold storage and boosting small market demand offer policy levers to improve spatial equity in consumption.
• Intervention combinations improve small market fruit and vegetable availability, whilst avoiding farmer revenue trade-offs.
• Packages of synergistic policies should be embraced to foster ‘win-wins’ in nutrition-sensitive food systems.

## 1 Introduction

Insufficient fruit and vegetable (F&V) consumption is estimated to be responsible for 1.7-million deaths worldwide each year (Lim et al., 2012). Moreover, despite major developments in global agricultural productivity and technology over the past 50 years, only around 18% of individuals in low- and middle-income countries consume the World Health Organisation’s (WHO) recommendation of 400 g/day of F&V (Frank et al., 2019). With the United Nations’ second Sustainable Development Goal aiming to end all forms of malnutrition by 2030 (UN, 2015), achieving and sustaining the adequate delivery of F&V to populations vulnerable to food and nutrition insecurities remains one of the most pressing development challenges of today (Development Initiatives, 2020).

Improving supply is often seen as the first step towards enhancing F&V consumption (Schreinemachers, Simmons, & Wopereis, 2018). However, F&V production in low- and middle-income settings is often beset by problems, particularly in countries such as India where a combination of high risk, underdeveloped markets, high input and knowledge requirements, and a policy environment geared towards staples has stifled the growth of F&V production and associated farmer livelihood outcomes over time (Pingali & Sunder, 2017). Consequently, coordinated activities amongst smallholders has emerged as a broad approach to overcome issues associated with fragmented supplies, asymmetrical power relations and imperfect market information (Fischer and Qaim, 2012, Patil et al., 2016, Trebbin and Hassler, 2012).

*Aggregation* describes activities that organise and pool individual quantities of agricultural products to enable economies of scale in transportation, storage and marketing (Pingali, Aiyar, Abraham, & Rahman, 2019). These activities are often formally facilitated, benefitting from the technical and logistical expertise of non-governmental organisations or large agribusinesses (Cadilhon et al., 2005, Pingali et al., 2019). Previous studies have established associations between participation in aggregation (and related collective activities) and enhanced rates of credit access in Cambodia (Ofori, Sampson, & Vipham, 2019), improved access to vegetable storage in Honduras (Hellin, Lundy, & Meijer, 2007), and improved access to price information in Kenyan fruit markets (Fischer & Qaim, 2012). Evidence from Vietnam, Madagascar and India suggests that the sharing of transport and quality assurance costs typically opens pathways to supply larger, often more exclusive buyers, including domestic supermarkets and high-value exporters (Cadilhon et al., 2005, Minten et al., 2009, Narrod et al., 2009). Therefore, whilst aggregation interventions promote supply responses, their frequent gearing towards wealthier segments of the overall market raises questions about their sensitivity and equity from the perspective of relatively impoverished consumers.

Food systems across Africa and South Asia have also evolved dramatically over the past 30 years (Reardon & Timmer, 2014). Improvements in transport and storage infrastructure have lengthened urban-centric distribution networks (Reardon, 2015), and the comparative economic wealth of urban populations provides a consumer-base that is able to spend more on food (Minten, Reardon, & Chen, 2017). Reinforcing these urban pull factors are the inferior economic distance (i.e. transport costs and infrastructure), capacities and risks of spoilage associated with often more isolated, smaller markets (Reardon, 2015). Economically remote communities can be poorly integrated with other markets, resulting in inferior diversity and relative costs of perishable, nutritious food available, with regional inequalities exaggerated in low and middle income countries (Filmer et al., 2021, Miller et al., 2016). A recent literature demonstrates that remoteness and lack of market access is detrimental to food security and dietary quality (Hirvonen et al., 2017, Stifel and Minten, 2017). Moreover, evidence from Kenya and Ethiopia shows that whilst improved rural connectivity can kickstart positive feedbacks between farmer mobility and market access, such upgrades tend to strengthen the economic viability of farmers to supply urban centres over markets in communities with the greatest barriers to accessible and affordable F&V (Ogutu and Qaim, 2019, Rammelt and Leung, 2017).

Given the need to make food systems work for all (UN, 2015), the extent to which aggregation can advantage farmers whilst simultaneously improving the availability of F&V in smaller, neglected markets remains an open question. Informed by multiple rich datasets and participatory exercises, we develop an innovative system dynamics model of an aggregation scheme in Bihar, India, to explore the trade-offs emerging from adapting aggregation and the wider enabling environment to become more sensitive to F&V availability in smaller, rural or semi-rural markets serving nutritionally insecure populations. Aware that simultaneously achieving positive outcomes for multiple actors is likely to require bundles of interventions (Beauchamp et al., 2018, Glover and Poole, 2019), we then investigate the combinations of upgrades that increase local F&V availability and affordability whilst securing farmer returns and supply responses.

We also address two further research gaps. In June 2020, the Government of India amended the Essential Commodities Act (1955) in an attempt to improve the range and size of buyers available to farmers and encourage investment into the cold storage sector (Government of India, 2020). The scenarios explored below investigate how trade-offs may emerge from policy relevant changes to aggregation and the wider enabling environment, including the upscaling of aggregation to strengthen links between smallholders and larger traders, and investment in cold storage to improve F&V capture in local markets.

Typically the remit of large observational datasets and complex statistical models, this study also contributes to the need to explore the dynamics and trade-offs of multiple interacting food system interventions (Kawabata et al., 2020, Mylona et al., 2018). Therefore, moving away from the tendency to view policy instruments as alternatives (Gunningham & Sinclair, 1999), we explore how multiple complementary market interventions and policy instruments may generate positive outcomes for farmers and consumers in developing food systems.

## 2 Methods

### 2.1 Study context

Bihar is home to an estimated 115 million people, with agriculture employing 80% of the working population (Government of Bihar, 2019). Despite being the fourth largest producer of vegetables and eighth largest producer of fruits amongst Indian states (Government of India, 2018), farmers in Bihar remain amongst the poorest in India (Satyasai, 2016). At the same time, the average per capita F&V consumption rate in Bihar is estimated to be 64–79% of recently estimated Indian national average rates (Choudhury et al., 2021, Choudhury et al., 2020), and 35–45% of the World Health Organisation’s (WHO) recommended 400 g/capita/day (NSSO, 2013). In turn, rural F&V consumption is estimated to be roughly 12% less than urban (NSSO, 2013).

The F&V food system presents numerous barriers to the goal of equitably developing farmer livelihoods at the same time as increasing the availability of F&V for nutritionally insecure populations. Small (1–2 ha) and marginal (<1 ha) landholdings providing 70% of the state’s vegetable production (Sinha & Kumar, 2015). Only half of village roads are paved and Bihar registers the lowest rate of vehicle ownership in India (Government of Bihar, 2019). In turn, owing to the combination of relative transport costs and heightened risks of spoilage, geographically local markets can often appear economically distant; as a consequence, farmers and traders tend to favour higher demand urban markets connected to arterial transport networks. Bihar also has the third widest F&V cold storage deficit of any Indian state, when measured as the difference between the total demand for cold storage facilities for perishable items and existing cold storage capacities (Vanitha, Chaurasia, Singh, & Naik, 2013), whilst spoilage that occurs during summer (March – May) and monsoon (June – September) months underpins wastage rates between 19 and 32% of annual F&V production (Kumari, Bairwa, Meena, & Rahman, 2017).

Against this backdrop, the non-governmental organisation (NGO) Digital Green has been active in the provision of agricultural extension services in Bihar, Odisha and Andhra Pradesh since 2006. Initially specialising in the creation of participatory videos for community-level nutrition education (Gandhi et al., 2007, Kadiyala et al., 2018), Digital Green has since pioneered a number of farm- and market-level interventions based around collective action and the generation of near real-time data dashboards (Gandhi, Aggarwal, & Sasindaran, 2016). Funded by The Bill & Melinda Gates Foundation (BMGF) and USAID, Digital Green launched the ‘Loop’ aggregation scheme in 2016, with the smallholder farmer-focused aims of cutting transportation costs, saving marketing time, and increasing market access (Digital Green, 2017). In essence, a local vehicle owner oversees aggregation for each cluster of two to three villages, with Digital Green providing aggregators with smartphones to record details of market transactions (e.g. crop type, quantities, revenues from sales). This information is then made available to farmers, who receive a digital receipt in the form of a text message. In turn, day-to-day farmer participation is based on verbal agreement only (i.e. non-contractual), with farmers generally contacting their aggregator one day in advance if they intend to supply Loop as opposed to supplying the market themselves. The ‘loop’ completes once farmer revenues are returned, minus the costs of transportation (normally 0.6–0.9 Rs/kg paid to aggregators) and a service charge collected by Digital Green (often 10% of the aggregation charge).

**Fig. 1:**
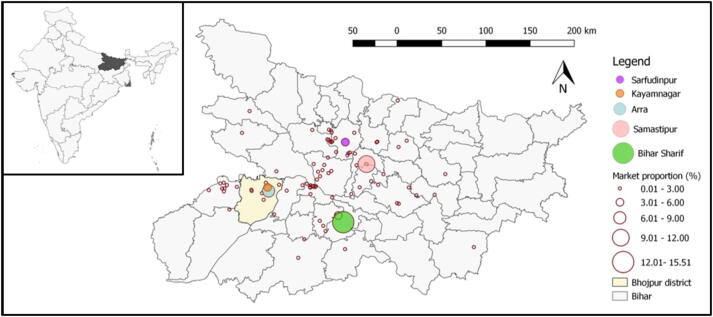
The distribution of aggregated Loop market supplies to 96 markets (red dots) across the northern Indian state of Bihar (inset – grey). The markets that received the top-five highest cumulative volumes of Loop supplies between January 2016 and October 2018 are highlighted in colour. Data: Loop dashboard (see Section 2.2). (For interpretation of the references to colour in this figure legend, the reader is referred to the web version of this article.)

Between January 2016 and September 2018, Loop aggregated over 95,000 tonnes of F&V from 28,000 farmers across Bihar (equivalent to 0.2% of all F&V production in the state over the time period). Participating farmers have reported transport costs being cut in half and savings of between 4 and 8 h each week from the aggregator visiting the market on their behalf (Digital Green, 2017). However, aggregations are predominantly supplied towards urban wholesale markets (Fig. 1), due to the desire of farmers to access higher demand markets and the physical need for sufficient market capacities to absorb aggregations of around 2000 kg. For example, according to Loop dashboard data (Section 2.2), 62% of aggregated volumes in Samastipur district were supplied to two large urban wholesale markets (Tajpur and Samastipur), with the remaining 38% shared between 32 other markets. Similar clustering is present across Bihar, with 85% and 71% of supplies in Nalanda and Bhojpur districts, respectively, delivered to the two largest wholesale markets in each district.

Therefore, the ability of aggregation to improve F&V distribution to traditionally neglected markets whilst continuing to coordinate, aggregate and lower farmer costs is currently unknown. We investigate this central question through a system dynamics model (SDM) based on the F&V system of Koilwar block, Bhojpur, located 40 km west of the state capital Patna (Fig. 1). Although the model is informed by data from Koilwar and wider Bihar, every effort has been made to keep the model as generalisable as possible, in order to capture the archetypical dynamics of fringe urban–rural marketing environments commonplace across north India and South Asia.

### 2.2 Data

System dynamics models represent systems as structures of stocks, flows, and feedbacks (Sterman, 2000), with links between model variables expressed as ordinary differential equations. Given their ability to explicitly capture feedback loops, delays and nonlinearities (Turner, Menendez, Gates, Tedeschi, & Atzori, 2016), systems models are increasingly being utilised in the investigation of complex food system challenges, including the design of inclusive value chain interventions (Lie et al., 2018, Queenan et al., 2020), disease outbreak management (Galarneau et al., 2020, Mumba et al., 2017), and the links between agricultural resilience and food security (Herrera de Leon and Kopainsky, 2019, Nicholson et al., 2020, Stave and Kopainsky, 2015). Here, our quantitative systems model was informed by five datasets (Table 1); the extent to which these datasets provide reliable and representative information is evaluated in Appendix A.

**Table 1:** Overview of the datasets informing model design and evaluation, including the specific elements of the model informed by each dataset.

Dataset	Format	Period	Size	Role in model design and evaluation
Rapid value chain analysis	Qualitative/primary	March – September 2018	49 interviews with actors in F&V production, marketing and consumption	- Structures of actors, trade and governance underlying the F&V system of Bihar - Decision-making processes of farmers and traders when purchasing and selling F&V
Loop dashboard – Koilwar block	Quantitative/secondary	October 2016 – September 2018	46,291 individual market transactions	- Daily time-series of Loop (i) membership, (ii) farmers opting to supply Loop, (iii) market sales - Loop transport costs
Farmer-household surveys	Quantitative/primary	February 2019	360 farming households (120 Loop and 240 non-Loop)	- Loop and non-Loop: (i) market participation, (ii) consumption of own produce, (iii) waste, (iv) F&V given away, (v) production costs - Average agricultural land cost
Value chain surveys	Quantitative/primary	February 2019	28 surveys with actors in F&V marketing	- Market trader: (i) capacities, (ii) marketing routines, (iii) marketing costs, (iv) wastage rates in transit, (v) price variations with F&V quality - Commission rates
Spatial group model building (SGMB)	Quantitative and qualitative/ primary	January – April 2019	10 sessions with up to 12 stakeholders each: five farmers, two aggregators, one commission agent, two distance traders, one wholesaler, and one retailer	- Drivers of (i) Loop adoption/dis-adoption, (ii) Loop daily participation, (iii) market choice - Loop aggregator capacities - Non-Loop market transport costs - Time-series of market traders - Number of retail consumers and their purchase routines - Scenario feasibility (Section 2.4)

First, a rapid value chain analysis aimed to discover the actors, decision-making processes and governance structures underlying the F&V system. Semi-structured interviews with farmers and aggregators focused on the marketing activities of farmers, plus any recent changes associated with the introduction of aggregation. Interviews with commission agents, wholesalers, retailers, and consumers focused on the movement of F&V within and downstream of markets, including the decisions at each stage of the system *vis-à-vis* availability, quality and prices (Table 1).

Second, the ‘Loop dashboard’ (www.loopapp.org/loop/analytics) provides a near-real time record of the transactions between aggregators and market traders. Since 2016, the dashboard has recorded the types of F&V sold, quantities, and revenues of over 700,000 transactions, alongside meta-datasets detailing the costs of aggregation, and the locations of villages and markets. We subset the dashboard to the 46,291 transactions from Koilwar to inform model design and evaluation (Table 1).

Third, we conducted 360 farmer-household surveys in Bhojpur and Muzaffarpur districts to increase the volume of quantitative data available, including estimates of land ownership, F&V production and input costs (Table 1). These two districts were purposefully sampled as Loop aggregation had been ongoing for at least one year prior to the study in both districts, thus covering the reference periods of our household surveys. Within each district, to associate outcomes with the type of market supplied, we first used dashboard data to split the markets into ‘large’ and ‘small’ (i.e. above or below the mean Loop supply per market). One market was then randomly sampled from each size category per district, followed by a randomly sampled village serving each market. The four Loop villages were then paired with nearby villages that had not supplied Loop at least once in the year before the survey, but exhibited similar market access (i.e. distance, travel times and costs) and transport infrastructure (i.e. road accessibility and quality) to their respective Loop village. Overall, 120 Loop and 240 non-Loop farm-households were surveyed – with half of the non-Loop households located in Loop villages and the other half located in non-Loop villages.

Fourth, we conducted quantitative surveys with traders in Koilwar block (Bhojpur) and Minapur block (Muzaffarpur) to obtain information on market capacities, commissions and trading costs (Table 1) in both urban and local markets. The surveys covered the cross-section of active traders, ranging from wholesalers that export up to 2000 kg/day to neighbouring states, to retailers that sell around 300 kg/day to local consumers.

Parallel to the growing recognition for the importance of contextual information in systems modelling (Rich, Rich, & Dizyee, 2018), we also conducted a series of 10 spatial group model building (SGMB) sessions in Bhojpur and Muzaffarpur. Building upon traditional group modelling frameworks (Vennix, Andersen, & Richardson, 1997), SGMB employs innovative approaches, such as the Layerstack offline GIS framework (Rich et al., 2018), to co-develop understanding around problematic behaviours in the system. The aims, agenda, and timings of each session were planned in advance, with each session lasting up to three hours and involving a range of stakeholders from across the F&V system (Table 1). Ultimately, SGMB helped to further establish the feedbacks and decision-making processes that drive Loop participation and day-to-day marketing choices.

### 2.3 Model description and evaluation

The SDM was developed in the modelling software STELLA Architect (ISEE Systems) to capture the Loop aggregation scheme within its wider F&V production and marketing system (Cooper et al., 2021).^1^ Each simulation runs at half-day timesteps for 11 years, in order to capture both short-term marketing decisions which play out over days and weeks, and longer-term, multi-annual trends in production and consumption. In order to keep the parameterisation and evaluation datasets independent, the model was parameterised over the first 302 half-days (i.e. October 2017 – February 2018), before evaluation over the next 366 half-days (i.e. March – August 2018). Future scenarios (Section 2.4) then activate from October 2018 and run for the next decade (September 2028) to assess the effects of alternative interventions on outcomes of interest. Consistent with our exploration of total F&V availability and affordability, the volumes of all 47 F&V types detailed in the Loop dashboard are aggregated to represent one F&V stock. Here we provide a brief overview of the model’s characteristics and structures, with model documentation and evaluation available in Appendices A-E.

**Fig. 2:**
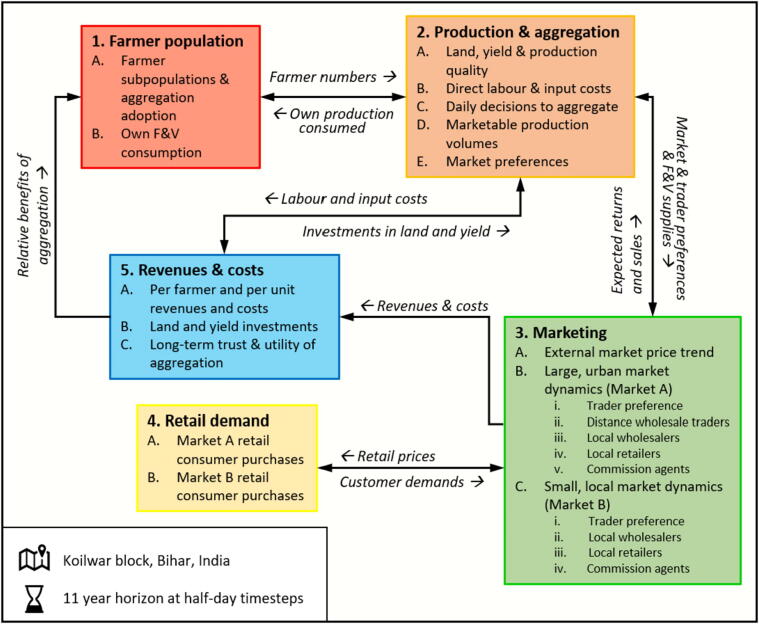
The overarching structure of the model, including its constituent modules, processes and flows of information and material (black arrows). The full model description is available in Appendix E

The model is formed of five interacting modules (Fig. 2) and includes two farming subpopulations and two F&V marketing environments. ‘Market A’ represents an urban wholesale environment with a peak capacity of 150,000–200,000 kg/day, whilst Market B represents a local cluster of five smaller markets with a peak capacity of 15,000–20,000 kg/day. In turn, the total population of farm owners in Koilwar is set to 12,087, revised up from the latest figure available in Census of India (2011) using the annual population growth rate of Bihar. As per Loop dashboard data for October 2017, the initial number of farmers participating in aggregation is set to 90 (i.e. 0.7% of the model population) – growing to 1100 farmers after one year as Loop actively recruited farmers through extension efforts (Appendix D, Fig. S3). In turn, the remaining farmers (‘non-Loop’) are yet to adopt aggregation and must self-supply the market. The number of farmers adopting aggregation is driven by active extension efforts and the benefits of aggregation spreading via word-of-mouth (Bass, 1969). The effectiveness of each of these pathways is modified by the relative returns and guaranteed sales (i.e. expected proportion of market supply that will be successfully sold) of aggregation, and the trust in aggregation to provide these benefits month-on-month (Appendix E, Module A).

The model also captures various processes and decisions underpinning F&V availability in local markets: (i) day-to-day participation in aggregation, (ii) market choices, and (iii) trader choices at each market. Each decision is based upon the expected guaranteed sales and expected profitability at each stage of the system, producing a chain of feedbacks that drive F&V flows downstream to traders and consumers, and flows of information back upstream to farmers regarding the expected returns and guaranteed sales under each supply pathway.

F&V production flows downstream to Market A or Market B (Fig. 2), minus the proportions (i) consumed by farm households, (ii) given away, and (iii) wasted prior to the farmgate (Appendix E, Module A). The aggregation supplies sent to either market is driven by the expected marketing returns and guaranteed sales of each market over the last week (Appendix E, Module B). Similarly, non-Loop supply decisions are dependent upon their expected marketing returns and guaranteed sales at each market, albeit smoothed over the last month to reflect the inferior mobility of self-supplying farmers. Therefore, reflecting our SGMB discussions and evidence from similar developing food system contexts (Anjalay and Bhamoriya, 2011, Cadilhon et al., 2005, Vandeplas et al., 2013), farmers and traders build relationships over time as a result of successful transactions. In turn, these relationships may then resist farmers opportunistically switching their supply destinations, meaning even if Market B was to exhibit higher prices in the short-run, farmers might still prefer to supply Market A given the experience accrued.

The choice of traders at Market A, the urban wholesale market, is between: (i) inter-state ‘distance wholesale traders’, only operating in the morning with capacities of 2000 kg/trader/half-day, (ii) local wholesalers with capacities of 400 kg/trader/half-day, and (iii) local retailers with capacities set by retail consumer demands. Due to the absence of distance traders, the choice in Market B is between local wholesalers and retailers only (Appendix E, Module C). Trader numbers on any half-day are parameterised with time-series co-developed with SGMB participants; traders may then enter or exit the market depending on the profitability of trading over the last month (Appendix E, Module C). The prices paid by market traders for produce are driven by three factors (Appendix E, Module C): (i) the volume of F&V available to each trader, relative to their downstream demand (Sterman, 2000); (ii) trader expectations of prices when selling F&V; and (c) produce quality. The expected price of distance traders is a timeseries generated by the weighted average weekly price at wholesale markets in Patna (October 2017 – September 2018) (NHB, 2018) of the four crops making up more than 50% of total Loop aggregations (i.e. brinjal, cauliflower, cabbage and bitter gourd arrivals) – repeated over the course of the simulation (Appendix, Module C). During SGMB, distance traders were identified as the major source of price information; therefore, all other traders base their expected price on the price of distance traders.

With regards to produce quality, the task of distinguishing between ‘high’ and ‘low’ quality supplies at the farmgate is rarely practiced in Bihar (Kumari et al., 2017). Instead, our SGMB discussions highlighted that F&V grading is one of the traditional responsibilities of market commission agents. Therefore, F&V quality is treated as an exogenous variable, with the proportion of F&V production that is high-grade ranging randomly between 60 and 100%. From here, the relative proportions of high-grade (receiving full price) and low-grade (receiving half price) supplies produce a weighted average price per market trader (Appendix E, Module B).

At the furthest point downstream, F&V purchases by consumers in Markets A and B are dependent upon retail prices (Sterman, 2000). Each consumer is assumed to purchase F&V from markets twice per week, with Market A serving 15,000 households, and Market B serving 10,000 households, as estimated during SGMB.^2^ Initial per consumer demands for F&V are parameterised to satisfy 5.5 household members, each consuming the latest estimated average urban and rural consumption levels for Bihar of 177 g/capita/day (Market A) and 159 g/capita/day (Market B) (NSSO, 2013), respectively.

The average returns per farmer population are calculated by subtracting the half-daily costs of production inputs, hired labour, market transport and commission from the market revenues (Appendix E, Module B).^3^ Consistent with dashboard data, aggregating farmers incur transport costs of 0.75–0.85 Rs/kg to Market A, and 1.00–1.10 Rs/kg to Market B; the costs of self-supplying either market vary randomly between 1.00 and 1.50 Rs/kg on any given half-day. From here, the accumulation of profits enables farmers to invest in F&V production at the start of each cropping season. In the absence of reliable quantitative data, we conservatively assume farmers only invest if their cumulative profits are adequate and growing (Appendix E, Module E). If both conditions are met, farmers purchase an area equivalent to 20% of their cumulative profits. Likewise, farmers may invest in inputs to enhance yields if their profits smoothed over the last season are increasing. These investments feedback to increase production by 1.5–2.5%/year in the baseline scenario – consistent with Government of India. (2018) (2018) data for Bihar (2013–2018). Finally, production growth is counterbalanced by the assumption that consumer populations increase by 2.3%/year – as per the Bihar-wide rate of population growth from 2001 to 2011 in the latest Census of India (2011).

We conducted three evaluation tests (Appendix A–C) to assess the extent to which the model captures reality.^4^ Whilst the model replicates the broad historical trends of participating farmers and aggregation sales (Appendix B), we also conducted a two-stage Monte Carlo sensitivity analysis to assess model robustness under multiple interacting uncertainties (Cooper and Dearing, 2019, Spear and Hornberger, 1980) (Appendix C).

All variables informed by data were first assigned a reliability score based on the transferability, quantity and statistical confidence of the underlying data (Table S1) (Chapman & Darby, 2016). Variables scoring beneath the reliability threshold (n = 54) were then perturbed by up to ±25% of their parameterised values across 500 simulations, with Kolmogorov-Smirnov tests (Massey, 1951) identifying variables with significant influence on model dynamics. Overall, 14 variables were found to be critically sensitive (Table S2), corresponding to the initial proportion of non-Loop supplies to Market A, six weighting factors in Loop market and trader choices, and seven variables moderating the sensitivity of market prices to changes in supply.

**Fig. 3:**
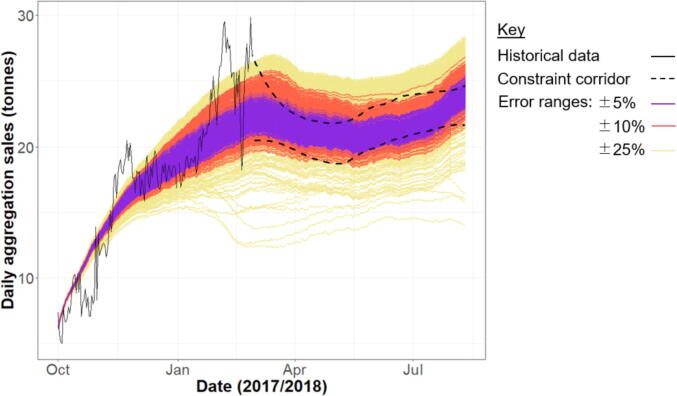
Outputs from the second stage sensitivity analysis. The 14 critically sensitive variables (Table S2) were perturbed by three different error ranges, and the resulting timeseries of aggregated sales in Market A are compared to the 95% confidence intervals (dashed) of the observed data from March – August 2018.

The critically sensitive variables were then perturbed by three permissible error ranges (±5%, ±10% and ±25% of their parameterised values) across 500 simulations per subset to ascertain whether historical trends could still be replicated under the interacting errors of the most uncertain parameters (Cooper & Dearing, 2019). Overall, 94.8% of outcomes under the 5% error range fall within the quantitative constraint corridor, followed by 79.1% and 47.1% for the ±10% and ±25% error subsets, respectively (Fig. 3). Whilst the similarity between simulations and observations weakens as errors widen (Fig. 3), the model critically captures the historical patterns of growth, stabilisation and seasonality in market sales across all three error ranges.

In summary, we have evaluated the reliability of all datasets informing model design (Appendix A) and shown that the model recreates the broad historical trends of three key system indicators (Appendix B). Furthermore, Monte Carlo sensitivity analysis has built confidence in the ability of the model to robustly reproduce historical reference patterns under multiple and interacting error terms (Fig. 3).

### 2.4 Scenarios and outcomes

The model described and evaluated above provides a virtual laboratory to explore how aggregation may increase the distribution of F&V to smaller, more local markets, whilst simultaneously avoiding farmer-facing trade-offs and maintaining the financial sustainability of the aggregation scheme. The rationale and operationalisation of 14 future scenarios are described below (Table 2).

**Table 2:** Future scenarios exploring how aggregation may be positioned to increase F&V availability in smaller markets, whilst simultaneously avoiding negative farmer-facing trade-offs.

Group	Scenario name	Scenario description and operation in the model
Baseline	Counterfactual	What if aggregation stopped evolving? The number of aggregation participants remains constant from Oct. 2018 to Sept. 2028
	A. Baseline	Baseline evolves naturally; there are no constraints on aggregation adoption via word-of-mouth, daily participation or market supply

Internal	B. Scaling-up Loop	On top of the baseline, extension efforts continue to actively recruit new aggregation participants. The number of new farmers recruited through extension is set to 1/10 of the value during the initial period of extension (Oct. 2017 – Feb. 2018)
	C. Loop subsidy	Aggregation transport costs to Market B subsidised at 50%
	D. Fixed Market B quota	20% of the daily aggregated volume is supplied to Market B

External	E. Market B transport costs	Baseline Loop and non-Loop transport costs, and their respective wastage rates (5% of farmgate F&V volumes) are halved to reflect improvements in transport infrastructure
	F. Retail demand growth	Reflecting an exogenous increase in F&V demand from current demands (i.e. 3.1 kg/consumer/market-visit), the price beyond which consumers in Market B perceive F&V to be expensive is increased by 5%/year, as per the increase in the real minimum wage in Bihar from 2012 to 2019 (Government of Bihar, 2019)
	G. Market B cold storage	Traders in Market B have access to an on-site cold storage chamber of 10,000 kg/day capacity

Combination: scale	H. Scaling-up Loop and reference price increase	What if extension continued *and* F&V demand is externally increased?
	I. Scaling-up and cold storage	What if extension continued *and* traders in Market B had access to cold storage of 10,000 kg/day capacity?
	J. Scaling-up, reference price increase and cold storage	Combination of scenarios ‘H’ and ‘I’: what if aggregation extended at the same time as cold storage and higher F&V demands?
Combination: Market B deficit	K. Deficit quota and reference price increase	What if aggregation reacted to retail supply shortages in Market B *and* F&V demand is externally increased?
	L. Deficit quota and cold storage	What if aggregation reacted to retail supply shortages in Market B *and* traders in Market B had access to cold storage of 10,000 kg/day capacity?
	M. Deficit quota, reference price increase and cold storage	Combination of scenarios ‘K’ and ‘L’: what if aggregation reacted to supply shortages in Market B at the same time as cold storage is available and higher F&V demands?

First, to establish the trade-offs emerging from the unmodified aggregation scheme, the baseline is compared to a counterfactual which holds the number of participating farmers beneath 10% of the total farmer population. In turn, the baseline simulates the unconstrained evolution of the current aggregation scheme over ten years, whereby adoption, day-to-day participation and marketing pathways remain driven only by the relative performance of aggregation, rather than any external influence.

The second group explores how changes to aggregation may generate positive outcomes for Market B consumers and farmers (Table 2). By boosting adoption through active extension efforts, *Scenario B* assesses whether scaling-up aggregation alone can improve the attractiveness of supplying the smaller, more local market environment. *Scenario C* investigates whether a market-specific transport subsidy (i.e. 50% of aggregation transport costs) dampens the risks associated with the smaller market and incentivises aggregations to Market B. In turn, *Scenario D* directly supplies 20% of aggregated volumes towards Market B. Given the feedbacks between profitability, guaranteed sales and participation, *Scenario D* explores the dynamics of farmers understanding during adoption that 20% of their supplies will be delivered to Market B.^5^

The third group of scenarios explore how aggregation interacts with changes to the enabling environment, i.e. the regulatory, sociocultural and infrastructural settings of the system (Reardon, Lu, & Zilberman, 2019) (Table 2). First, investments in new roads and surface improvements are often linked to cheaper transport costs, increased mobility and market access (Beauchamp et al., 2018, Rammelt and Leung, 2017). Whilst a lack of site-specific data prohibits the model from capturing the explicit causal dynamics (e.g. between investments and road quality), *Scenario E* proxies improved transport infrastructure by halving the transport costs to Market B and associated in-transit wastage rates for both farmer subpopulations.

As a proxy for consumers being able to spend more on F&V, for instance, as a result of behaviour changes in response to nutrition education schemes and/or income changes (Hawkes & Ruel, 2008), *Scenario F* simulates the effects of an exogenously increasing demand amongst consumers in Market B. This scenario is modelled as a 5%/year increase in the retail reference price of F&V in Market B, which links the reference consumer demand (Table 2) to a specified initial price (20 Rs/kg). Therefore, an increase in the reference price elevates the threshold price beyond which the elasticity of demand begins to increase and F&V retail prices are perceived to become relatively unaffordable (Sterman, 2000). Finally, *Scenario G* explores the effects of traders in Market B having access to a 10,000 kg/day cold storage chamber, in line with the notion that improving cold storage accessibility may help to reduce food loss rates that are estimated to be up to 40% of total F&V production in northern India (Gardas et al., 2018, Godfray et al., 2010). Given that F&V cold storage facilities are currently only available in major urban markets, Scenario G represents the scaling-out of cold storage to smaller, more local markets, enabling F&V to be stored for up to a maximum of 21 days (as opposed to two days in the baseline). The share of storage between wholesalers and retailers is assumed proportional to their respective F&V supplies, and rent is paid by traders at 0.15 Rs/kg/half-day.

The *combination scenarios* then aim to explore the combinations of interventions improving equity in consumer outcomes, and whether potential trade-offs generated by the standalone scenarios can be overcome with multiple interventions. We first combine the assumption that aggregation schemes inherently aim to reach as many farmers as possible and the growing priority of the Indian government towards cold storage investment, plus the ability of consumers to spend more on F&V as a result of demand stimulation from nutrition education schemes (Table 2). Second, we modify the fixed quota (*Scenario D*) to become reactive to the expected supply shortages of Market B retailers. For instance, if retailers expect daily supplies to be 200 kg short of demand, then the retailers can request aggregation to supply the deficit. This ‘de-seasonalisation’ scenario (Reardon, 2015) is also combined with cold storage and an exogenous increase in demand (Table 2).

Acknowledging that too many outcomes can complicate scenario comparisons (Holden, Linnerud, & Banister, 2017), we track six outcomes summarising changes in F&V availability in Market B (A-B), the livelihood outcomes of farmers (C-D), and the performance and attractiveness of aggregation (E-F):

A. *Monthly average price of F&V in retail Market B –* Main indicator of F&V affordability in Market B.
B. *Monthly total F&V purchases per consumer in retail Market B –* Dependent on the availability and price of F&V in retail Market B.
C. *Monthly average F&V marketing returns per Loop farmer –* Revenues minus transportation costs, commissions, and hired input and labour costs.
D. *Monthly average F&V marketing returns per farmer –* Weighted average of all farmer F&V returns (i.e. Loop and non-Loop).
E. *Monthly average Loop versus non-Loop per unit returns –* Ratio of Loop to non-Loop average per unit returns.
F. *Aggregation costs recovered –* Sum of the service charge collected from participating farmers equal to 10% of the aggregation cost.

**Fig. 4:**
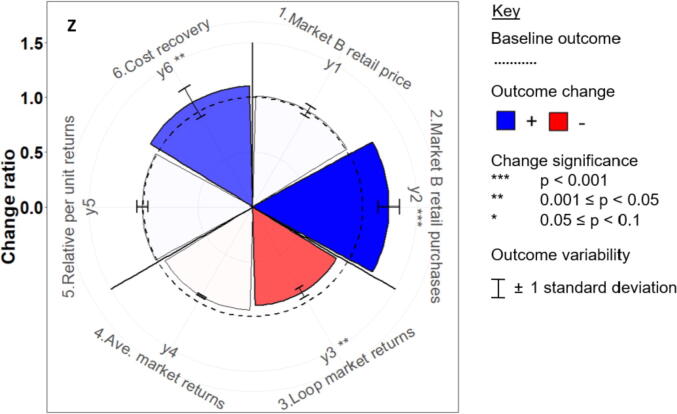
Schematic trade-off wheel comparing the change ratios of the six outcomes (y_1_… y_6_) under the hypothetical future scenario ‘Z’, relative to their respective baseline values. Note: the price outcome is inverted (i.e. a positive change represents cheaper F&V).

We develop novel trade-off wheels to aggregate the monthly output time-series (Appendix D) and analyse the direction, magnitude and significance of change in each outcome (Fig. 4). The above six outcomes populate the radial x-axis, with the mean change ratio of each outcome (i) under each scenario (j) equalling:

(1) $${\mathrm{Change}\;ratio}_{i,j}=mean\left(\frac{{\mathrm{New}\;value}_{i,j,t}}{{\mathrm{Baseline}\;value}_{i,j,t}}\right)$$

where t represents the simulation month.

For example, the hypothetical scenario in Fig. 4 is associated with significant positive changes in monthly small market retail F&V purchases and aggregation costs recovered; however, the returns of participating farmers are significantly degraded relative to the baseline. Lastly, two-sample *t*-tests are performed to determine whether the monthly outcome values under each intervention are significantly different from the baseline.

## 3 Results

### 3.1 Implications of the current aggregation scheme

Before analysing scenarios to improve the equitable distribution of F&V, we establish the temporal dynamics associated with the baseline evolution of aggregation adoption and market supplies, relative to the counterfactual holding aggregation participation below 10% of farmers.

**Fig. 5:**
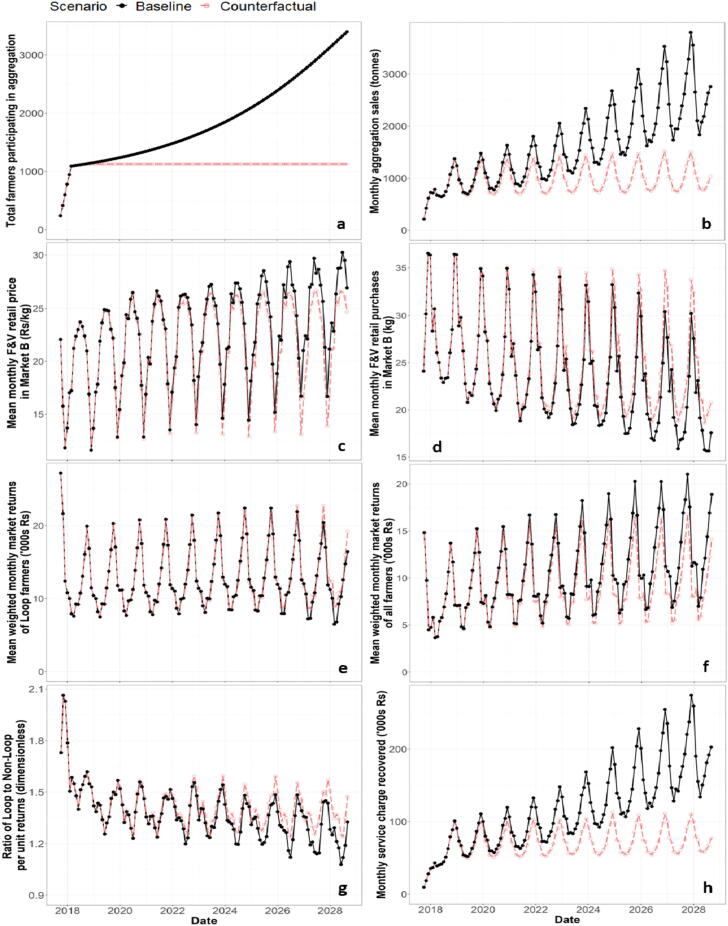
Time-series comparing the baseline to the counterfactual across two drivers (a-b) and six outcomes (c–h): (a) Participating farmers, (b) aggregation sales, (c) retail price of F&V in Market B, (d) monthly F&V purchases per consumer in Market B, (e) participating farmer market returns, (f) all farmer market returns, (g) relative returns from aggregation, and (h) aggregation charges recovered.

Driven by the tripling of participating farmers over ten years (Fig. 5a), the F&V volume aggregated in the final year of the baseline is 2.5 times greater than the counterfactual (Fig. 5b). Moreover, with 2300 more farmers benefitting from lower transport costs and improved access to Market A over the course of the ten years, increased participation is associated with an 15.9% increase in monthly average all farmer market returns (Fig. 5f). However, increasing participation in aggregation gradually diverts F&V supplies towards the urban market, making F&V more expensive for consumers dependent on the smaller local market environment. Relative to the counterfactual, the baseline observes 6.8% fewer farmers supplying Market B each month – underpinning a 5.2% increase in mean monthly retail prices (Fig. 5c) and a 5.5% decrease in mean monthly purchases per consumer in Market B (Fig. 5d).

Comparing the baseline to the counterfactual suggests that simply aggregating an increasing volume of F&V does not automatically produce a F&V system that works for all. Instead, whilst strengthened participation spreads the benefits of lower transportation costs and urban market access across a wider farmer population, the reinforcement of urban-bound F&V supplies reduces local F&V availability and increases the prices faced by consumers in smaller, traditionally neglected retail markets. Consequently, the alternative futures below attempt to identify pathways enabling the simultaneous growth of aggregation and farmer benefits, whilst improving F&V distribution towards smaller local market environments.

### 3.2 Trade-offs of market interventions

**Fig. 6:**
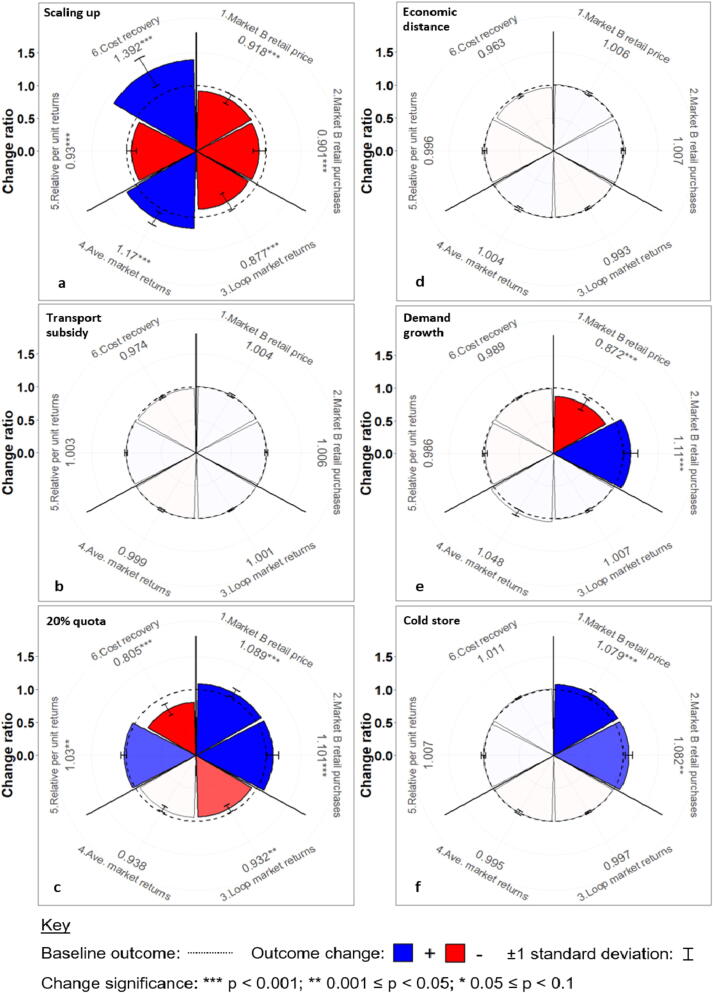
Trade-off wheels resulting from the alternative standalone scenarios: (a) scaling-up aggregation; (b) 50% subsidy of aggregation transport to Market B; (c) 20% of daily aggregated volumes to Market B; (d) the halving of all Market B transport costs and in-transit wastage rates; (e) exogenous increase in Market B F&V demand; (f) cold storage in Market B. See Appendix D for the time-series underlying these plots.

Here we describe the outcome changes and trade-offs associated with the six standalone scenarios (Fig. 6), alongside their key causal dynamics. Overall, significant improvements in local F&V availability *and* affordability are generally associated with improved F&V delivery and/or capture within the local marketing environment.

Scaling aggregation from approximately 1000–5500 farmers over ten years (Fig. 6a) reinforces the trade-offs found between the baseline and counterfactual (Fig. 5). Whilst increasing membership improves average farmer returns and the charges recovered, enhanced farmer-access to Market A reduces the monthly number of farmers supplying Market B by 11.9%. With increasing urban-bound supplies, 3.5% of participating farmers must supply secondary traders in Market A due to the capacity limitations of distance traders (0.2% in the baseline) – triggering a reduction in average returns from aggregation.

The additional 2200 kg/month supplied to Market B under the aggregation subsidy scenario only produces marginal improvements in small market F&V availability (Fig. 6b). Whilst the monthly number of aggregating farmers supplying Market B increases by 4.8% relative to the baseline, non-Loop farmers adapt to an increase in small market crowding by increasing their monthly supplies to Market A by 7.6%. Conversely, the scenario that explicitly diverts aggregated supplies (~42,000 kg/month or +275% above the baseline) away from Market A produces significant improvements in consumer outcomes in Market B (Fig. 6c). However, the average returns of farmers participating in aggregation are traded-off, with the saturation of the local market with aggregated produce causing the average price farmers receive to fall by 5.1% and the proportion of unsold supplies to increase by 8.3%, relative to the baseline. The fixed quota scenario also weakens the aggregation charges recovered (Fig. 6c), with the need to send 20% of aggregated supplies towards the smaller, less profitable market reducing aggregation adoption by 27.9% over 10 years (Appendix D, Fig. S3).

Halving both Loop and non-Loop transport costs and wastage rates to Market B delivers an extra 2000 kg/month. However, the standalone scenario is only able to generate marginal benefits for retail customers in Market B (Fig. 6d), with only an additional 5.7% Loop and 1.2% of Loop and non-Loop farmers, respectively, encouraged to supply Market B each month. In turn, cold storage in Market B is the only scenario to significantly improve both local retail purchases and prices without significant trade-offs across farmer- and scheme-facing dimensions (Fig. 6f). Enabling retailers to store F&V up to 21 days cuts daily in-market wastage from 17.0% to 13.7%, which is associated with a reduction in the mean monthly retail price from 22.8 Rs/kg (‘baseline’) to 20.9 Rs/kg. Contrastingly, exogenously boosting demand is found to bolster the resilience of local retail consumer purchases to seasonally higher prices (Fig. 6e). Consequently, retailers adapt their prices and margins to heightened demands, meaning higher purchases are counterbalanced by prices averaging 14.0% above the baseline.

### 3.3 Combinations of interventions to overcome multidimensional trade-offs

Increasing F&V delivery and capture within local markets can generate consumer-facing benefits. However, to avoid trade-offs, changes in marketing dynamics must be sensitive to local market capacities and demands, and the inherent preferences of farmers to supply less risky, larger markets. Reflecting the importance of multiple instrument studies in environmental economics and social-ecological systems (Cooper and Dearing, 2019, Goulder and Parry, 2008), and in line with recent calls to action in the exploration of food systems (Kawabata et al., 2020, Mylona et al., 2018), we combine interventions to uncover potential synergies in farmer and small market consumer outcomes.

**Fig. 7:**
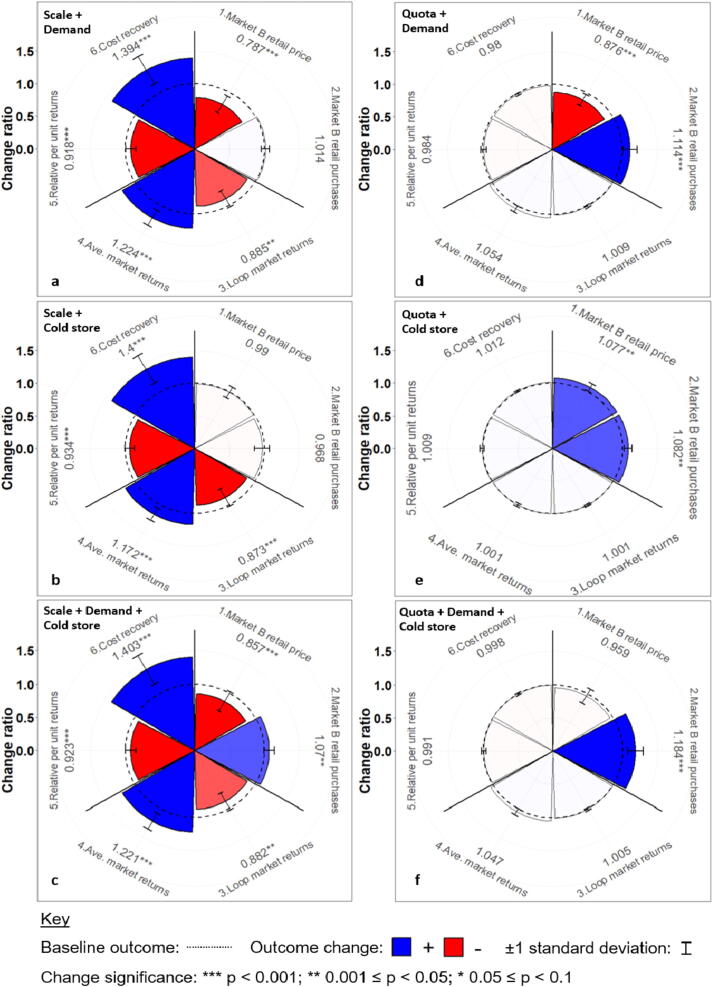
Trade-off wheels resulting from the six combination scenarios (Table 2). See Appendix D for the time-series underlying these plots.

Combining the increase in F&V demand or the introduction of cold storage with aggregation scaling can overcome the significant purchase declines in Market B associated with scaling-up aggregation alone (Fig. 7a). Whilst scaling-up aggregation reduces the monthly number of farmers supplying Market B by 10.7%, the additional effect of exogenously increasing F&V demand in Market B enables consumers to maintain purchases, despite higher prices caused by the diversion of F&V towards the urban market (Fig. 7a). In turn, combining scaling-up and cold storage cuts Market B wastage by 37.6% (relative to the baseline) (Fig. 7b), which helps to offset significant consumer-facing trade-offs. The combination of scaling-up, increasing demand and cold storage produces significant improvements in purchases by Market B consumers (+8.0%, Fig. 7c); however, cold storage is unable to overcome the synergistic effects of increasing urban-bound supplies and the acceptance of higher prices amongst consumers, meaning consumers pay +12.8% above the baseline to satisfy heightened demands.

The trade-offs across the wider outcomes reflect the dominance of scaling-up over the two external interventions (Fig. 7c). Whilst participating farmer returns increase by 2.0% over the scaling-up scenario alone, the combination of Market B cold storage and an exogenous increase in demand are unable to offset the significant decline in participant returns relative to the baseline, caused by access to Market A becoming less exclusive.

In turn, the three combinations involving a small market quota that adapts to retailer supply shortages all produce significant improvements in small market F&V purchases from the baseline. For instance, combining the dynamic quota with an exogenously increasing consumer demand (Fig. 7d) produces significantly higher consumer retail purchases, despite retail prices that are +10.6% above the baseline. In turn, the combination of cold storage and a dynamic quota is the only scenario that produces at least marginal improvements across all outcomes (Fig. 7e). Retail prices in Market B are dampened by 8.4% and purchases increase by 9.4% above the baseline, whilst diverting aggregated supplies only during local supply shortages benefits both aggregating and self-supplying farmers by avoiding Market B oversupply. Lastly, combining cold storage and the dynamic quota with an exogenously increasing consumer demand produces the largest overall improvement in monthly F&V purchases (20.4%), whilst avoiding the significantly higher prices associated with other exogenous demand scenarios (Fig. 7f). Synergistic effects are particularly prominent during the monsoon offseason (Appendix D, Fig. S3), with the adaptive delivery of aggregation and the storage of F&V within the local market limiting the inflation of prices caused by more demanding consumers.

## 4 Discussion

This study has identified future scenarios that harness F&V aggregation to increase the availability of F&V in traditionally neglected smaller market environments, whilst protecting against trade-offs on farmer returns and the costs recovered from aggregation. We discuss the implications of these futures in the contexts of (i) developing aggregation as a means of making food systems work for all, (ii) the F&V policy landscape of Bihar, and (iii) the limitations of our approach.

### 4.1 Developing consumer-sensitive aggregation schemes

Food systems in developing country contexts are typically characterised by fragmented supplies, underdeveloped infrastructures and a lack of market transparency. Consequently, efforts to boost F&V supplies and livelihoods have predominantly focused on the links between smallholder farmers and premium market channels involving consumers with higher purchasing powers. Market innovation in horticulture across South Asia has thus been associated with a rapid increase in formal producer groups and the entrance of organised retail outlets into traditional food systems (Reardon, Timmer, & Minten, 2012).

Given the above, interventions to enhance F&V supplies to traditionally neglected local markets in locations such as rural Bihar must operate within an increasingly urban-oriented environment. Aggregation may reinforce the barriers associated with local capacity limitations and relative economic distances, with participating farmers benefiting from heightened mobility, the sharing of transport costs and the ability to satisfy the demands of urban wholesalers. Therefore, our study further supports the notion that innovations in developing food systems may actually present double-edged swords (Glover and Poole, 2019, Rammelt and Leung, 2017), with a subset of farmers benefitting disproportionately at the expense of F&V availability and prices faced by consumers in smaller market environments. Problematically for the identification of such trade-offs in real-time, the divergence between farmer and smaller market consumer outcomes is not immediately obvious; for instance, under the *scaling-up* scenario, a 1.2%/year decrease in F&V volumes arriving at the local market ultimately produces a 19.1% decline in the volumes purchased by local consumers after eight years.

On the flipside, explicitly developing aggregation to improve equitable F&V distribution has multiple potential pitfalls. Whilst diverting a fixed supply towards local markets significantly improves F&V availability, such a static approach underappreciates feedbacks involving farmer profits, local demands, and the long-term reputation of the scheme itself. Making aggregation more responsive to seasonal production volumes, market capacities and price variations presents opportunities to overcome problems associated with offseason supply shortages, peak-season oversupply and wastage. However, positioning aggregation schemes to ‘de-seasonalise’ (Reardon, 2015) F&V systems is complicated by the potential need to establish new trust-based relationships between aggregators and local traders, and develop efficient methods of forecasting and communication in an environment lacking reliable market information.

Owing to the feedbacks, delays and trade-offs linking different sectors of food systems, it is increasingly recognised that the achievement of multiple outcomes across food systems may require combinations of interventions that target actors with different goals, interests and scales (Kawabata et al., 2020, Reardon et al., 2019). To this end, our systems-level trade-off analysis shows that combinations of interventions that significantly improve F&V availability in traditionally neglected markets whilst avoiding negative impacts upon the financial takeaways of farmers must be carefully designed, multipronged and coordinated, as to avoid reinforcing pre-existing trade-offs that concentrate benefits towards a small number of stakeholders.

Here, combinations that achieve multidimensional outcomes go beyond the traditional first step of generating a supply response (e.g. through increased participation and subsidisation) to address a number of structural barriers underpinning the food system (Ogutu and Qaim, 2019, Schreinemachers et al., 2018). The introduction of cold storage allows F&V that might otherwise be wasted to be stored in the market – building a reserve against supply shortages and spiky demands. A self-sustaining feedback mechanism then stabilises prices and boosts local F&V demands, which increases the attractiveness of the local market to farmers and aggregators. In turn, combining an exogenously stimulated consumer demand (e.g. by enabling consumers to spend more on F&V through behaviour change and/or an income effect) with an adaptable, targeted supply of aggregated F&V ahead of short-term supply shortages helps to further enhance the volumes available, as well as the returns of farmers both inside and outside of the aggregation scheme.

### 4.2 Policy implications

State and national F&V policy discussions in India over the past decade have centred around trade liberalisation, including strengthening the connections between farmers and large markets, improving transport and cold chain investments, and increasing the market shares of organised retailers (Government of India, 2020, NITI Aayog, & Rocky Mountain Institute, 2018). As highlighted by Choudhury et al., 2020, Khandelwal et al., 2020, India’s F&V policy is largely farmer-focused, commercially oriented and not explicitly designed to be nutrition-sensitive. Against this backdrop, we highlight a number of policy relevant implications for developing aggregation whilst ensuring already neglected market segments are not further left behind.

Funded by the World Bank, The Bihar Rural Livelihoods Programme (‘JEEViKA’) is the flagship rural development programme of the Government of Bihar. Through the sharing of input costs and aggregation of agricultural produce, JEEViKA primarily aims to enhance the socioeconomic empowerment of farmers in Bihar (Datta, 2015). However, as with Loop, JEEViKA primarily focuses on enabling participants to ‘reach out to larger markets’ (Shetty, Vutukuru, Kak, Penumaka, & Takada, 2017, p. 107). Therefore, with aggregation becoming an increasingly central strategy of The Bihar Transformative Development Project (i.e. JEEViKA Stage II) (Shetty et al., 2017), this study timely stresses the potential benefits (and trade-offs) of broadening the scope of traditionally farmer-focused interventions to become increasingly sensitive towards the needs of relatively neglected markets.

To this end, an important policy implication arising from our results is that strategies to lower the cost of delivery to smaller markets, such as transport cost subsidies or road upgrades, may have limited success by themselves. This is in contrast to successful policies that financially incentivise farmers to larger markets in similar developing country contexts (Rammelt and Leung, 2017, Zanello et al., 2019). Thus, this study highlights a potential case of policy resistance, as financial incentives amounting to around 100 Rs per farmer per market day may be insufficient to overcome the comparative pull of the generally better priced, lower risk urban market environment.

Rather than subsidies or quotas to increase direct F&V delivery, the introduction of small-scale market-based cold storage significantly reduces F&V wastage rates and retail prices. However, despite the number of cold storage units in Bihar increasing by 67% between 2000 and 2011, cold storage in Bihar is “almost exclusively used for the storage of potato” (Minten, Reardon, Singh, & Sutradhar, 2011, p .5), with F&V facilities only available in hub markets like Bihar Sharif and Muzaffarpur. Therefore, whilst our findings are timely given the planned national increase in cold storage investment (Government of India, 2020), their realisation requires the diversification of national and state-level policies from the industrial-scale towards more locally managed cold chains, which extend the life of perishable F&V within the local market environment. However, operationalisation would inherently involve multiple financial and logistical challenges, including the funding of cold chamber construction and maintenance, provision of rural power supplies, and potential disruptions to local value chain governance.

In parallel, interventions that drive F&V demand consistently generate positive changes in local retail purchases, whilst avoiding the need for significant investments in physical infrastructure. Yet, the operationalisation of such scenarios faces two key barriers. First, relative to the prioritisation of F&V production and marketing within the national policy space, behaviour change communication remains largely the remit of non-governmental organisations, private actors, and government departments external to the agricultural sector, including the Ministry of Health and Family Welfare (MoHFW), and Ministry of Women and Child Development (MoWCD) (Khandelwal et al., 2020). Second, actions that stimulate demand, such as nutrition education and awareness campaigns or increased income, may lead to significantly higher local prices without complementary increases in F&V supply. Therefore, whilst targeted strategies such as cash transfer schemes may boost F&V demand amongst disadvantaged consumers (Choudhury et al., 2020), the stimulation of demand should be complemented with additional instruments that increase F&V delivery and storage to avoid widening inequalities between those who can afford F&V and those who cannot.

In summary, and in line with Thow et al., 2018, Khandelwal et al., 2020, we recommend a package of complementary policy levers involving multiple food system stakeholders and sectors to utilise the ability of aggregation to bulk and lower costs of F&V supplies to market, whilst at the same time adapting the enabling environment to improve demand and capture within the local environment to reduce the risks of wastage, insufficient market capacities and erratic prices on both farmers and consumers.

### 4.3 Evaluation of the systems approach

Systems approaches are increasingly recognised for their ability to incorporate quantitative and qualitative data, dynamic feedback structures and interlinkages between segments of food systems that are traditionally analysed in isolation (Kawabata et al., 2020, Mylona et al., 2018). Whilst providing a virtual laboratory to horizon-scan interventions increasing local F&V availability, the key limitations of our approach must also be recognised.

First is the level of process aggregation and abstraction required to tractably and efficiently simulate system-wide dynamics. While disaggregating between the production volumes, transport costs and revenues of participating and non-participating farmers, the analysis lacked sufficient quantitative evidence to distinguish between different land classes and cropping patterns within each farmer subpopulation. Similarly, the consumption impacts that we model pertain to a generic group of ‘consumers’ that are distinct from ‘farmers’ in the model set-up. Of course, Loop farmers are also consumers; however, we currently do not include the F&V consumption impacts arising from improved incomes amongst the Loop farming group. Loop farmers are only a small proportion of the local population of consumers served by specific markets, but it is nevertheless worth keeping in mind that income effects on consumption are not currently included. Likewise, retail consumers in each market are assumed to have homogenous demands, both in terms of the quantity and types of F&V purchased, whilst farmgate intermediaries that may occasionally purchase from farmers and transport produce to markets have been excluded in the interests of model parsimony due to the lack of reliable data.

Therefore, building upon the trade-offs and policy implications established here, a future research priority could seek to down-scale to a more disaggregated analysis of the differential effects of market interventions and household-level pathways (e.g. empowerment of female decision-making and time-use) on producer versus consumer trade-offs. Likewise, in line with growing recognition for the importance of such granular perspectives, future research could break down consumption impacts on the basis of socio-economic categories (Choudhury et al., 2021, Hemalatha et al., 2020). Such additions to the model structure would require further cross-sectional and participatory datasets that interrogate the key differences between producer and consumer subgroups, albeit with potentially negative implications for the complexity of the model, and the scalability and interpretability of findings beyond the specific case study.

Second, the number of marketing pathways parameterised during model initialisation remain constant. Therefore, the model does not currently explore plausible scenarios where farmers seek more distant markets to those traditionally supplied, perhaps during sustained periods of regionally low prices or the potential emergence of e-commerce. With these boundaries in mind, our results should be interpreted in the context of two alternative marketing pathways, whereby changes in aggregation and the wider environment influence the dynamics of traders and consumers along each pathway, which then feedback to influence regional marketing choices and farmer-facing outcomes.

Finally, our outcomes do not attempt to cover every potential food system trade-off. Conscious that too many targets risk underperformance in one dimension being discounted for overperformance in others (Holden et al., 2017), our analysis incorporates a number of potentially important outcomes into six key metrics; for example, market transport costs are incorporated into farmer market returns, while market wastage rates contribute to F&V availability and prices. Therefore, in parallel with the rational to further disaggregate system dynamics and feedbacks, future research may aim to increase the number of key outcomes to identify trade-offs across the system at a higher resolution.

## 5 Conclusions

Despite global recognition for the need to make food systems work for all, farmer-focused interventions traditionally prioritise market access and returns over the co-production and equitable distribution of consumer-oriented outcomes. To help address this imbalance, we identify a range of scenarios that enhance the availability of F&V in local market environments in Bihar, India, whilst avoiding trade-offs on farmer returns and participation.

Scaling-up participation in aggregation in developing country contexts can significantly improve farmer market returns and transport costs. However, the approach represents a double-edged sword, with improved urban market access traded-off against the availability and affordability of F&V in more local, traditionally neglected market environments. In contrast, directly increasing aggregated supplies to the local market has significant positive implications for local F&V delivery, but risks trading-off participation in aggregation and higher market wastage. Instead, scenarios that significantly increase local F&V availability whilst avoiding farmer-facing trade-offs simultaneously tackle a number of the multidimensional challenges facing traditional F&V systems, including adapting aggregation to de-seasonalise supply and demand in local markets, the reduction of market wastage through the introduction of small-scale cold storage, and the stimulation of consumer demands through actions such as awareness campaigns or increased incomes. Therefore, this study stresses that win–win futures for farmers and consumers in developing country contexts may not be achievable with a silver-bullet intervention; consequently, we call for the greater appreciation of potential intervention combinations to identify synergistic futures that overcome farmer versus consumer trade-offs in food systems.

## CRediT authorship contribution statement

**G.S. Cooper:** Conceptualization, Methodology, Validation, Formal analysis, Investigation, Writing - original draft, Writing - review & editing. **B. Shankar:** Conceptualization, Methodology, Investigation, Supervision, Funding acquisition, Writing - original draft, Writing - review & editing. **K.M. Rich:** Conceptualization, Methodology, Investigation, Writing - original draft, Writing - review & editing. **N.N. Ratna:** Writing - review & editing. **M.J. Alam:** Writing - review & editing. **N. Singh:** Writing - review & editing. **S. Kadiyala:** Writing - review & editing.

## Declaration of Competing Interest

The authors declare that they have no known competing financial interests or personal relationships that could have appeared to influence the work reported in this paper.

## Footnotes

1 The STELLA model file is available on request from the corresponding author.

2 Study results (Section 3.2 and Section 3.3) remain robust when the half-daily consumer demands in Markets A and B factor in the demands of farming households that have already consumed some of their own produce (Appendix E.2). Overall, the 72 outcome ratios (6 outcomes across 12 scenarios) change by a mean absolute percentage error of 0.652%, with only five of the 72 outcome ratios changing in significance level (see Appendix E.2 for details).

3 Study results also remain robust when the imputed costs of family labour and owned productive assets are included. Overall, the 72 outcome ratios (6 outcomes across 12 scenarios) change by a mean absolute percentage error of 0.837%, with none of the outcome ratios changing in statistical significance (see Appendix E.3 for details).

4 We also assessed the extent to which the prices Loop farmers receive in Markets A and B are cointegrated, both in reality (i.e. Loop dashboard data) and in the model. In both cases, the price trends of both markets are cointegrated with one cointegrating vector (see Appendix E.4 for details).

5 The two transport-oriented interventions (i.e. *Scenario C* and *Scenario E)* only impact market transportation costs and wastage rates, and their associated feedbacks with aggregation participation and market choice. Therefore, secondary factors that might further influence the volumes of F&V deliveries to the smaller market, such as improved road capacities or the willingness of farmers to experience the improved road surfaces, are currently omitted.
