# Supplementary material for: Can fruit and vegetable aggregation systems better balance improved producer livelihoods with more equitable distribution?
Source: World Dev. 2021 Dec;148:105678. doi: 10.1016/j.worlddev.2021.105678 (PMC8520944; doi:10.1016/j.worlddev.2021.105678)
Supplement: Supplementary Data 1 [file mmc1.docx]

**Supplementary information**

Contents

**Appendix A: Qualitative parameter assessment**

**Appendix B: Behaviour reproduction**

**Appendix C: Monte Carlo sensitivity analysis**

**Appendix D: Outcome time-series**

**Appendix E: Model archive**

E.1 Model overview

E.2 Module A: Farmer population

E.3 Module B: Production & aggregation

E.4 Module C: Marketing

E.5 Module D: Retail demand

E.6 Module E: Farmer revenues & costs

**Appendix references**

Appendix A: Qualitative parameter assessment

| **Table S1: Output table of the qualitative parameter assessment. Based on the methodology of Chapman and Darby (2016), each model parameter informed by data was scored out of three for its ‘locational transferability’, ‘spatial scale transferability’, ‘quantity of evidence’, and ‘statistical confidence’. The reliability score for each parameter equals the average scored across the four dimensions. The mean average reliability score equals 58.4% (n = 139) and the standard deviation equals 21.4%. The reliability threshold score equals 37.0% (i.e. mean minus standard deviation); variables that undergo sensitivity analysis (scoring below the threshold or variables informed by personal intuition) are highlighted in red. ‘Dmnl’ = dimensionless number; ‘Rs’ = Indian rupee** | | | | | | |
| --- | --- | --- | --- | --- | --- | --- |
| **Module** | **Sub-module** | **Model variable** | **Value (unit)** | **Source(s)** | **Information type** | **Score (%)** |
| **A. Farmer population** | Farmer sub-populations | Extension on | On = 1; off = 0 (dmsnl) | SGMB sessions; Loop dashboard data; personal intuition | Expert stakeholder knowledge; modeller intuition | 83 |
|  |  | Extension effectiveness baseline | 0.00018 (dmnl) | Bass (1969); Loop dashboard data | Modeller intuition; statistical information | 67 |
|  |  | Adoption baseline | 0.0001 (dmnl) | Bass (1969); Loop dashboard data | Modeller intuition; statistical information | 67 |
|  |  | Contact rate | 2 (people/half-day) | Bass (1969); Loop dashboard data; Household surveys | Modeller intuition; statistical information | 67 |
|  |  | Koilwar farming population | 12,087 (people) | Indian census 2011 | Statistical information | 67 |
|  |  | Market supply frequency | See Table S2 | Household surveys | Statistical information | 75 |
|  |  | Base disadoption rate | 0.2 (people/half-day) | Loop dashboard data | Statistical information | 58 |
|  | Own F&V consumption | Baseline Loop household day consumption of own produce | 0.380 kg/half-day | Household surveys | Statistical information | 75 |
|  |  | Baseline Non-Loop household day consumption of own produce | 0.331 kg/half-day | Household surveys | Statistical information | 75 |
| **B. Production & aggregation** | On-farm costs | Loop on-farm costs (x7) | Variable (see Appendix E.3) | Household surveys | Statistical information | 75 |
|  |  | Loop labour costs (x7) | Variable (see Appendix E.3) | Household surveys | Statistical information | 75 |
|  |  | Non-Loop on-farm costs (x7) | Variable (see Appendix E.3) | Household surveys | Statistical information | 75 |
|  |  | Non-Loop labour costs (x7) | Variable (see Appendix E.3) | Household surveys | Statistical information | 75 |
|  | Loop land & yield | Rabi F&V yield (part of seasonal yield) | See Table S6 | NIFTEM (2013) and NHB (2015) | Statistical information | 50 |
|  |  | Zaid F&V yield (part of seasonal yield) | See Table S6 | NIFTEM (2013) and NHB (2015) | Statistical information | 50 |
|  |  | Kharif F&V yield (part of seasonal yield) | See Table S6 | NIFTEM (2013) and NHB (2015) | Statistical information | 50 |
|  |  | Loop random self-supply | 0 – 5 (%) | SGMB sessions; personal intuition | Expert stakeholder knowledge; modeller intuition | 83 |
|  |  | Minimum Loop quality | 60 (%) | SGMB sessions; personal intuition | Expert stakeholder knowledge; modeller intuition | 83 |
|  |  | Maximum Loop quality | 100 (%) | SGMB sessions; personal intuition | Expert stakeholder knowledge; modeller intuition | 83 |
|  |  | Loop quality smooth time | 14 (half-days) | SGMB sessions; personal intuition | Expert stakeholder knowledge; modeller intuition | 83 |
|  |  | Staple price | See Table S6 | USDA (2019) | Statistical information | 58 |
|  |  | Staple price perception time | 240 (half-days) | Personal intuition | Modeller intuition | 33 |
|  |  | Staple land weighting | -0.00001 (dmnl) | Personal intuition | Modeller intuition | 33 |
|  |  | F&V land weighting | 0.00001 (dmnl) | Personal intuition | Modeller intuition | 33 |
|  | Loop marketable | On farm wastage rate | 4.5 (%) | Household surveys | Statistical information | 75 |
|  |  | F&V given away rate | 2.5 (%) | Household surveys | Statistical information | 75 |
|  |  | Loop wastage en route to market | 5 (%) | SGMB sessions | Expert stakeholder knowledge | 83 |
|  | Short-term satisfaction in Loop | Satisfaction perception time | 14 (half-days) | SGMB sessions | Expert stakeholder knowledge | 83 |
|  | Loop market preference | Market A cost per km | 130 (Rs) | SGMB sessions; Loop dashboard data | Expert stakeholder knowledge; statistical information | 83 |
|  |  | Market A distance | 10 (km) | Map-based data; SGMB sessions | Statistical information; expert stakeholder knowledge | 75 |
|  |  | Market B cost per km | 180 (Rs) | SGMB sessions; Loop dashboard data | Expert stakeholder knowledge; statistical information | 83 |
|  |  | Market B distance | 10 (km) | Map-based data; SGMB sessions | Statistical information; expert stakeholder knowledge | 75 |
|  |  | Aggregation default capacity | 2000 (kg) | SGMB sessions; Loop dashboard data | Expert stakeholder knowledge; statistical information | 83 |
|  |  | Smooth capacity time | 60 (half-days) | SGMB sessions; personal intuition | Expert stakeholder knowledge; modeller intuition | 58 |
|  |  | Aggregator capacity standard deviation | 900 (kg) | SGMB sessions; Loop dashboard data | Expert stakeholder knowledge; statistical information | 83 |
|  |  | Market preference change time | 14 (half-days) | SGMB sessions; personal intuition | Expert stakeholder knowledge; modeller intuition | 83 |
|  |  | Market sales weight | 0.5 (dmnl) | Personal intuition | Modeller intuition | 33 |
|  |  | Market profits weight | 0.1 (dmnl) | Personal intuition | Modeller intuition | 33 |
|  |  | Random market preference change | -5.0 – 5.0 (%) | SGMB sessions | Expert knowledge | 83 |
|  | Non-Loop land & yield | Minimum non-Loop quality | 60 (%) | SGMB sessions; personal intuition | Expert stakeholder knowledge; modeller intuition | 83 |
|  |  | Maximum non-Loop quality | 100 (%) | SGMB sessions; personal intuition | Expert stakeholder knowledge; modeller intuition | 83 |
|  |  | Non-Loop quality smooth time | 14 (half-days) | SGMB sessions; personal intuition | Expert stakeholder knowledge; modeller intuition | 83 |
|  | Non-Loop marketable | On farm wastage rate | 4.5 (%) | Household surveys | Statistical information | 75 |
|  |  | F&V given away rate | 2.5 (%) | Household surveys | Statistical information | 75 |
|  |  | Maximum non-Loop Market A proportion | 99 (%) | Personal intuition | Modeller intuition | 33 |
|  |  | Default non-Loop wastage rate en route to market | 5 (%) | SGMB sessions | Expert stakeholder knowledge | 83 |
| **C. Market A dynamics** | External market | External market price | See Table S6 | National Horticultural Board (NHB, 2018) ‘MIS Weekly Report’ dataset | Statistical information | 67 |
|  | Trader preference | Loop and non-Loop price perception time | 14 (half-days) | Personal intuition | Modeller intuition | 33 |
|  |  | Loop and non-Loop trust perception time | 60 (half-days) | Personal intuition | Modeller intuition | 33 |
|  |  | Non-Loop minimum proportion to distance traders in Market A | 0.1 (dmnl) | Personal intuition | Modeller intuition | 33 |
|  |  | Non-Loop maximum proportion to distance traders in Market A | 0.8 (dmnl) | Personal intuition | Modeller intuition | 33 |
|  |  | Non-Loop minimum proportion to local traders in Market A | 0.8 (dmnl) | Personal intuition | Modeller intuition | 33 |
|  |  | Non-Loop maximum proportion to local traders in Market A | 0.9 (dmnl) | Personal intuition | Modeller intuition | 33 |
|  | Distance wholesale trader | Distance traders | See Table S6 | SGMB sessions | Expert stakeholder knowledge | 83 |
|  |  | Distance trader capacity | 2000 (kg) | SGMB sessions; Value chain surveys | Expert stakeholder knowledge; statistical information | 75 |
|  |  | Distance trader frequency | 2 (half-days) | Value chain surveys | Statistical information | 75 |
|  |  | Market A wastage levy | 5 (%) | Value chain surveys | Statistical information | 67 |
|  |  | Distance trader wastage en route | 5 (%) | Value chain surveys | Statistical information | 67 |
|  |  | Inventory coverage perception time | 14 (half-days) | Value chain analysis and SGMB | Expert stakeholder knowledge | 83 |
|  |  | Sensitivity of price to inventory coverage | - 0.3 (dmnl) | Sterman (2000); personal intuition | Modeller intuition | 33 |
|  |  | Sensitivity of price to costs | - 0.1 (dmnl) | Sterman (2000); personal intuition | Modeller intuition | 33 |
|  |  | Trader cost perception time | 14 (dmnl) | Personal intuition | Modeller intuition | 33 |
|  |  | Quality price difference | 50 (%) | SGMB sessions; Value chain surveys | Expert stakeholder knowledge; statistical information | 75 |
|  |  | Profit perception time | 60 (half-days) | Personal intuition | Modeller intuition | 33 |
|  |  | Distance trader effect of profits on capacity | See Figure S15 | Personal intuition | Modeller intuition | 33 |
|  |  | Gaddidar outside actor commission | 8.0 (%) | SGMB sessions; Value chain surveys | Expert stakeholder knowledge; statistical information | 75 |
|  |  | Gaddidar local actor commission | 5.0 (%) | SGMB sessions; Value chain surveys | Expert stakeholder knowledge; statistical information | 75 |
|  | Local wholesale trader | Local traders | See Table S6 | SGMB sessions | Expert stakeholder knowledge | 83 |
|  |  | Local trader capacity | 400 (kg) | SGMB sessions; Value chain surveys | Expert stakeholder knowledge; statistical information | 75 |
|  |  | Local trader frequency | 2 (half-days) | Value chain surveys | Statistical information | 75 |
|  |  | Local trader wastage en route | 5 (%) | Value chain surveys | Statistical information | 67 |
|  |  | Inventory coverage perception time | 14 (half-days) | Value chain analysis and SGMB | Expert stakeholder knowledge | 83 |
|  |  | Sensitivity of price to inventory coverage | - 0.3 (dmnl) | Sterman (2000); personal intuition | Modeller intuition | 33 |
|  |  | Sensitivity of price to costs | - 0.1 (dmnl) | Sterman (2000); personal intuition | Modeller intuition | 33 |
|  |  | Quality price difference | 50 (%) | SGMB sessions; Value chain surveys | Expert stakeholder knowledge; statistical information | 75 |
|  |  | Profit perception time | 60 (half-days) | Personal intuition | Modeller intuition | 33 |
|  |  | Local trader effect of profits on capacity | See Figure S15 | Personal intuition | Modeller intuition | 33 |
|  | Local retailer | Retailers | See Table S6 | SGMB sessions | Expert stakeholder knowledge | 83 |
|  |  | Local retailer frequency | 1 (half-day) | Value chain surveys | Statistical information | 75 |
|  |  | Inventory coverage perception time | 14 (half-days) | Value chain analysis and SGMB | Expert stakeholder knowledge | 83 |
|  |  | Sensitivity of price to inventory coverage | - 0.3 (dmnl) | Sterman (2000); personal intuition | Modeller intuition | 33 |
|  |  | Sensitivity of price to costs | - 0.1 (dmnl) | Sterman (2000); personal intuition | Modeller intuition | 33 |
|  |  | Quality price difference | 50 (%) | SGMB sessions; Value chain surveys | Expert stakeholder knowledge; statistical information | 75 |
|  |  | Profit perception time | 60 (half-days) | Personal intuition | Modeller intuition | 33 |
|  |  | Retailer effect of profits on capacity | See Figure S15 | Personal intuition | Modeller intuition | 33 |
| **D. Market B dynamics** | Trader preference | Price perception time | 14 (half-days) | Personal intuition | Modeller intuition | 33 |
|  |  | Trust perception time | 60 (half-days) | Personal intuition | Modeller intuition | 33 |
|  |  | Non-Loop minimum proportion to local traders in Market B | 0.2 (dmnl) | Personal intuition | Modeller intuition | 33 |
|  |  | Non-Loop maximum proportion to local traders in Market B | 1 (dmnl) | Personal intuition | Modeller intuition | 33 |
|  | Local wholesale trader | Local traders | See Table S6 | SGMB sessions | Expert stakeholder knowledge | 83 |
|  |  | Local trader capacity | 400 (kg) | SGMB sessions; Value chain surveys | Expert stakeholder knowledge; statistical information | 75 |
|  |  | Local trader wastage en route | 5 (%) | Value chain surveys | Statistical information | 67 |
|  |  | Market B wastage levy | 5 (%) | Value chain surveys | Statistical information | 67 |
|  |  | Inventory coverage perception time | 14 (half-days) | Value chain analysis and SGMB | Expert stakeholder knowledge | 83 |
|  |  | Sensitivity of price to inventory coverage | - 0.3 (dmnl) | Sterman (2000); personal intuition | Modeller intuition | 33 |
|  |  | Sensitivity of price to costs | - 0.1 (dmnl) | Sterman (2000); personal intuition | Modeller intuition | 33 |
|  |  | Quality price difference | 50 (%) | SGMB sessions; Value chain surveys | Expert stakeholder knowledge; statistical information | 75 |
|  |  | Profit perception time | 60 (half-days) | Personal intuition | Modeller intuition | 33 |
|  |  | Local trader effect of profits on capacity | See Figure S15 | Personal intuition | Modeller intuition | 33 |
|  | Local retailer | Retailers | 50 (people) | SGMB sessions | Expert stakeholder knowledge | 83 |
|  |  | Inventory coverage perception time | 14 (half-days) | Value chain analysis and SGMB | Expert stakeholder knowledge | 83 |
|  |  | Sensitivity of price to inventory coverage | - 0.3 (dmnl) | Sterman (2000); personal intuition | Modeller intuition | 33 |
|  |  | Sensitivity of price to costs | - 0.1 (dmnl) | Sterman (2000); personal intuition | Modeller intuition | 33 |
|  |  | Quality price difference | 50 (%) | SGMB sessions; Value chain surveys | Expert stakeholder knowledge; statistical information | 75 |
|  |  | Profit perception time | 60 (half-days) | Personal intuition | Modeller intuition | 33 |
|  |  | Retailer effect of profits on capacity | See Figure S15 | Personal intuition | Modeller intuition | 33 |
|  | Gaddidar commission | Gaddidar local actor commission | 5.0 (%) | SGMB sessions; Value chain surveys | Expert stakeholder knowledge; statistical information | 75 |
| **E. Retail demand** | Market A demand | Demand adjustment delay | 14 (half-days) | Personal intuition | Modeller intuition | 33 |
|  |  | Reference industry demand elasticity | 0.92 (dmnl) | Kumar et al. (2011); Kumari and Singh (2016) | Statistical information | 50 |
|  |  | Maximum consumption | 7.7 (kg) | Personal intuition | Modeller intuition | 33 |
|  |  | Reference price | 20 (Rs/kg) | Personal intuition; SGMB sessions | Modeller intuition; Expert stakeholder knowledge | 33 |
|  |  | Reference consumer demand | 3.4 (Rs/kg) | NSSO (2013); Rich and Dizyee (2016) | Statistical information | 83 |
|  |  | Market A consumer routine | 1 in 7 (half-days) | SGMB sessions; value chain analysis report | Expert stakeholder knowledge | 66 |
|  |  | Initial total Market A consumer households | 15,000 | SGMB sessions | Expert stakeholder knowledge | 83 |
|  |  | Consumer population growth | 2.3 (%/year) | Indian census 2011 | Statistical information | 67 |
|  | Market B demand | Demand adjustment delay | 14 (half-days) | Personal intuition | Modeller intuition | 33 |
|  |  | Reference industry demand elasticity | 0.88 (dmnl) | Kumar et al. (2011); Kumari and Singh (2016) | Statistical information | 50 |
|  |  | Maximum consumption | 7.7 (kg) | Personal intuition | Modeller intuition | 33 |
|  |  | Initial reference price | 20 (Rs/kg) | Personal intuition | Modeller intuition | 33 |
|  |  | Reference consumer demand | 3.1 (kg) | NSSO (2013); Rich and Dizyee (2016) | Statistical information | 83 |
|  |  | Market B consumer routine | 1 in 7 (half-days) | SGMB sessions; value chain analysis report | Expert stakeholder knowledge | 66 |
|  |  | Total Market B consumer households | 10,000 | SGMB sessions | Expert stakeholder knowledge | 83 |
| **F. Revenues and costs** | Per unit and per farmer revenues | Loop yield investment rate | 0.2 (dmnl) | Personal intuition | Modeller intuition | 33 |
|  |  | Loop yield investment conversion | 1% yield increase per Rs 20,000 invested | Personal intuition | Modeller intuition | 33 |
|  |  | Loop land investment rate | 0.2 (dmnl) | Personal intuition | Modeller intuition | 33 |
|  |  | Non-Loop transport cost Market A | 1.0 – 1.5 (Rs/kg) | SGMB sessions; value chain analysis report | Expert stakeholder knowledge | 66 |
|  |  | Non-Loop transport cost Market B | 1.0 – 1.5 (Rs/kg) | SGMB sessions; value chain analysis report | Expert stakeholder knowledge | 66 |
|  |  | Non-Loop yield investment rate | 0.2 (dmnl) | Personal intuition | Modeller intuition | 33 |
|  |  | Non-Loop yield investment conversion | 1% yield increase per Rs 20,000 invested | Personal intuition | Modeller intuition | 33 |
|  |  | Non-Loop land investment rate | 0.2 (dmnl) | Personal intuition | Modeller intuition | 33 |
|  |  | Price of one tenth of a katha (land area) | 7,600 (Rs) | Household surveys | Statistical information | 67 |
|  |  | Baseline roving non-Loop proportion | 10 (%) | SGMB sessions | Expert stakeholder knowledge | 83 |
|  | Trust and utility | Trust perception time | 60 (half-days) | SGMB sessions; personal intuition | Expert stakeholder knowledge; modeller intuition | 75 |
|  |  | Loop utility perception time | 60 (half-days) | Personal intuition | Modeller intuition | 33 |
|  |  | Market profit utility weight | 0.5 (dmnl) | Personal intuition | Modeller intuition | 33 |
|  |  | Sales utility weight | 1 (dmnl) | Personal intuition | Modeller intuition | 33 |
|  |  | Loop profitcweight in trust | 0.5 (dmnl) | Personal intuition | Modeller intuition | 33 |
|  |  | Loop sales weight in trust | 1 (dmnl) | Personal intuition | Modeller intuition | 33 |

Appendix B: Behaviour reproduction

| 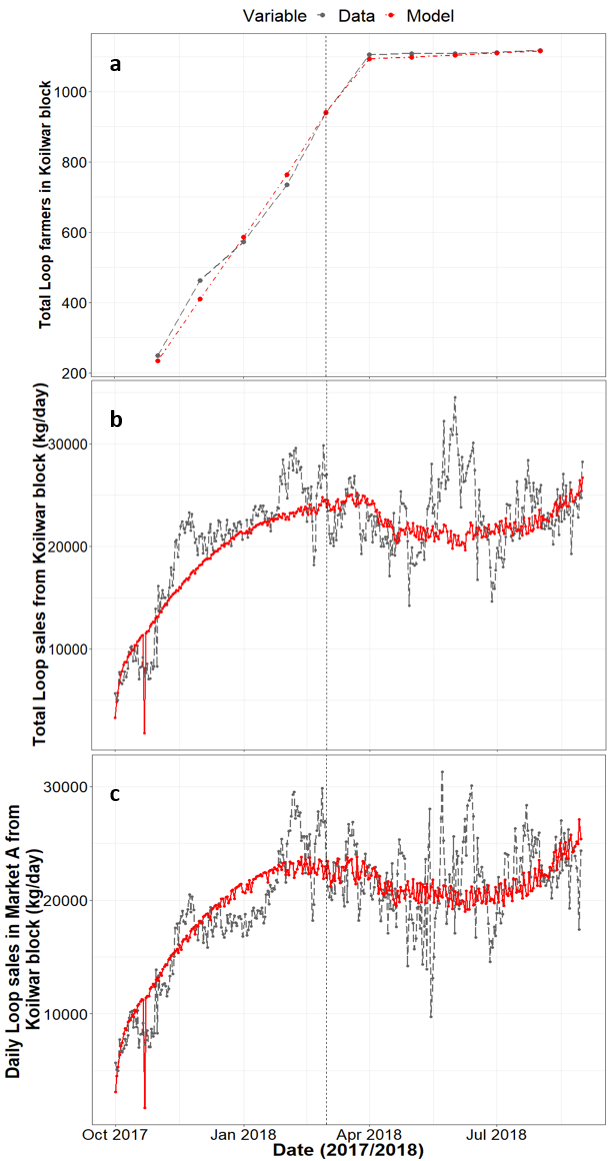 |
| --- |
| Figure S1: Time-series comparing modelled outputs to Loop dashboard data: (a) the number of Loop farmers in Koilwar block, (b) the total quantity of Loop sales from Koilwar block (i.e. Market A and Market B), and (c) the quantity of Loop F&V supplied to Market A from Koilwar block. The dashed line separates the parameterisation period (left) from the evaluation period (right). |

Appendix C: Monte Carlo sensitivity analysis

| **Table S2: Outputs from the first stage Monte Carlo sensitivity analysis, where each of the variables which scored lower than the threshold reliability score (Table S1) were randomly varied by ±25% of their parameterised values across 500 simulations. The process was repeated on a per-module basis, before Kolmogorov-Smirnov tests were performed to identify the variables within each module with significant differences between the error ranges producing ‘behaviour-giving’ outputs and the error ranges producing ‘non-giving’ outputs. ‘Behaviour-giving’ outputs are classified as 95% of outputs falling between the 95% confidence intervals of a locally weighted least squares regression (LOESS) of daily aggregated supplies to Market A from March 1^st^ – August 25^th^ 2018 (Figure S2)**. ‘**Critically sensitive’ variables (p < 0.05) then go onto the second stage Monte Carlo analysis, as detailed in the main manuscript (Section 2.3).** | | | | | |
| --- | --- | --- | --- | --- | --- |
| **Module** | **Variable** | **Variable ID** | **K-S Score (D)** | **p-value** | **Critically sensitive** |
| Production & aggregation | Staple price perception time (in land allocation) | B1 | 0.097 | p > 0.1 | 🗶 |
|  | Staple land weighting (in land allocation) | B2 | 0.065 | p > 0.1 | 🗶 |
|  | F&V land weighting (in land allocation) | B3 | 0.054 | p > 0.1 | 🗶 |
|  | Market sales weight (in Loop market choice) | B4 | 0.278 | p < 0.001 | ✓ |
|  | Market returns weight (in Loop market choice) | B5 | 0.196 | p < 0.001 | ✓ |
|  | Non-Loop Market A proportion | B6 | 0.558 | p < 0.001 | ✓ |
| Market A dynamics | Loop and non-Loop price perception time (in trader preference) | C1 | 0.060 | p > 0.1 | 🗶 |
|  | Loop and non-Loop trust perception time (in trader preference) | C2 | 0.072 | p > 0.1 | 🗶 |
|  | Non-Loop minimum proportion to distance traders in Market A | C3 | 0.058 | p > 0.1 | 🗶 |
|  | Non-Loop maximum proportion to distance traders in Market A | C4 | 0.0867 | p > 0.1 | 🗶 |
|  | Non-Loop minimum proportion to local traders in Market A | C5 | 0.158 | 0.001 < p < 0.05 | ✓ |
|  | Non-Loop maximum proportion to local traders in Market A | C6 | 0.420 | p < 0.001 | ✓ |
|  | Sensitivity of price to distance trader inventory coverage | C7 | 0.088 | p > 0.1 | 🗶 |
|  | Sensitivity of price to distance trader costs | C8 | 0.107 | p > 0.1 | 🗶 |
|  | Cost perception time | C9 | 0.083 | p > 0.1 | 🗶 |
|  | Distance trader profit perception time | C10 | 0.071 | p > 0.1 | 🗶 |
|  | Distance trader effect of profits on capacity | C11 | 0.064 | p > 0.1 | 🗶 |
|  | Sensitivity of price to local trader inventory coverage | C12 | 0.277 | p < 0.001 | ✓ |
|  | Sensitivity of price to local trader costs | C13 | 0.058 | p > 0.1 | 🗶 |
|  | Local trader profit perception time | C14 | 0.072 | p > 0.1 | 🗶 |
|  | Local trader effect of profits on capacity | C15 | 0.158 | 0.001 < p < 0.05 | ✓ |
|  | Sensitivity of price to retailer inventory coverage | C16 | 0.311 | p < 0.001 | ✓ |
|  | Sensitivity of price to retailer costs | C17 | 0.116 | 0.05 < p < 0.1 | 🗶 |
|  | Retailer profit perception time | C18 | 0.801 | p > 0.1 | 🗶 |
|  | Retailer effect of profits on capacity | C19 | 0.170 | 0.001 < p < 0.05 | ✓ |
| Market B dynamics | Price perception time (in trader preference) | D1 | 0.052 | p > 0.1 | 🗶 |
|  | Trust perception time (in trader preference) | D2 | 0.084 | p > 0.1 | 🗶 |
|  | Non-Loop minimum proportion to local traders in Market B | D3 | 0.672 | p < 0.001 | ✓ |
|  | Non-Loop maximum proportion to local traders in Market B | D4 | 0.069 | p > 0.1 | 🗶 |
|  | Sensitivity of price to local trader inventory coverage | D5 | 0.123 | p > 0.1 | 🗶 |
|  | Sensitivity of price to local trader costs | D6 | 0.088 | p > 0.1 | 🗶 |
|  | Local trader profit perception time | D7 | 0.071 | p > 0.1 | 🗶 |
|  | Local trader effect of profits on capacity | D8 | 0.595 | p < 0.001 | ✓ |
|  | Sensitivity of price to retailer inventory coverage | D9 | 0.161 | p < 0.05 | ✓ |
|  | Sensitivity of price to retailer costs | D10 | 0.067 | p > 0.1 | 🗶 |
|  | Retailer profit perception time | D11 | 0.116 | p > 0.1 | 🗶 |
|  | Retailer effect of profits on capacity | D12 | 0.048 | p > 0.1 | 🗶 |
| Retail demand | Demand adjustment delay in Market A | E1 | 0.084 | p > 0.1 | 🗶 |
|  | Maximum consumption in Market A | E2 | 0.100 | p > 0.1 | 🗶 |
|  | Reference price in Market A | E3 | 0.111 | 0.05 < p < 0.1 | 🗶 |
|  | Demand adjustment delay in Market B | E4 | 0.083 | p > 0.1 | 🗶 |
|  | Maximum consumption in Market B | E5 | 0.107 | p > 0.1 | 🗶 |
|  | Reference price in Market B | E6 | 0.750 | p < 0.001 | ✓ |
| Revenues and costs | Loop yield investment rate | F1 | 0.036 | p > 0.1 | 🗶 |
|  | Loop yield investment conversion | F2 | 0.066 | p > 0.1 | 🗶 |
|  | Loop land investment rate | F3 | 0.062 | p > 0.1 | 🗶 |
|  | Non-Loop yield investment rate | F4 | 0.514 | p > 0.1 | 🗶 |
|  | Non-Loop yield investment conversion | F5 | 0.077 | p > 0.1 | 🗶 |
|  | Non-Loop land investment rate | F6 | 0.071 | p > 0.1 | 🗶 |
|  | Loop utility perception time | F7 | 0.071 | p > 0.1 | 🗶 |
|  | Market returns utility weight | F8 | 0.071 | p > 0.1 | 🗶 |
|  | Sales utility weight | F9 | 0.046 | p > 0.1 | 🗶 |
|  | Loop market returns weight in trust | F10 | 0.099 | p > 0.1 | 🗶 |
|  | Loop sales weight in trust | F11 | 0.567 | p < 0.001 | ✓ |

| 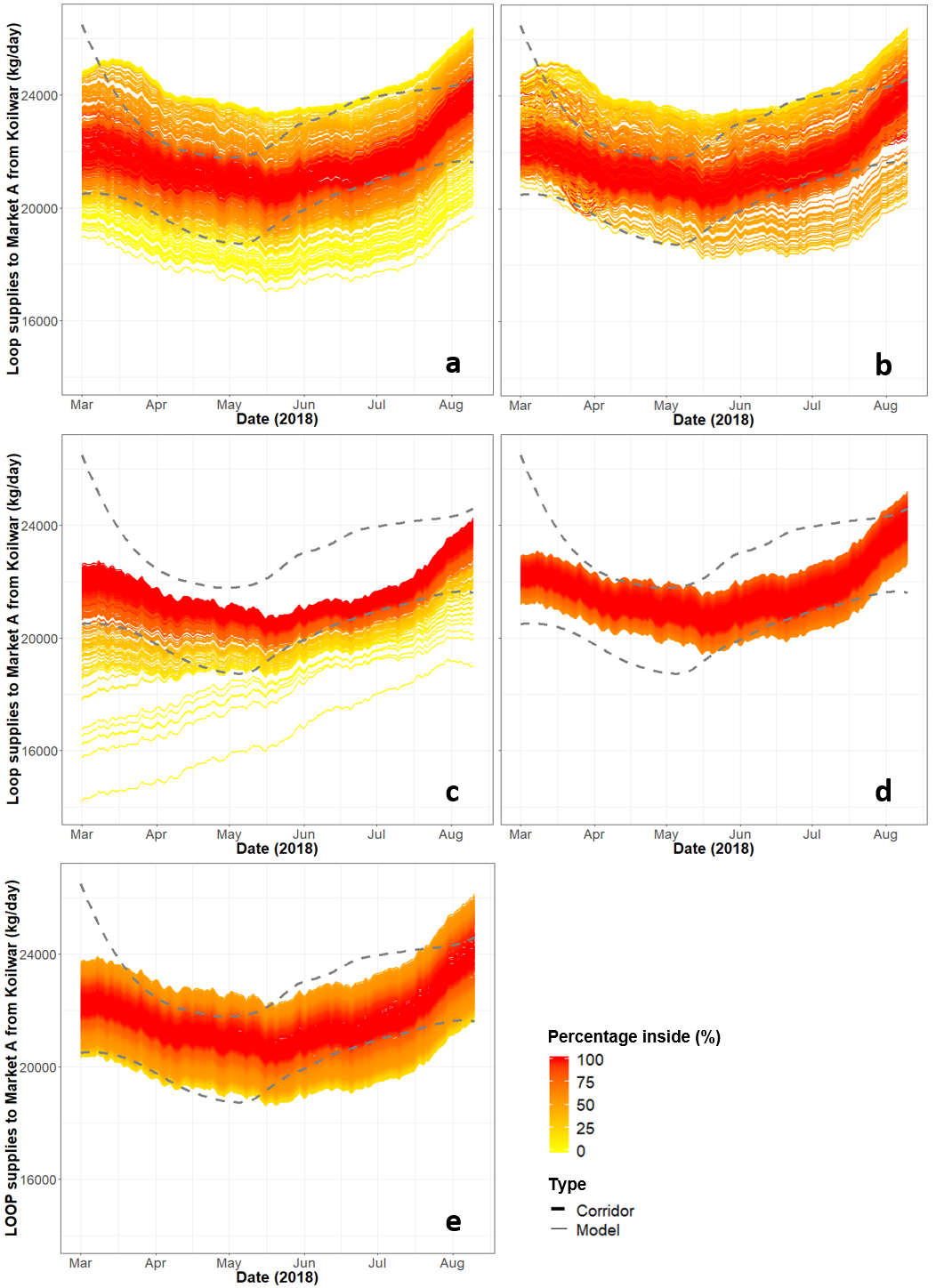 |
| --- |
| Figure S2: Modular output time-series from the first stage of Monte Carlo analysis. Module names and associated percentage of runs that are ‘behaviour-giving’ (i.e. 95% of outcomes fall within the constraint corridor): (a) Production & aggregation (54.5%), (b) Market A dynamics (84.7%), (c) Market B dynamics (90.9%), (d) Retail demand (90.1%), (e) Revenues and costs (75.2%). |

Appendix D: Outcome time-series

| 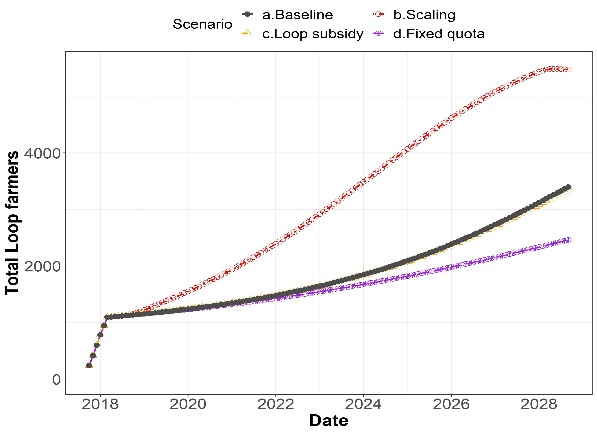 | 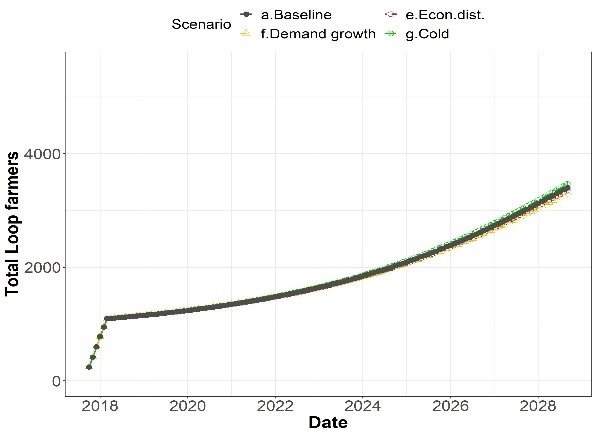 |
| --- | --- |
| 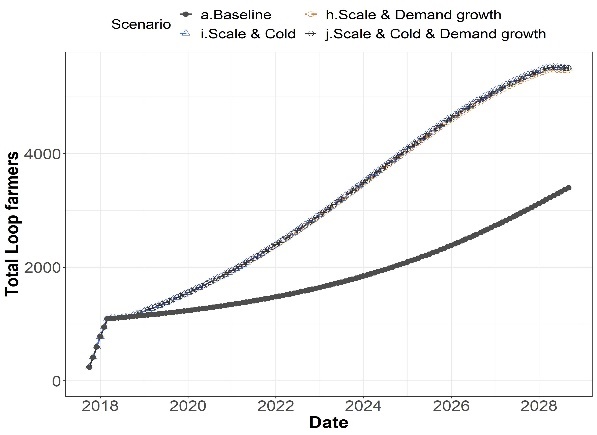 | 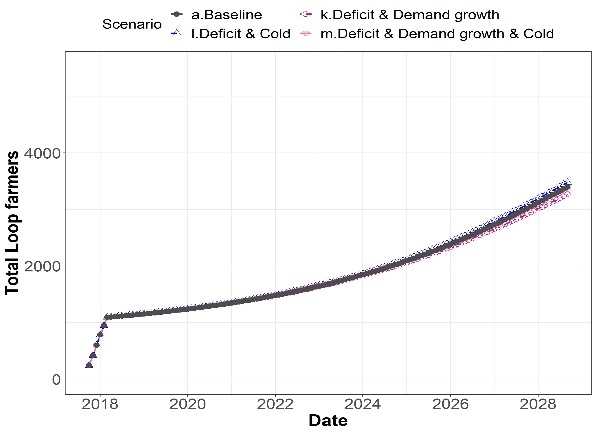 |
| Figure S3: Time-series of total Loop farmers under the four scenario groups; (top left) Internal, (top right) external, (bottom left) Combination scale, (bottom right) combination Market B deficit quota. | |
| 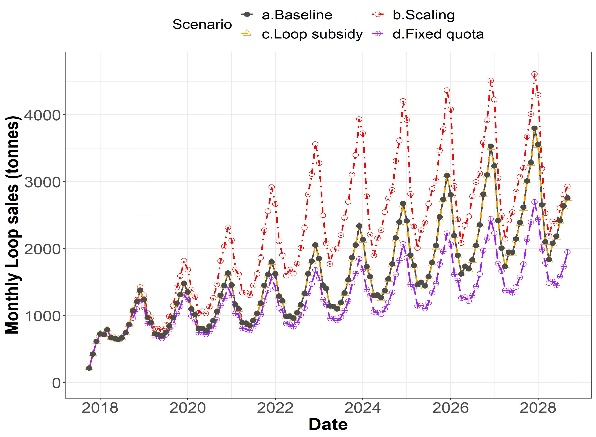 | 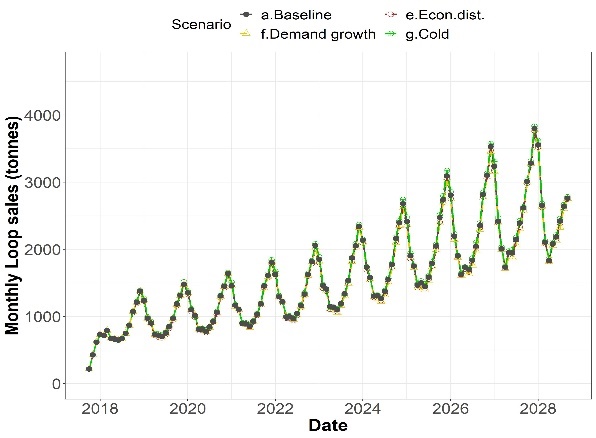 |
| 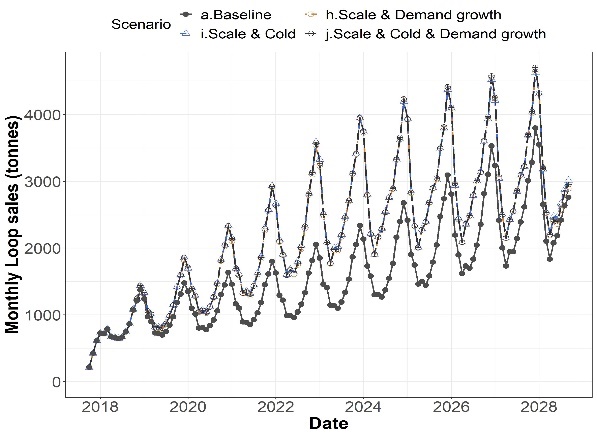 | 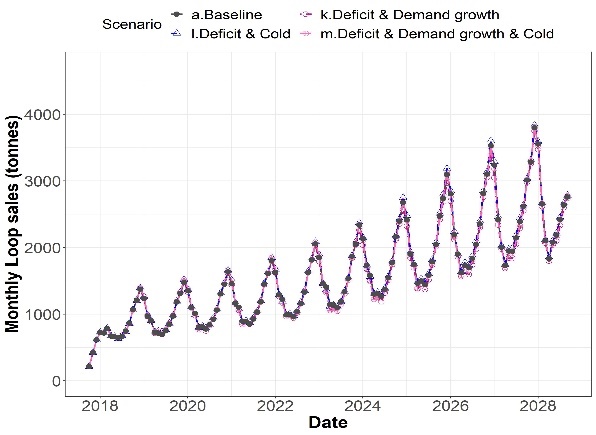 |
| Figure S4: Time-series of total Loop sales under the four scenario groups; (top left) Internal, (top right) external, (bottom left) Combination scale, (bottom right) combination Market B deficit quota. | |
| 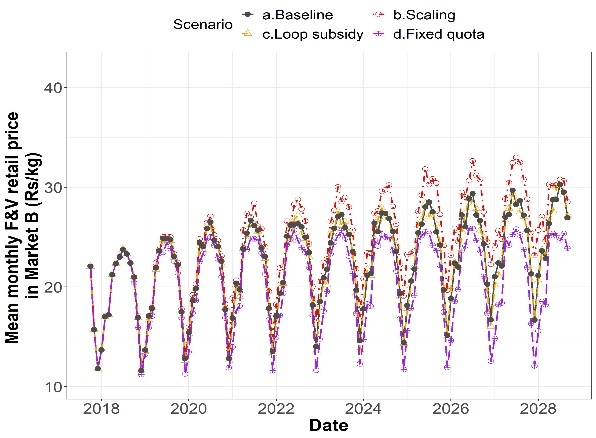 | 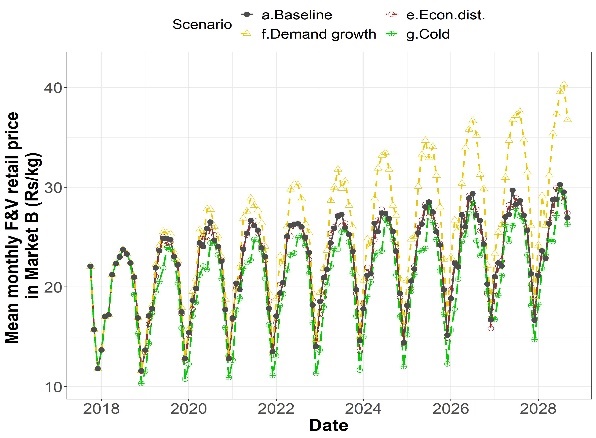 |
| 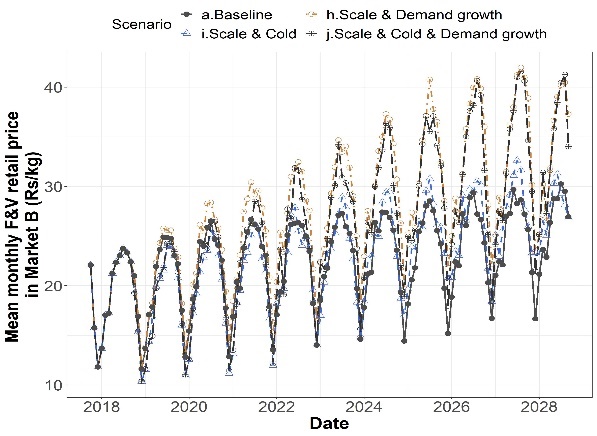 | 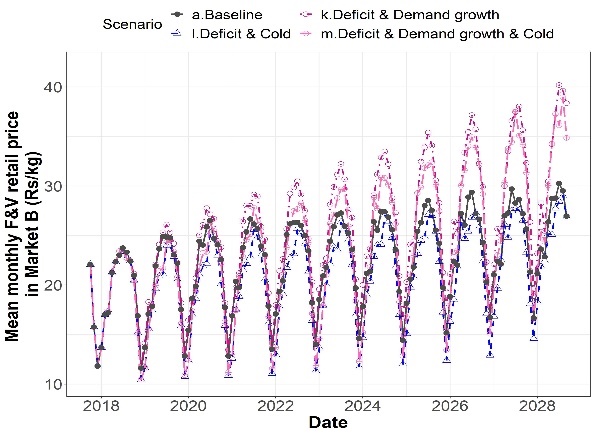 |
| Figure S5: Time-series of the monthly average F&V retail price in Market B under the four scenario groups; (top left) Internal, (top right) external, (bottom left) Combination scale, (bottom right) combination Market B deficit quota. | |
| 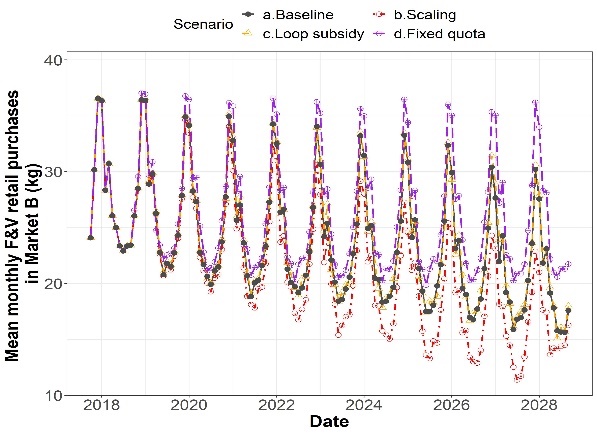 | 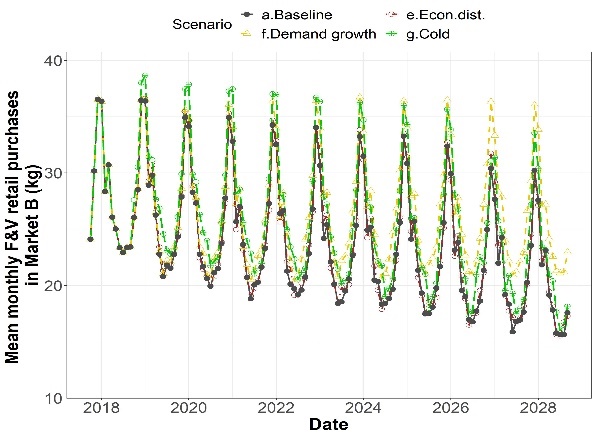 |
| 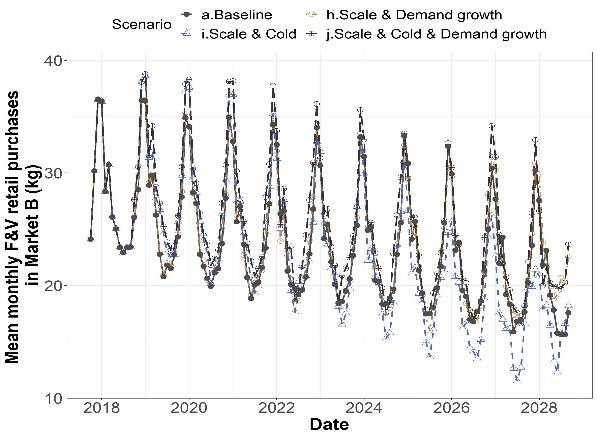 | 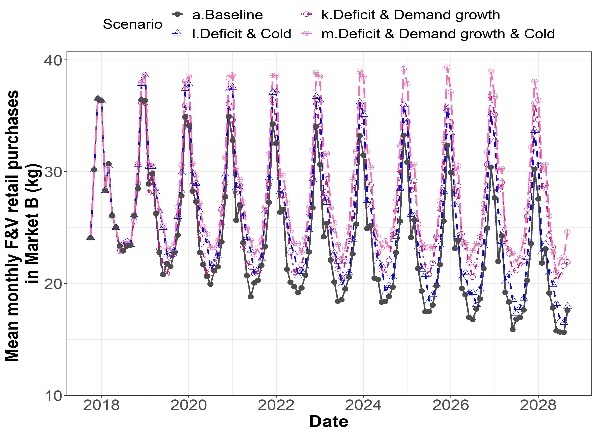 |
| Figure S6: Time-series of monthly F&V purchases per retail consumer in Market B under four scenario groups; (top left) Internal, (top right) external, (bottom left) Combination scale, (bottom right) combination Market B deficit quota. | |

| 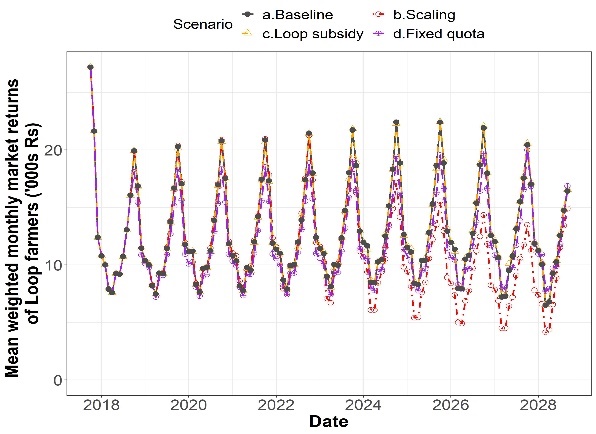 | 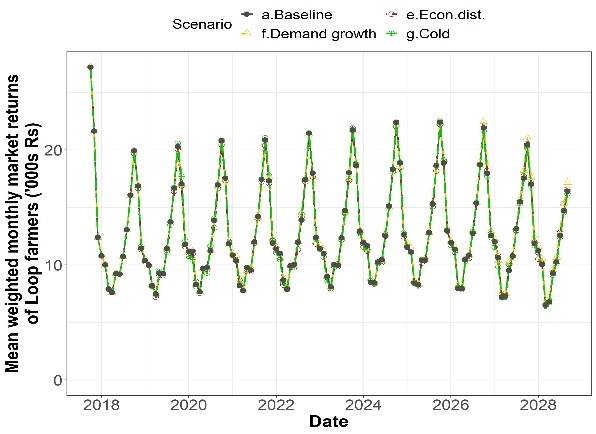 |
| --- | --- |
| 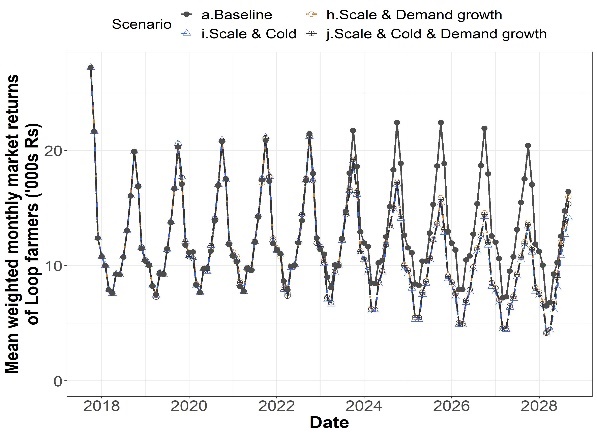 | 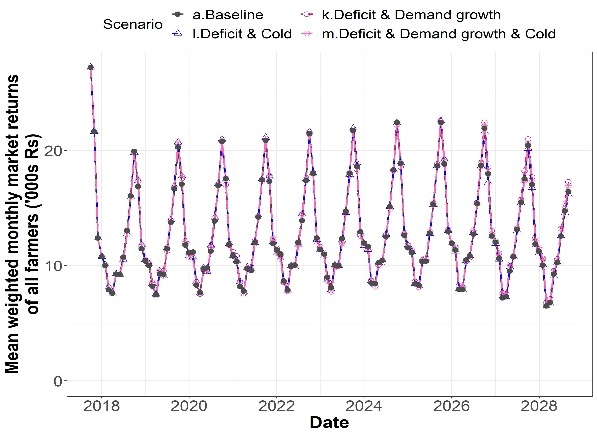 |
| Figure S7: Time-series of the monthly market returns generated by Loop farmers under the four scenario groups; (top left) Internal, (top right) external, (bottom left) Combination scale, (bottom right) combination Market B deficit quota. | |
| 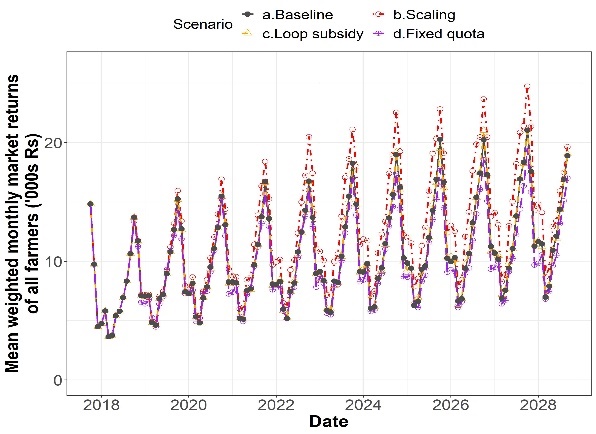 | 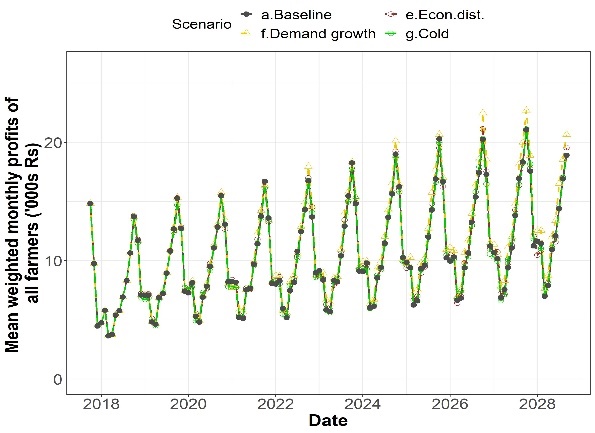 |
| 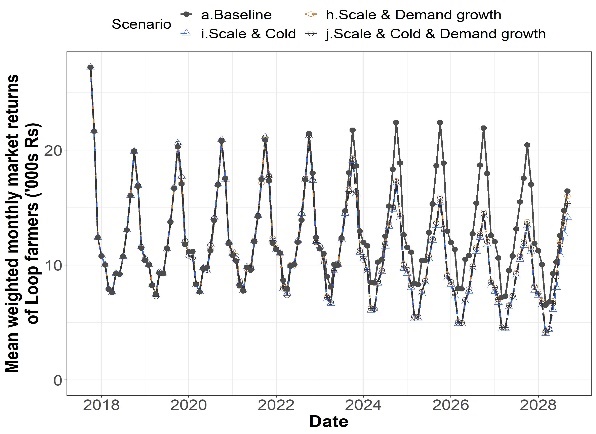 | 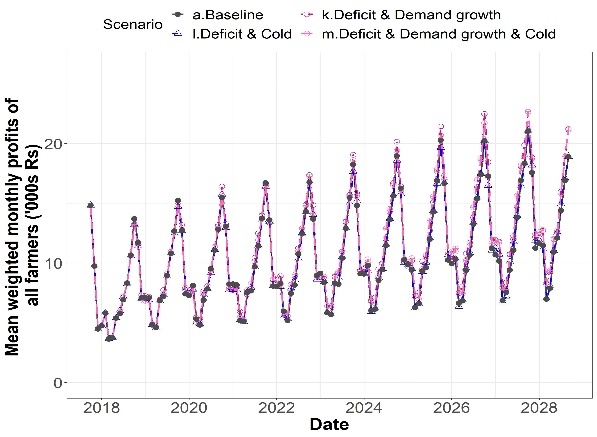 |
| Figure S8: Time-series of the monthly market returns generated by all farmers under the four scenario groups; (top left) Internal, (top right) external, (bottom left) Combination scale, (bottom right) combination Market B deficit quota. | |

| 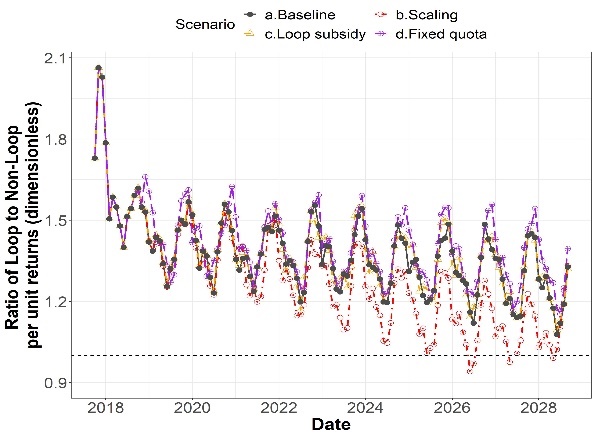 | 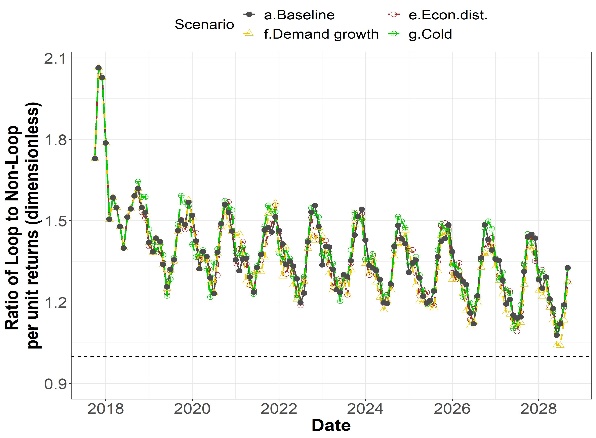 |
| --- | --- |
| 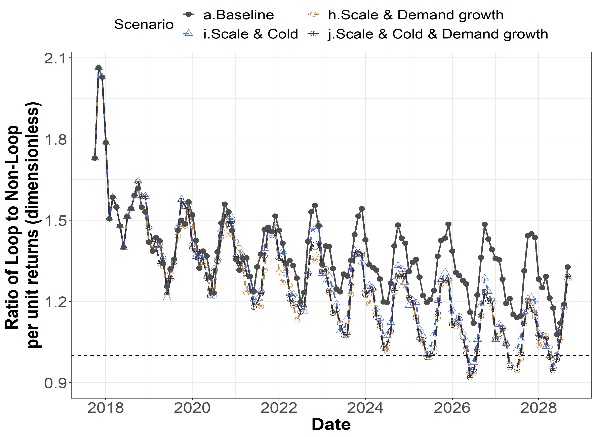 | 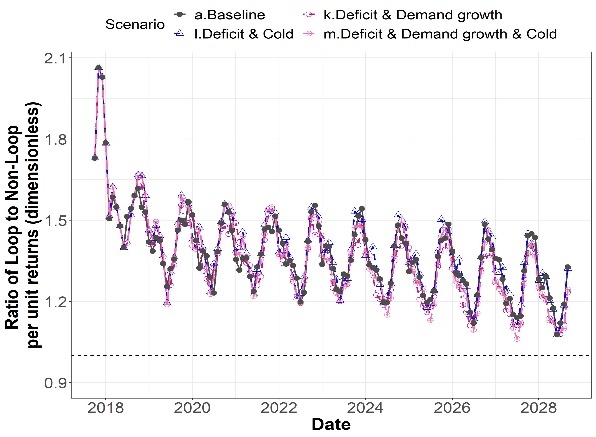 |
| Figure S9: Time-series of the relative Loop per unit profit under the four scenario groups; (top left) Internal, (top right) external, (bottom left) Combination scale, (bottom right) combination Market B deficit quota. The dashed line equals unity (i.e. Loop and non-Loop per unit profits are equal). | |
| 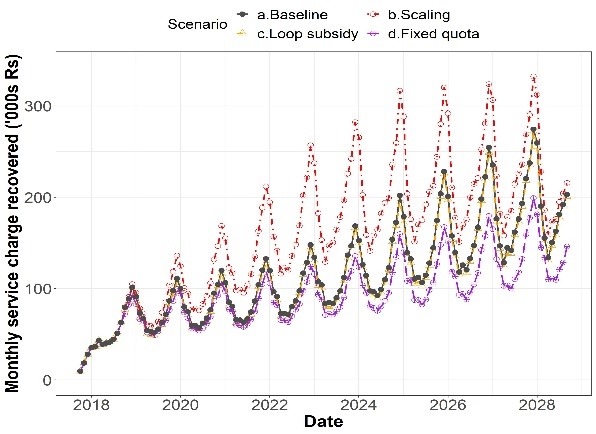 | 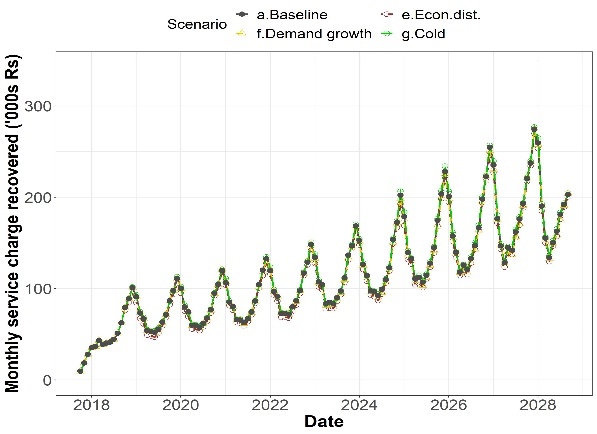 |
| 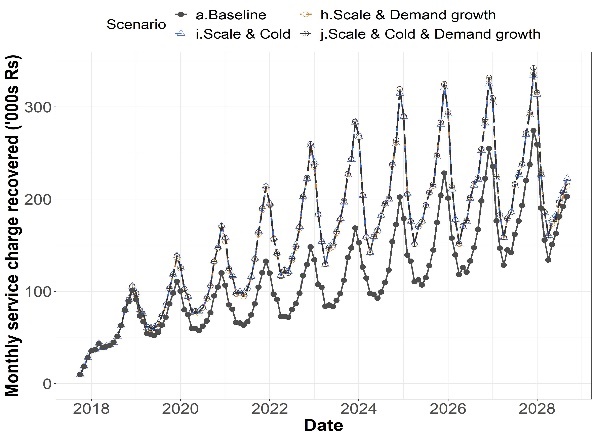 | 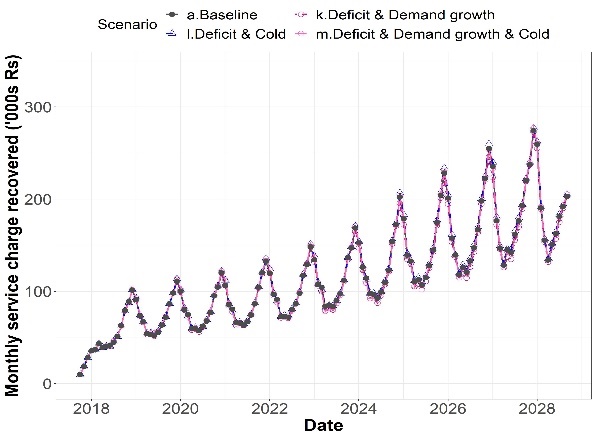 |
| Figure S10: Time-series of monthly aggregation service charges recovered under the four scenario groups; (top left) Internal, (top right) external, (bottom left) Combination scale, (bottom right) combination Market B deficit quota. | |

Appendix E: Model archive

The model simulated in the manuscript is a system dynamics model (SDM) developed around the functions of the ‘Loop’ aggregation scheme (DG, 2017) and the wider F&V value chain of the state of Bihar, India (Cooper et al., 2021). Here we provide further details around the underlying characteristics, structures and functions of the model, as to aid the transparency and replicability of the modelling process. The overarching aim of the model is: *To explore future scenarios that would evolve aggregation schemes and their wider enabling environments to improve the availability and affordability of fruits and vegetables (F&V) in the types of local markets that often serve nutritionally vulnerable consumers in Bihar, India*.

It should be emphasised that whilst the underlying structure and datasets of the model derive from Bhojpur and Muzaffarpur districts in Bihar, the model is not designed to explicitly forecast the medium to long-term future of the Loop scheme. Therefore, we acknowledge that this model is fundamentally an abstraction of reality, meaning certain decisions were made during model design to keep the model as generalisable and tractable as possible (e.g. reducing the influence of stochastic elements in decisions around which market to supply). We first provide an overview of the model’s characteristics, including the temporal horizon, resolution and spatial associations of the model. The individual modules and their associated submodules are then described. Moreover, the full version of the model and its associated equations can be obtained from the corresponding author on request.

E.1 Model overview

The model consists of five modules covering a range of F&V value chain processes and outcomes. The spatiotemporal characteristics of the model are as follows:

**- Temporal resolution:** one timestep represents one half-day (i.e. morning and afternoon)

**- Full model horizon:** 8036 half-days across 132 months – based upon the period from October 1^st^, 2017 to September 30^th^, 2028.

**- Parameterisation period:** Model timesteps 1-308 – corresponding to data within the Loop dashboard dataset from October 1^st^, 2017 to February 28^th^, 2018.

**- Validation period:** Model timesteps 309-668 – corresponding to data within the Loop dashboard dataset from March 1^st^, 2018 to August 31^st^, 2018.

**- Scenario period:** Model timesteps 669-8036 – corresponding to September 1^st^, 2018 to September 30^th^, 2028.

**- Spatial associations:** System dynamics models do not traditionally capture space explicitly (Neuwirth et al., 2016). Instead, the values and connections between the stocks, flows and feedbacks are associated with a particular locality. Here, the model is based upon the numbers and types of farmers, markets and traders found in Koilwar block in Bhojpur

district, Bihar. For instance, farmers have to decide between supplying the large wholesale-type market environment (i.e. the twin markets of Kayamnagar and Arra, Bhojpur district), or a smaller cluster of more retail-oriented markets. However, whilst the flows of F&V, finance and information across the model are implicitly associated within Koilwar, the underlying value chain structures, feedbacks and decision-making processes are more broadly representative of the archetypical F&V marketing systems commonplace across north India and South Asia (Maestre et al., 2017; Reardon et al., 2019).

The total stock of farming households (‘farmers’ – representing the head farmer in each household) in the model is split between (a) ‘Loop farmers’ that have signed-up to participate in aggregation, and (b) ‘non-Loop farmers’ that rely on self-supplying the market. F&V supplies flow downstream from the farmgate via a chain of decisions and feedbacks:

**- Whether to join the aggregation scheme?** Non-Loop farmers may decide whether to become ‘Loop farmers’ depending on (i) the expected profitability of Loop marketing pathways relative to non-Loop pathways, and (ii) expectations around the relative ability of the aggregation scheme to sell all of the aggregation at the market (i.e. the ability to guarantee sales and minimise wastage) (Module A).

**- Whether to aggregate on any given half-day?** Loop farmers supplying the market on any given half-day must choose between participating in aggregation or self-supplying the market as a temporary non-Loop farmer. This decision is based on the short-term performance of Loop relative to non-Loop, expressed as the relative per unit market profit of each pathway over the last week (i.e. 14 half-days) (Module B).

**- Which market to supply?** Loop aggregators aggregate and transport F&V of all quality into a single aggregation load per village. Therefore, aggregating farmers from each village must choose between supplying the larger, urban-based ‘Market A’ or the cluster of smaller local markets (‘Market B’) (Module C).

**- Which trader to supply at the market?** In turn, once at the market, aggregators and farmers must decide which of the trader-types to supply. In essence, both of the market and trader decisions are determined by: (i) the market returns they would expect to generate from each market/trader, accounting for the recent trends in prices and transport costs, and (ii) in order to minimise wastage, the expected ability of each market/trader to guarantee sales – relating to the capacity of each market/trader on any given half-day (Module C).

**- How much F&V to purchase for consumption?** Representing the model’s furthest points downstream are the stocks of F&V each consumer demands to purchase from retailers in Market A and Market B. The demand for F&V is dependent on the availability and retail prices of F&V in Markets A and B on any given half-day (Module D)

**- Farmer revenues and costs.** Loop and non-Loop farmers generate revenues and costs depending on the different volumes sold to each market/trader and the price dynamics at the time of sale. Farmers may then invest their profits into additional land for F&V cultivation and inputs for enhanced F&V yields (Module E).

SDMs take a more aggregated approach to individual decisions. For instance, rather than calculating the preference to supply Market A over Market B for every farmer individually, this model calculates the proportion of Loop farmers that opt to supply Market A over Market B. As detailed below, variables that express preferences are parameterised as stocks which can vary between 0 and 1; i.e. where 1 means that 100% of farmers opt for option A (e.g. Market A), and 0 means that 100% of farmers opt for option B (e.g. Market B).

E.2 Module A: Farmer population

Module A simulates the size of the F&V farmer population in Koilwar block, the respective number of Loop and non-Loop farmers, the processes of Loop adoption and dis-adoption, and the number of farmers in each sub-population who have marketable F&V produce available on any given half-day. In turn, the module then simulates the quantity of home F&V produce that is consumed within the farming household (i.e. removed from the Loop and non-Loop marketable stocks in Module B).

Farmer population

The *total farming population* represents the number of farming households in the model, which are then split between Loop and non-Loop households. The latest data point for the number of ‘cultivators’ (i.e. owners or co-owners of farms) in Koilwar block comes from the latest Census of India (2011). The initial number of farmers for October 2017 (12,087) was calculated by increasing the 2011 value by an annual population growth rate of 2.3%, which

| 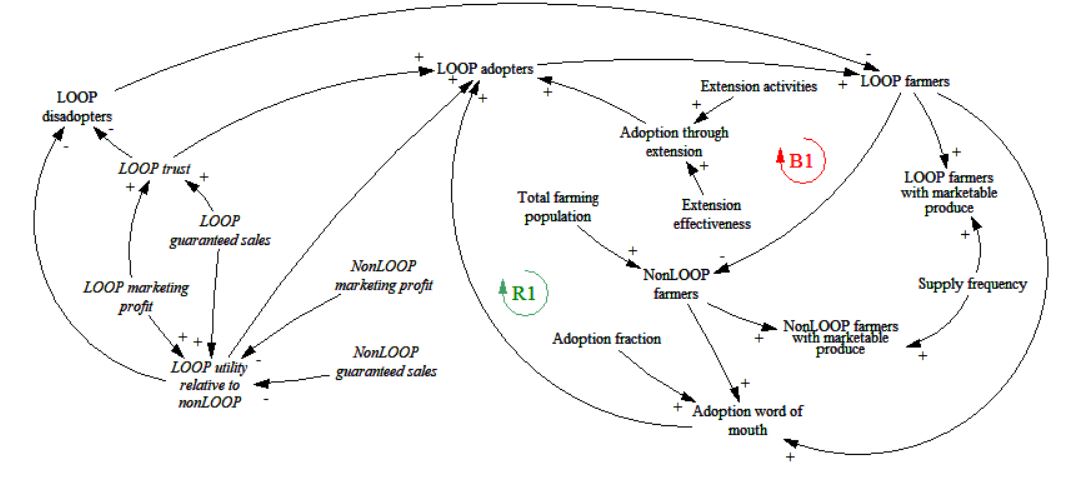 |
| --- |
| Figure S11: Causal-loop diagram (CLD) describing the dynamics driving Loop adoption and dis-adoption. Positive (+) polarities signify a positive relationship between cause and effect (i.e. when *‘*Loop adopters’ increase, so too does the number of ‘Loop farmers’). Negative (-) polarities denote inverse relationships between cause and effect (i.e. when Loop dis-adopters increase, the number of ‘Loop farmers’ decrease). Feedbacks: R1 = reinforcing feedback driving Loop adoption via word of mouth; B1 = balancing feedback limiting adoption via word of mouth as the number of non-Loop farmers reduces. Italicised variables are imported from elsewhere in the model. |

was also obtained from the 2011 Indian census for Koilwar block. In turn, as per Loop dashboard data for October 2017, the starting number of farmers participating in aggregation equals 90 (i.e. 0.7% of the model population).

The adoption of Loop practices in the model is based upon the Bass (1969) diffusion model, which simulates how new products are adopted amongst a population of users and

non-users. Here, Loop farmers are farmers which have signed up to the aggregation scheme (i.e. transactions recorded in the Loop dashboard), and therefore may choose to aggregate F&V produce rather than self-supply the market by their own means (and become subject to non-Loop transport costs). Every non-Loop farmer is assumed to have equal opportunities to adopt Loop practices.

The strength of aggregation adoption is a function of two base processes and two related modifications to the original Bass (1969) model (Figure S11).

**- Active aggregation scaling:** Captures the effects of active extension efforts, such as Digital Green’s community meetings, which actively seek to scale-up aggregation by encouraging non-Loop farmers to join Loop.

**- Adoption via word-of-mouth**: The second base process simulates the social interactions between Loop and non-Loop farmers, which help to spread the benefits of aggregation via word-of-mouth. The model is parameterised with household survey data, finding that Loop farmers tend to have social interactions with two non-Loop farmers per half-day.

**- Trust in aggregation:** We make two further extensions to the Bass (1969) model to reflect how adoption and dis-adoption are related to the performance of aggregation. First, the rate of adoption is also a function of the extent to which farmers *trust* the aggregation system to continue providing financial benefits over time and continue to sell all of the aggregated produce to traders at the market. As described in more detail below (Module E), trust is modelled as a self-evaluating process, where the marketing returns and the sales guarantee of the current month are compared to those of the previous month. Therefore, trust in Loop will weaken if returns (or guaranteed sales) show a decreasing trend, leading to fewer new adoptions and more dis-adoptions.

**- The utility of aggregation:** Inspired by a model of the adoption of pigeon-pea practices in Malawi (Grabowski et al., 2019), the adoption of aggregation practices here is also related to the expected returns and guaranteed sales of Loop *relative* to non-Loop. Essentially, aggregation adoption strengthens as the ratio of Loop to non-Loop returns and guaranteed sales grows.

The modelled trend for the total number of Loop farmers between March – August 2018 is evaluated against the observed data in Appendix B.

Farmers with produce available to market

The production of F&V is modelled as a three-step process: (i) the number of Loop and non-Loop farmers that cultivate their F&V production on any given half-day, (ii) the seasonal F&V yield, and (iii) the area of productive land under F&V cultivation.

Point (i) above is based on data collected in the household surveys, where we asked farmers about the number of days in each season over the reference period^[[1]](#footnote-1)^ that they marketed F&V production. During the Rabi season, each Loop and non-Loop farmer have F&V ready to market once every 10 half-days (5 days); during the rest of the year, both subpopulations have F&V ready to market once every 18 half days. The farmers with marketable produce on any given half-day are then exported into Module B to calculate the marketable produce available.

Consumption of home produce

The final part of Module A generates the volume of F&V that is consumed before leaving the farmgate (Figure S12). First, the baseline household consumption rates for Loop and non-Loop farmers in Bhojpur district are calculated from the household surveys. The baseline consumption rates are converted from annual totals to half-day rates (mean average Loop household consumption = 380 grams/household/day; mean average non-Loop household consumption = 330 grams/household/day).

From here, the respective base consumption rates are added to when the farming populations feel more secure in consuming their produce at home (rather than selling it to market), based on the assumption that increased market volatility and uncertainty encourages smallholders to consume more of their own produce for food security purposes (Fafchamps, 1992). As such, any time (*t*) the average retail demand for F&V (mean average of Markets A and B) drops below the initial parameterised value (see Module D), then farming households are triggered to consume an additional amount from their production. This logic may be expressed via the following equations:

$${Consumption of own produce}_{i,t}={Baseline consumption of own produce}_{i,t}\times{(1+ABS(SMOOTH(Additional consumption}_{t}, {Supply frequency}_{i,t} )$$

$${Additional consumption}_{t}=\frac{({Average retail demand}_{t}-Initial retail demand)}{Initial retail demand}$$

Where *ABS* equals ‘absolute value’, *SMOOTH* equals the STELLA function ‘smooth’ (generates a moving average) and *i* equals the household type (i.e. Loop or non-Loop).

If the average retail demand at any given point in time is greater than the initial demand, then the additional consumption equals zero (i.e. farming households do not

| 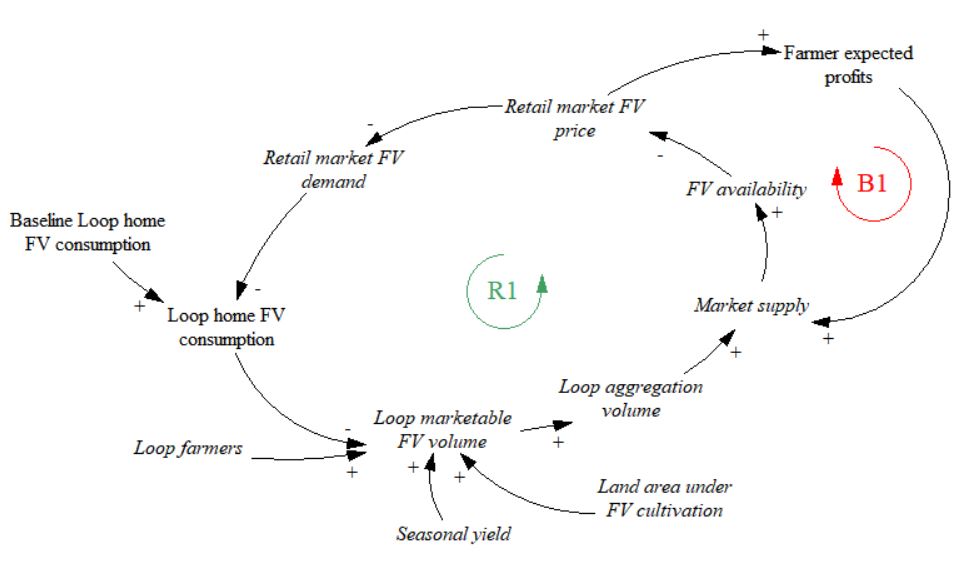 |
| --- |
| Figure S12: Reinforcing feedback (R1) linking Loop market supplies, market prices and the consumption of own produce. However, this reinforcing feedback is counterbalanced by a balancing feedback (B1), whereby as prices reduce, so too do the expected returns of supplying farmers (B1), meaning farmers are likely to divert their supplies towards more profitable traders (e.g. local wholesalers or distance traders). |

consume more than they need from their own production). Moreover, in order to introduce an element of perceived security, the additional consumption is smoothed by the market supply frequency (i.e. farmers are only aware of the going rate when they visit the market). The volumes of home produce consumed by Loop and non-Loop households are then subtracted from their respective marketable production stocks (Module B).

E.3 Module B: Production & aggregation

Module B simulates the volumes of F&V produced and made available on any given day, plus the associated F&V flows downstream towards Markets A and B (Figure S13).

F&V yields

It is important to acknowledge that this SDM is not a specialised crop model; as a consequence, the data demands and model structures needed to disaggregate and code individual crop growth rates, irrigation requirements and fertiliser demands are not captured here. Therefore, this model makes a number of generalisations to estimate the overall flows of F&V to markets in the study area,^[[2]](#footnote-2)^ whilst retaining key elements of reality such as seasonality and wastage rates.

F&V yields have been calculated through a combination of the Loop dashboard dataset and the National Horticultural Production Database 2014-2015 (NHB, 2015).^[[3]](#footnote-3)^ First,

| 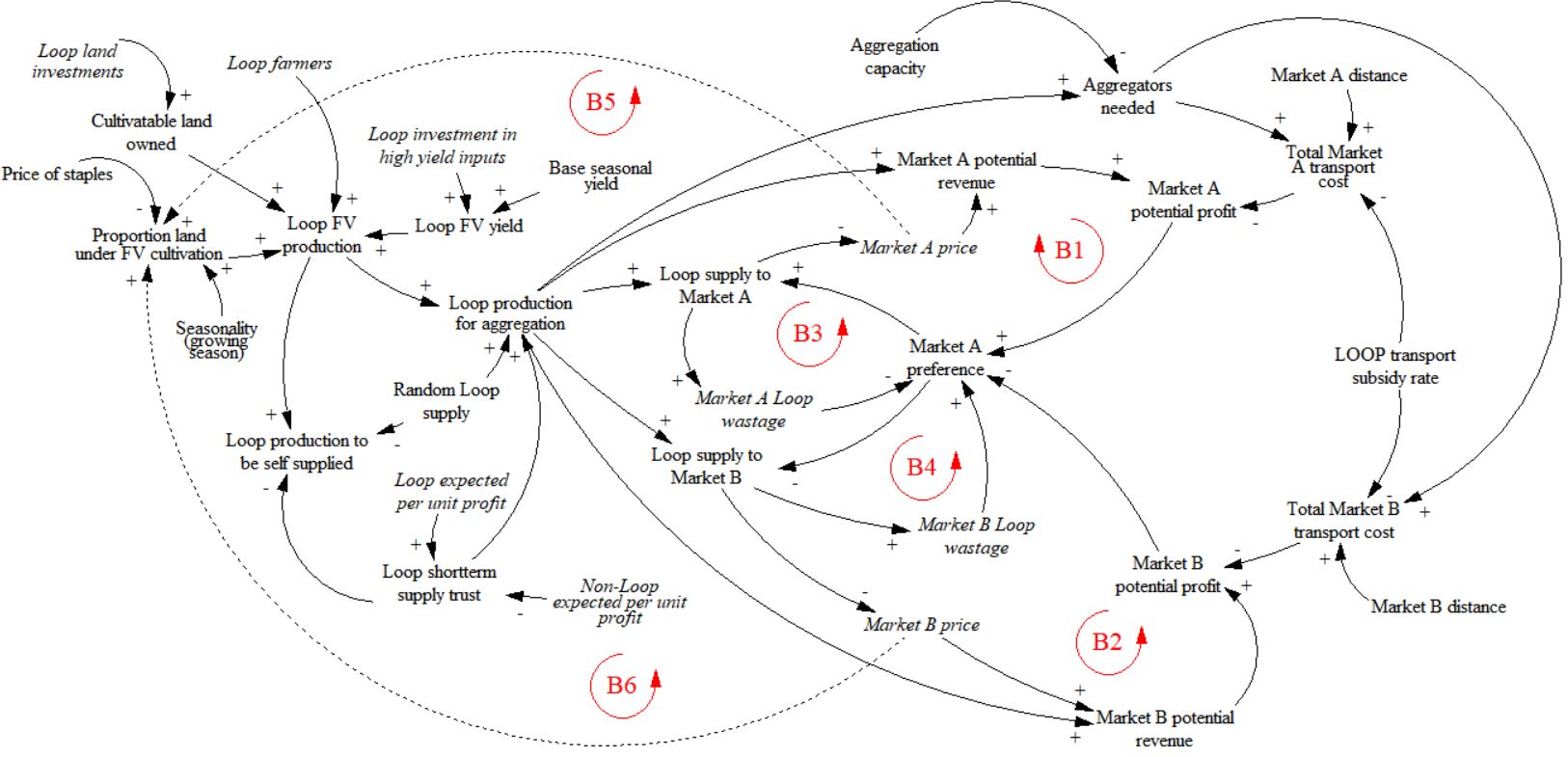 |
| --- |
| Figure S13: CLD summarising the drivers of Loop F&V production, market supplies and feedbacks with decisions regarding F&V land allocation and market choice. Dashed lines are for visual clarity where multiple causal arrows overlap. Feedback key: B1 – balancing feedback where increasing supplies to Market A may reduce market prices and expected returns, producing a decrease in Market A preference; B2 – the equivalent of B1 for Market B; B3 – balancing feedback where increasing supplies towards Market A may lead to increase wastage, producing a decrease in Market A preference; B4 – the equivalent of B3 for Market B; B5 – an increase in supplies to Market A may reduce prices below the price of staples, leading to farmers preferring staple production; B6 – the equivalent of B5 for Market B. |

the top three aggregated crops in each season were subset from the Loop dashboard. During the Rabi 2017-2018, brinjal, cauliflower and cabbage accounted for 69.1% of all F&V aggregated from the study site. In turn, cauliflower, tomato and bottle gourd made up 45.6% of all aggregation quantities during Zaid 2018. Lastly, bottle gourd, bitter gourd and sponge gourd accounted for 72.5% of all F&V aggregated during Kharif 2018. From here, seasonal weighted averages were calculated from the crop-wise yields obtained from NHB (2015) and NIFTEM (2013). It is possible that the yields resulting from the weighted averages of the top three crops may be different than if all aggregated crops were included. However, yield and production data for some of the less popular crops (e.g. Ivy gourd and Jhiguni) were not reliably available, hence the restriction to the top three crops in each season.

The seasonal yields are coded into the model as a ‘graphical function’, which provides a given output for a given input by means of an x-y graph (Table S6). Here, the input is model time (i.e. half-day 1, half-day 2… half-day 8036), meaning a F&V yield is produced for all of the model’s time-steps. Loop and non-Loop farmers are parameterised to have the same baseline yields. However, as described more in Module E, Loop and non-Loop farmers are able to invest their profits into inputs for enhanced yields over time.

Land area under F&V cultivation

Two main factors drive the area under F&V cultivation: (i) total land area under cultivation, (ii) the seasonal proportion of land area dedicated to F&V.

| 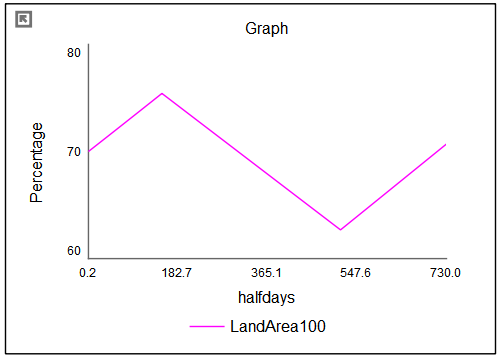 | Figure S14: The seasonal trend over the first year of simulation for the proportion of cultivatable land under F&V cultivation. A total of 730 half-days make up the first year of simulation. |
| --- | --- |

The total cultivatable land areas for each farmer subpopulation were informed by our household survey data. To avoid biasing favourable production outcomes towards Loop

farmers at the start of the simulation, both sets of farmer subpopulations are parameterised with 49 katha.^[[4]](#footnote-4)^ In turn, Loop and non-Loop farmers may then opt to purchase additional land for cultivation, depending on the evolution of their cumulative profits over time (Module E).

During the SGMB sessions it became apparent that the proportion of land under F&V cultivation changes seasonally. In general, farmers tend to dedicate more of their land to F&V during the Rabi growing season, and favour stable crops and pulses during the rainy season. In order to capture these seasonal cycles and transitions, as opposed to unrealistically abrupt switches at the start of each new season, land area under F&V

cultivation is modelled as a stock, with an inflow that actively increases the land area, and an outflow that reduces the land area. Therefore, we assume that land area under F&V cultivation peaks in the middle of the Rabi season (mid-December each year) and falls to a minimum in the middle of the combined Zaid and Kharif season (i.e. mid-June each year).

From here, the proportion of land under F&V cultivation may then further increase or decrease depending on the price of F&V relative to the price of staples. Due to the lack of regional, monthly resolution data, we parameterise the price of staples based on a time-series of rice, wheat, and maize prices across India between 2017-2018 (USDA, 2019). Essentially, this monthly time-series is repeated for the length of each simulation, with the inflow that increases the proportion of land under F&V cultivation strengthening when the price of F&V exceeds the price of staples. The opposite then occurs when the price of staples exceeds the price of F&V. The F&V prices refer to the mean weighted per unit market prices received by Loop and non-Loop farmers, separately (Module E).

The resulting time-series for non-Loop farmers over the course of the first year can be seen in Figure S14, with the cultivatable land area under F&V cultivation peaking at ~75% during the Rabi and falling to ~62% by the mid-Kharif.

F&V quality

From the SGMB and value chain discussion, low-quality produce (e.g. often visibly damaged and/or less fresh) usually fetches a lower price at the market. The SGMB discussions uncovered a variety of pricing models, with some traders offering to buy low-quality F&V at half the price of high-grade produce, and others pricing low-quality produce 5-10 Rs/kg less than high-grade produce.

According to the SGMBs, Loop aggregations average 80% high-grade produce. However, neither farmers or aggregators grade produce at the farmgate, meaning the quality is only determined by the commission agents once the produce arrives at the market. As a consequence, under the present aggregation scheme, both high- and low-quality produce is sent to the market together (i.e. unsorted and ungraded). Therefore, the model treats F&V quality as an external process by randomly generating values which represent the proportion of high-quality F&V in Loop and non-Loop market supplies on any given half-day. The model randomly samples between minimum and maximum percentages of high-quality produce, which are set at 60% and 100%, respectively. The values are then smoothed by 14 half-days, so that the quality dynamics operate at the weekly timescale.

The proportions of high-quality supplies are then exported to the Market Supply module (Module C) to influence the prices traders offer Loop and non-Loop produce (in the model, traders pay 50% of the full price for low-quality produce). For example, say that a Loop aggregation (1000 kg) to a distance trader in Market A on any given half-day consists of 80% high-quality produce and 20% low-quality produce. The trader is offering 10 Rs/kg for high-grade produce, and 5 Rs/kg for low-grade produce. The total Loop revenue from sale would therefore equal: $\left( 0.8\times1000\times10 \right)+\left( 0.2\times1000\times10\times0.5 \right)=9000 Rs$.

Short-term aggregation satisfaction

Loop farmers do not always opt to participate in aggregation. Deciding not to aggregate is not necessarily dis-adoption; instead, farmers may find greater convenience in self-supplying the market if they also have other work to do at the market.

Therefore, the proportion of Loop farmers with marketable produce supplying the aggregation scheme is a function of two processes. The main driver is the short-term satisfaction in the per unit returns generated from aggregation relative to the equivalent outcome for non-Loop pathways (Figure S13). Therefore, satisfaction in aggregation (and the proportion of Loop farmers willing to aggregate) changes proportional to the percentage difference between the per unit returns of Loop and non-Loop pathways. In turn, aggregation satisfaction is smoothed over 14 half-days, reflecting the importance of the weekly scale in marketing decisions (e.g. farmers tend to supply between once and twice per week).

Second, randomness is introduced to capture sporadic supply decisions. During the SGMBs, participants felt that 5% of Loop farmers may self-supply the market on any given day. Therefore, the *Loop random self-supply* variable generates a random number between 0 and 0.05 (taken the conservative estimate to minimise model stochasticity and improve output tractability), which is then subtracted from the short-term satisfaction in aggregation to generate the actual proportion of Loop farmers participating on any given half-day.

On-farm costs

The hired on-farm costs for Loop and non-Loop farmers are parameterised from household survey data. The on-farm costs are split into two categories. The first category contains seven F&V input costs: (i) seeds, (ii) fertilisers, (iii) pesticides, (iv) irrigation, (v) manure, (vi) tractor and (vii) rotavator. The second category captures the cost of hired labour across seven on-farm activities, namely: (i) ploughing, (ii) planting, (iii) hilling, (iv) weeding, (v) watering, (vi) harvesting and (vii) others.

In the surveys, each farmer had to answer questions about the above 14 costs for each of their F&V crops grown over the reference period. For example, if a farmer had grown four F&V crops over the reference period, then data on 56 individual costs (i.e. 14 x 4) were obtained. Outliers for each cost category were removed using the ‘1.5*interquartile range’ (IQR) technique, before density plots were graphed for each activity to understand the shape of the distribution (i.e. uniform, normal, log-normal). These distributions were then coded into STELLA using random distribution functions, allowing the model to generate annual costs. From here, the model converts to the half-daily scale by dividing the randomly generated costs for each activity by the number of half-days per year (i.e. 730).

The same strategy is used to parameterise labour costs. Survey participants were asked about the number of hired wage labour ‘man-days’ hired for each activity over the reference period. For each activity, outliers were first removed, before distributions were plotted from the mean averages and standard deviations were generated. Next, the financial costs of wage labour were generated by multiplying the man-days by the minimum daily wage labour salary for unskilled farm labourers (257 Rs/day). As per the input costs, the annual labour costs are divided by 730 half-days to match the temporal resolution of the model. The respective Loop and non-Loop on-farm costs are then exported to Module E to calculate the net F&V revenues of each farmer subpopulation.

It would also be possible to extend the model by including the implicit costs of owned productive assets and family labour. To assess the effects of including these costs on the study results, we calculate the per household imputed costs of family labour, as well as owned (i) seeds, (ii) manure, (iii) irrigation, (iv) animal traction, (v) thresher/harvester and (vi) rotavators, as per the guidelines of FAO (2016): i.e. the average rented price was multiplied by the quantity of each owned asset used, as per our survey data (e.g. kilograms of owned manure and grams of crop seeds). These calculations provided 14 different distributions: the imputed costs of six productive assets for Loop and non-Loop farmers, plus Loop and non-Loop family labour. However, once outliers were removed for each group using the ‘1.5*interquartile range’ (IQR) approach (as per the hired costs), Loop and non-Loop farmers on average were found to only incur imputed costs for owned manure and family labour. On average, Loop farmers were found to incur imputed costs of 210 Rs/year and 9100 Rs/year on owned manure and family labour, whilst non-Loop farmers incurred imputed costs of 862 Rs/year and 9000 Rs/year, respectively. We find that adding these costs to the model produces negligible impacts on the results of the study, with the 72 outcome ratios (6 outcomes across 12 scenarios) presented in Section 2.2 and Section 2.3 of the main manuscript changing by a mean absolute percentage error of 0.837%. Furthermore, the inclusion of imputed costs does not cause any of the 72 outcome ratios to change their significance level from those depicted in Figure 6 and Figure 7. Therefore, given that imputed costs are not widespread, the results presented in the main manuscript include explicit hired expenditures only.

Loop and non-Loop market preference

Given that market conditions such as demands, capacities and prices are dynamic, aggregators and farmers actively work together in reality to decide which market to supply. Therefore, it is important that the model captures the salient elements of this decision-making process in order to simulate scenarios that explore the ways in which aggregation schemes may increase the availability of F&V in local markets.

Market choice could be modelled as a binary choice, meaning all of the aggregation on any given morning would go to *either* the larger, urban-based Market A or the smaller, more local Market B. However, given that Loop aggregations from Koilwar block supplied more than one market on 40% of days between October 2017 and August 2018, a binary choice is deemed less suitable than modelling market preference as a stock to capture the proportions that are supplied to either market.

Informed by our SGMB discussions around the drivers of market choice, the stock representing the preference for Market A over time is driven by (i) the expected relative

|  | Figure S15: Graphical function relating the expected profitability of supplying Market B over Market A to the change in the proportion of non-Loop farmers supplying Market B on any day. The same graph form is used for ‘expected guaranteed sales’ and ‘effect of expected profits on trader capacities’ (Module C). |
| --- | --- |

returns of Market A over Market B, and (ii) the expected relative guaranteed sales of supplying Market A over Market B. The second driver essentially combines farmer concerns around market capacities, wastage and convenience, with farmers communicating the importance of Loop ‘guaranteeing sales’ via their links with larger markets and traders. In

practical terms, the module imports the proportion of Loop’s supply to Market A that was successfully sold at the market, and compares it to the equivalent figure for Market B.

Therefore, the inflow to the Market A preference stock is positive (with a magnitude proportional to the differences between Market A and Market B) when the net effect of relative returnability and guaranteed sales is positive, leading to an increase in the preference for Market A over B. In turn, the market preference is randomly varied by up to

±5% to reflect the small proportion of aggregators who would supply a different market due to having some other work in the market vicinity.

Four feedbacks emerge from this structure (Figure S13). First, two balancing feedbacks (B1 and B2) reflect how an increase in Loop supply towards Market A may have a negative impact on the prices offered by traders, leading to lower expected returns and a decline in the preference for Market A. Likewise, the remaining two feedbacks (B3 and B4) show how increasing aggregations towards Market A may increase wastage, for example, if supply outweighs demand. As a consequence, aggregators will begin to channel supplies away from Market A in favour of Market B. The preference for Market A over Market B is then connected to the dynamics of the Loop marketable stock to calculate the percentage of the aggregated volume that is sent towards Market A (see below).

The proportions of non-Loop supplies towards either market is similarly based on expected returns and guaranteed sales. A baseline 10% of non-Loop farmers supply Market B on any given day, with the additional percentage determined by the dynamics of the ‘desired capacity’ model of Sterman (2000, p.807). In essence, the expected profitability and guaranteed sales of Market B are compared to the equivalent figures for Market A over the past month, generating expected *relative* profitability and guaranteed sales. In turn, each of these relative measures form the inputs to two graphical functions (Figure S15), which generate an additional percentage of non-Loop farmers supplying Market B for given levels of relative profitability and guaranteed sales.

Marketable stocks

The main outputs here are the volumes of aggregated F&V that flow downstream towards the different types of traders in Market A and Market B (Figure S16): (i) distance traders in Market A, (ii) local wholesalers in Market A, (iii) local retailers serving F&V consumers in Market A, (iv) local wholesalers in Market B, and (v) local retailers

| 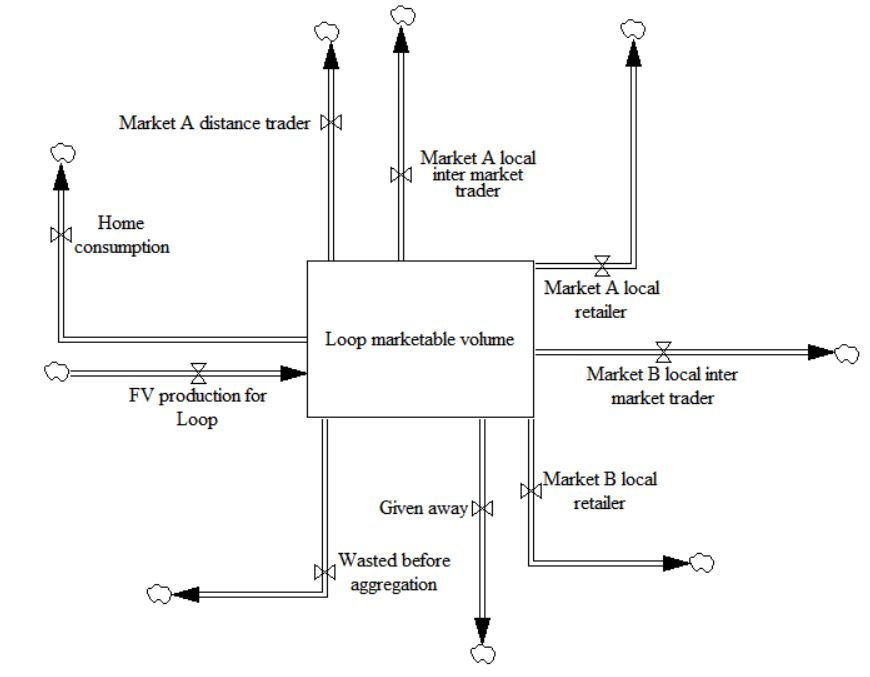 | Figure S16: The stock and flow structure underlying the Loop marketable F&V volume, depicting one inflow (Loop F&V production), five marketing outflows and three additional outflows. The eight outflows are modified by different converters (e.g. Loop Market A distance trader preference) that are described in text but have been excluded here for visual clarity. |
| --- | --- |

serving F&V consumers in Market B. These marketing outflows are moderated partly by the inflow of Loop and non-Loop F&V production, as well as the various feedbacks driving market (see above) and trader preferences (Module C). It is important to note that Loop aggregations only flow downstream towards the markets during the morning of each day, reflecting the overarching tendency for aggregators to arrive early at the market in order to supply the large distance traders which often leave the market by late-morning. In contrast, non-Loop farmers may supply in either the morning or the afternoon.

Three additional non-marketing outflows takeaway from the Loop marketable volumes, namely the volumes: (i) wasted before aggregation (i.e. on the farm or during loading), (ii) given away to family and friends, and (iii) eaten at home (Module A). The other two non-marketing outflows are parameterised from our household survey data, with the Loop farmers surveyed in Bhojpur district (n = 60) giving away and wasting 2.5% and 4.5% of their F&V production over the reference period, respectively.

The structure of the non-Loop marketable produce is essentially the same as in Figure S16. However, the non-Loop F&V stock is also fed by the F&V produced by Loop farmers but supplied along non-Loop pathways (i.e. produce not put forward for aggregation).

E.4 Module C: Marketing

Until this point, the model generates the number of farmers participating in aggregation, their production and supply quantities, and their preference for the large urban-type market over the local-type market. The inputs into Module C derive from the Loop and non-Loop marketable stocks (Module B), namely the quantities of F&V from Loop and non-Loop farmers to the different traders in each of the markets. The internal dynamics of this module then simulate the supply and demand schedules of each trader type, generating

price information, revenues, commissions and wastage rates. The outcomes of this module are the volumes of F&V sold at each market (and by each trader type), the marketing revenues and profits, and the impacts of retail prices on consumer demands (see Module D).

There are two key differences between Market A and Market B: (i) market structure and (ii) marketed quantities. Market A is based on an urban-type market which accommodates the demands of distance traders, wholesalers and local retailers. Distance

traders generally export F&V from large regional markets in Bhojpur to wholesale markets in cities like Patna, Ranchi and Kathmandu. Market B is more representative of smaller semi-rural markets, which cater for local wholesalers and local consumers. Therefore, Market A is

parameterised to have a peak capacity of ~150,000-200,000 kg/day during the peak Rabi season, whilst Market B is parameterised with a capacity of 15,000-20,000 kg/day.

External distance market

Both Market A and Market B are assumed to be price takers, meaning their prices are partly informed by prevailing external price trends. According to our SGMB sessions, distance traders in Market A have a significant market share, purchasing between 50-90% of all F&V arrivals in urban markets in Bhojpur (e.g. Kayamnagar wholesale market). In turn, the price distance traders offer to F&V sellers in Market A is informed by the price they will receive in the destination market. However, rather than modelling the internal dynamics of these large external markets, the model uses a time-series of prices from the National Horticultural Board’s ‘MIS Weekly Report’ dataset.^[[5]](#footnote-5)^ The dataset is aggregated to the monthly resolution; therefore, rather than assuming the price is constant throughout the month, the model assumes a continuous interpolation between adjacent months/data points (Table S6).

The external market price time-series was created by taking the monthly weighted average price of the four F&V crops that make up the majority (~55%) of all Loop aggregations across the study site October 2017 and September 2018: brinjal, cauliflower, bitter gourd and lady finger. For example, in October 2017, brinjal made up 59% of the four-crop subset quantity from the study site, whilst cauliflower, bottle gourd and lady finger represented 1%, 10% and 30%, respectively. In turn, according the NHB dataset, their respective prices in Patna were 42 Rs/kg, 45 Rs/kg, 40 Rs/kg and 36 Rs/kg, producing a weighted average price of 39.7 Rs/kg. This process was then repeated for the following 11 months (Table S6).

To arrive at the price offered to farmers in Market A, distance traders work at a margin that is half of the price in the distance market. For example, according to the SGMB

discussions, if distance traders expect to receive 22 Rs/kg at the distance market, they will often offer ~11 Rs/kg at their home market (plus or minus any local supply and demand effects). This figure is then exported into the Sterman (2000) price formation structure (see below) within the distance trader module.

Loop trader preference

As shown for the distance traders (Figure S17), the preference for a given trader is a function of their relative prices and ability to guarantee sales. Modelling a two-way choice, such as deciding between supplying Market A or Market B, is relatively straightforward, as the preference for the first option equals $x$ and the preference for the second option equals $1-x$. However, this becomes more complicated with three possible options (e.g. distance traders, local wholesalers and retailers). Therefore, to model trader preference as a stock (i.e. a non-binary variable with temporal dynamics), it is assumed that Loop aggregators first prefer to supply distance traders in Market A ahead of the other two traders. Therefore, if the distance trader preference equals ‘0.8’, the remaining 20% will run-off towards wholesalers and retailers. From here, the second stage begins, where local wholesalers and retailers compete for the remaining supply (Figure S17).

Next, the change in the preference for distance traders over time is proportional to (i) the distance trader’s price relative to the best price offered by the other traders, and (ii) the

| 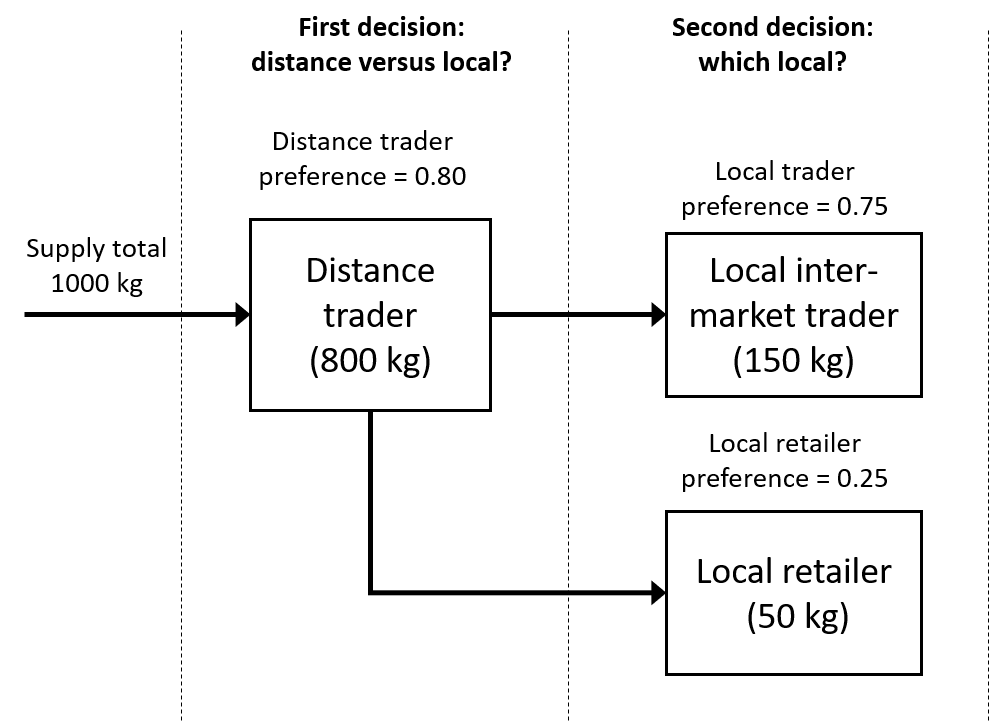 | Figure S17: Graphical representation of the logic driving the proportions of supplies to each of the three trader types in Market A. |
| --- | --- |

trust in the ability of the distance traders to guarantee sales. This latter factor reflects the perceptions expressed during the SGMB sessions that the ability of a trader to guarantee sales is critically important to develop trust. If the distance trader’s inventory is at capacity, then the trader will be able to purchase more F&V at a suitably profitable price. In this case, excess supply will flow towards local wholesalers, having a negative impact on the trust in the distance traders (see ‘Unsold supplies’ below). Rather than a direct comparison between trader types, the number of farmers who are unable to sell their produce to their desired trader on any given half-day is compared to the equivalent figure from one month ago (i.e. intra-trader evaluation).

Non-Loop trader preference

Whilst we assume that non-Loop farmers send relatively stable proportions of F&V to Markets A and B, the model allows non-Loop farmers to choose their trader type at the market within certain constraints.

The overarching structure is similar to that of Loop trader preferences; however, unlike the Loop calculation that only happens in the morning, the non-Loop calculation happens in both the morning and afternoon. Therefore, in the morning, the non-Loop trader preference works exactly the same as the Loop preference (i.e. a two-step process). However, in the afternoon, non-Loop farmers only have wholesalers and retailers to choose from (in both Market A and Market B). In this case, the proportion of non-Loop wholesale supply equals the local trader preference, whilst the remaining proportion goes to retailers.

The model also includes three constraints to ensure that each trader in Market A receives at least some produce. Therefore, the non-Loop preference for distance traders is constrained between 0.1-0.8 (ensuring that at least 10% of the non-Loop produce runs-off to the other traders).

Trader numbers and capacities

Market capacity on any given half-day is a function of: (i) the number of different types of traders in either market, (ii) the number of each trader type on any given half-day, and (iii) the capacity of F&V that each trader can handle per half-day. The latter two drivers are informed by a combination of SGMB data and our value chain surveys. We co-developed time-series with SGMB participants to estimate the numbers of each trader type in Market A over time – inputted into the model as graphical functions (Table S6). The trader numbers in small local markets were perceived to be relatively static; as such, a baseline of up to 5 local wholesalers and 50 retailers are parameterised to operate from Market B per half day.

Each trader type is also associated with a frequency; distance traders in Market A operate only in the morning (i.e. once every two half-days). Local traders as an aggregated

| 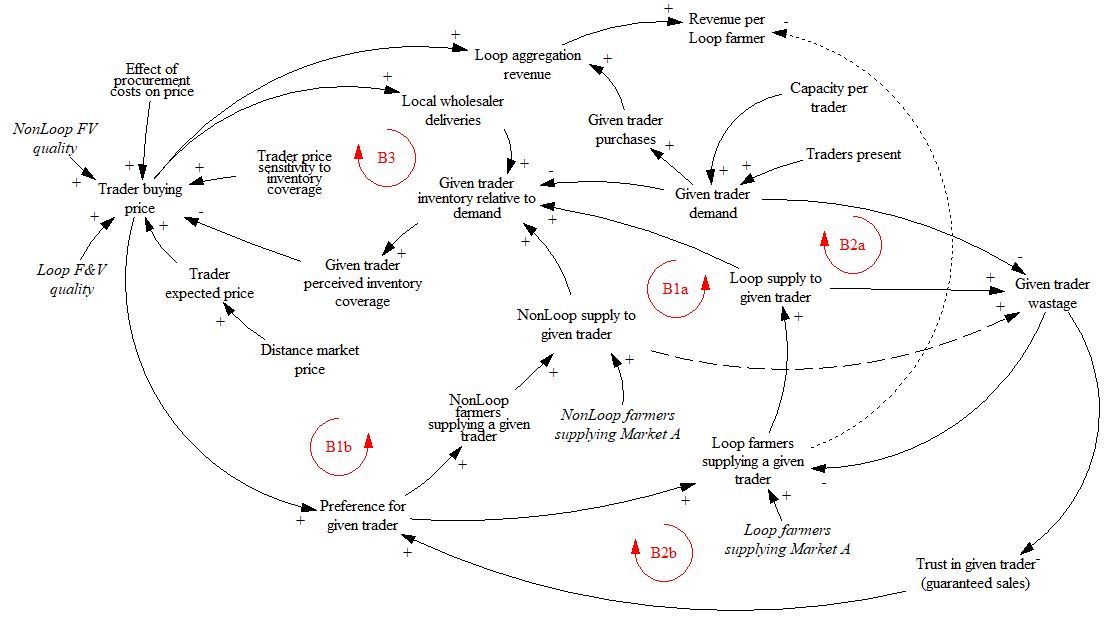 |
| --- |
| Figure S18: CLD of price dynamics. The ‘distance market price’ is only applicable to distance traders; see in text for other traders. The ‘local wholesaler deliveries’ is only relevant for local wholesalers in Markets A and B. Feedbacks: B1 – balancing feedbacks between the increasing supply of Loop (B1a) and non-Loop (B1b) produce to a given trader, which increases the trader’s inventory coverage and produces an adverse effect on the per unit price; B2 – short-term increases in supplies to a given trader without an associated increase in trader capacity can produce detrimental impacts on trader wastage rates and the trust in the trader to guarantee sales for farmers; B3 – increased wholesale prices in Market B can lead to the increased trade of wholesale produce from Market A to Market B, causing negative impacts upon prices in Market B (the equivalent applies for wholesale prices in Market A). |

population operate throughout the day; however, the time-series of local traders provided during the SGMB is divided by two, with half of the traders purchasing F&V in the morning and half of the traders operating in the afternoon (i.e. requiring one half-day outside of the market to travel). Lastly, retailers are assumed to sit in the market all day. In turn, the baseline numbers can be influenced by the entry/exit of traders from the market, based on their perceived revenues and costs (see ‘Trader profits and market entry’ below).

In line with the SGMB discussions around trader capacities, we keep the maximum quantity that can be procured per day constant (i.e. we assume the traders use the same vehicles over the course of the year, although the seasonal differences in supply might cause different procurement quantities). The individual capacities for the different trader types are as follows:

- Distance trader in Market A: 2000 kg/half-day
- Local wholesalers in Markets A and B: 400 kg/half-day
- Retailers in Markets A and B: dynamic capacity depending on respective consumer demands (see Module D)

The demand of each trader type for F&V on a given half-day therefore equals the number of traders multiplied by their individual capacities. The ratio of each trader’s demand to perceived inventory coverage (i.e. the F&V available to sell downstream) forms the major input to the formulation of price (see below).

Based upon observations during the value chain analysis and SGMB, local wholesalers in Market A may shift and sell produce in Market B, and vice versa for traders based out of Market B. The model assumes that local wholesalers in Market A will shift produce to Market B if the expected profits of selling produce in the smaller market are

greater than those in Market A, accounting for the costs of transportation (1 Rs/kg) and market commission. In turn, the volume shifted is equal to the spare wholesale capacity within the receiving market – equal to the demand of the local wholesalers in the receiving market minus their perceived inventory coverage (i.e. their inventory coverage averaged over the last week to smooth any spiky demand excesses or shortages). In contrast, distance wholesalers only trade produce to markets external to the model, whilst retailers only sell F&V to consumers visiting their market.

Trader price formation

Based on the Sterman (2000, Chapter 20) price setting model, the formation of price follows essentially the same structure for each of the different traders in the model (Figure S18).

Unlike traditional economic equilibrium models, system dynamics models work in disequilibrium, owing to the various delays and feedbacks that make up the model structure (Sterman, 2000). As a consequence, prices offered to farmers by each trader-type in the model are predominantly driven by the changes in trader inventory coverages relative to

downstream demands. An excess inventory coverage drives down prices, as to increase the inventory clearance rate; in contrast, a deficit inventory pushes up prices, as to attract additional supplies need to meet downstream demands. The five key components of price formation are detailed below:

- **Inventory coverage:** As detailed immediately above, each trader demands a certain quantity of F&V in order to satisfy downstream demands. If supplies increase relative to demands, the traders will notice that their inventory (i.e. stock of available F&V) is filling. As a consequence, traders will reduce their prices on offer, leading to reduced supplies towards the particular trader (and the eventual recovery of prices). Consistent with the rest of the model, the ‘inventory coverage perception time’ of each trader type is equal to 14 half-days.
- **Price sensitivity to supply and demand**: The sensitivity of prices to the supply-demand balance is a function of an elasticity-type variable. The dynamics are parameterised so that if demand outweighs supply (leading to an inventory coverage approaching zero), then the price offered to sellers will increase to attract greater volumes of supply. Within the current iteration of the model, the price sensitivity parameter for each trader equals the default value of Sterman (2000) (i.e. -0.3). Owing to a lack of reliable and contradictory evidence, the elasticities of each trader’s prices undergo sensitivity analysis in Appendix C.
- **Expected prices:** Traders also consider the price they expect to receive when selling the produce. For distance traders, this is the price they expect to receive at the distance market, which is based on a time-series for the price of F&V in the state capital Patna (see ‘External distance market’ above). In turn, given the size and influence of distance

traders relative to the other traders in the region, all other traders (in both markets) base their expected price on the distance traders’ price.

- **F&V procurement costs:** The model also factors in the trader procurement costs based on data collected during the value chain surveys. For distance traders and wholesale traders, the expected costs are the sum of half-day vehicle costs, market commissions

and other miscellaneous costs (e.g. tolls, bribes, cold storage rent during relevant scenarios). In turn, vehicle costs are omitted from the retailer’s costs in Market A and Market B. These costs are smoothed over the last 14 days and converted into per unit costs (i.e. Rs/kg) to remain consistent with the rest of the price module.

- **F&V quality**: The model also factors in F&V quality as detailed in Module B; essentially F&V supplies considered low-quality are priced 50% of high-quality produce.

From here, Loop and non-Loop revenues from each trader are generated by multiplying the volume of Loop and non-Loop produce purchased by the traders by their respective quality-adjusted buying prices. Farmers only receive 95% of the potential revenue however, as traders operate a 5% levy to offset any wastages between the point of purchase and sale. The proportions of each trader’s downstream flow which are Loop and non-Loop are assumed to be equal to the proportion of Loop and non-Loop supply in the previous timestep. In turn, farmer revenues are exported into Module E to calculate the net marketing revenues from different markets over time.

The key parameters in this module were both assessed for their qualitative reliability (Appendix A) and implications for model sensitivity (Appendix C).

Cointegration of market prices

Market cointegration occurs when changes in prices in at least two markets move together (Ghosh, 2000; Sexton et al., 1991). This process occurs over space, where traders purchasing from one market may deem it profitable to sell their produce in another market, once accounting for costs such as transport and market commissions. To further evaluate model confidence, we assess the extent to which the two market environments (urban ‘Market A’ and local ‘Market B’) are co-integrated in reality, before assessing the extent to which the systems model captures the level of cointegration observed in reality.

Critically, if markets are cointegrated, then the impact of intervening in one market may then be transmitted to another co-integrated market. In reality, market integration may be weakened by market imperfections, including trade barriers, imperfect knowledge and risk aversion (Sexton et al., 1991). To this end, based around efforts to assess market efficiency and liberalisation in India, cointegration tests have been widely applied to market price timeseries of staple crops (Ghosh, 2000; Jha et al., 2008; Rani et al., 2017), F&V produce (Basu, 2006; Mohapatra set al., 2018; Tingre and Bhopale, 2019) and animal-sourced products (Behura and Pradhan, 1998; Saran and Gangwar, 2008).

| 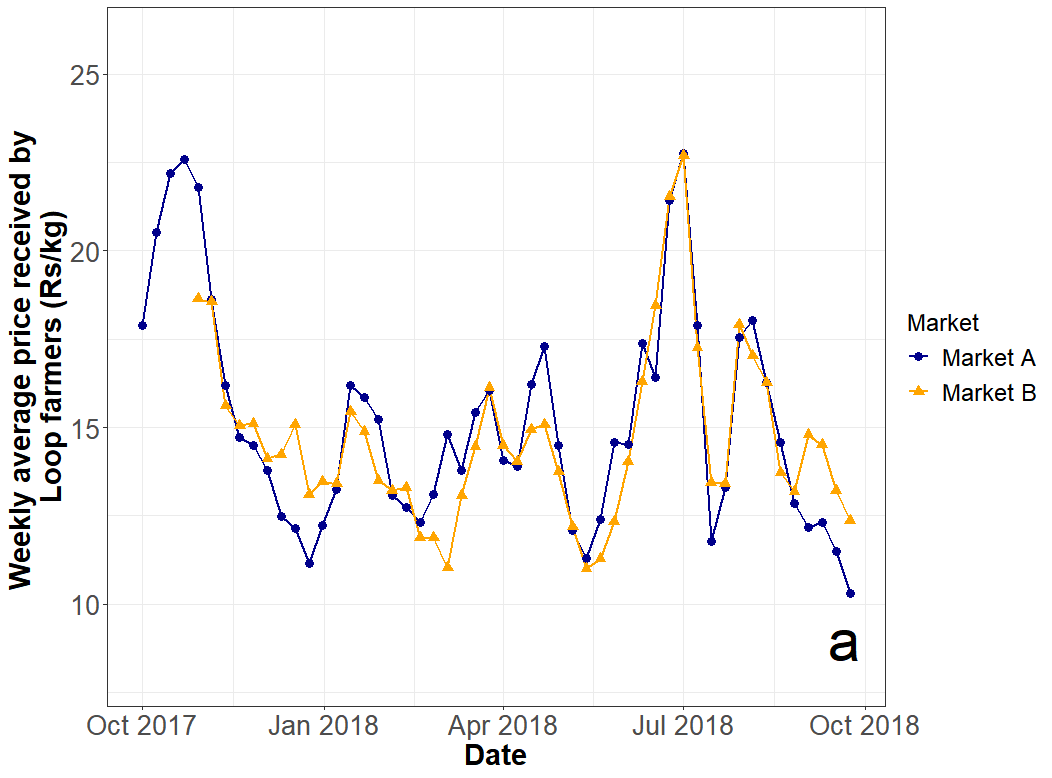 | 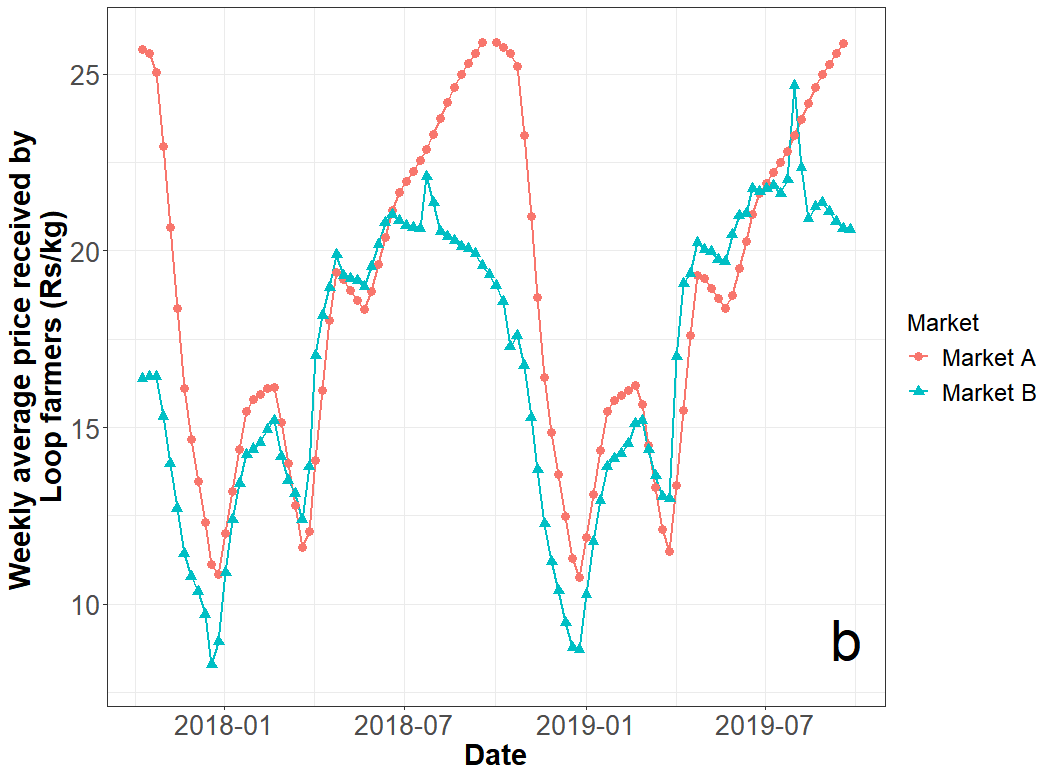 |
| --- | --- |
| Figure S19: (a) the *observed* weighted weekly F&V prices in ‘Market A’ and ‘Market B’; (b) the *modelled* weighted weekly F&V prices in ‘Market A’ and ‘Market B’. Note the different horizontal axes. | |

Market cointegration approaches generally constitute two stages. The first stage aims to confirm that the timeseries being co-integrated are non-stationary, meaning that the price value at any given time is at least partly dependent on time itself (e.g. through seasonality or a long-term trend). Commonly applied tests at this stage include the Augmented Dickey-Fuller (ADF) test (Dickey and Fuller, 1979) and the Phillips-Perron (PP) test (Phillips and Perron, 1988), which is a higher power non-parametric modification to the ADF test. Next, a cointegration test (e.g. Engle and Granger, 1987; Johansen and Juselius, 1990) is conducted to assess whether the time-series are integrated over the range of data available (i.e. the timeseries exhibit similar sensitivities to deviations). As per Basu (2006), who assessed the level of integration between wholesale potato prices in West Bengal, India, we conduct the Phillips-Perron test of non-stationarity and the Johansen and Juselius test of cointegration on our observed data and modelled timeseries from Bhojpur district,

Bihar. Importantly, owing to the lack of price data from non-Loop actors, these tests can only ascertain whether the prices received by Loop actors are co-integrated.

Deriving from Loop dashboard data, sales in the large twin markets of Kayamnagar and Arra are classified as ‘Market A’, whilst sales in the four local markets in the region are classified as ‘Market B’. In turn, we include data across the widest range of dates as possible, which corresponds to October 1^st^ 2017 to September 23^rd^ 2018 for ‘Market A’, and October 11^th^ 2017 to September 23^rd^ 2018 for ‘Market B’. To aggregate from individual timeseries to a combined timeseries of the 47 different crop types (as in our SDM), we calculate weighted averages for each market (i.e. ‘Market A’ or ‘Market B’) by summing the individual transaction prices, multiplied by the contributions of the individual transactions to the total Loop volume sold in the market in a given week (i.e. 1, 2… 52). The resulting timeseries are printed in Figure S19a. In turn, the modelled timeseries are the weighted average prices of Loop sales in both markets, aggregated to the weekly scale (Figure S19b). To avoid only assessing market integration over the parameterisation period (i.e. the period when the model was being set-up), we extend the modelled timeseries over an extra year (i.e. up until September 2019).

In order to meet the conditions for the Johansen and Juselius cointegration test, we need to confirm that the timeseries are non-stationary (Basu, 2006). Using the specialist timeseries cointegration and root unit package “urca” (Pfaff et al., 2016) in the statistical software R (R, 2013), we conduct the Phillips-Perron unit root test (Phillips and Perron, 1988) for each of the two observed and two modelled timeseries. If the unit-root null is rejected for the first difference of the timeseries, but not rejected for the level (i.e. the raw timeseries values), then we can infer that the series contains one unit root and is integrated of order one. As shown in Table S4, we are unable to reject the null hypotheses (i.e. that the series have a unit root) across all of the four timeseries; therefore, we are also unable to accept the alternate hypotheses that the series are stationary.

| Table S4: Outcomes of the Phillips-Perron unit root tests for the observed and modelled price timeseries in Market A and Market B. Significance codes: ** denotes significance at the p < 0.05 level. Critical values for different significance levels: -2.582 (10%), -2.890 (5%), -3.495 (1%) | | |
| --- | --- | --- |
| **Timeseries** | **Phillips-Perron (at level)** | **Phillips-Perron (at first difference)** |
| Observed – Market A | -2.366 | -5.111 ** |
| Observed – Market B | -2.904 | -4.840 ** |
| Modelled – Market A | -2.004 | -3.514 ** |
| Modelled – Market B | -1.627 | -6.013 ** |
|  | | |

From here, we run Johansen and Juselius (1990) cointegration tests to assess the presence of cointegrating vectors (‘r’) between the Market A and Market B price timeseries, in both the observed and modelled datasets. For the observed timeseries, we are able to reject the null hypothesis that the timeseries are not co-integrated (r = 0), with the trace statistic (26.01) exceeding the 95% critical value (Table S5). However, we are unable to reject the second null hypothesis that there are more than one cointegrating vectors (r > 1), with the trace statistic less than the 95% critical value of 8.18 (Table S5). Consistent with the results for the observed data, we are also able to reject the null hypothesis that there are no cointegrating vectors (r = 0) between the modelled price timeseries of Market A and Market B; likewise, we are unable to reject the second null hypothesis that the number of cointegrating vectors are more than one (Table S5). As a consequence, consistency between these cointegration tests helps to build confidence in the model’s ability to capture real-world price movements and relationships.

| Table S5: Outcomes from the Johansen and Juselius cointegration tests on the observed and modelled price timeseries in Market A and Market B. ** denotes significance at the p < 0.05 level. | | | | |
| --- | --- | --- | --- | --- |
| **Timeseries set** | **Null hypothesis** | **Alternate hypothesis** | **λ-trace value** | **95% critical value** |
| Observed | r = 0 | r > 0 | 26.01 ** | 17.95 |
|  | r ≤ 1 | r > 1 | 7.23 | 8.18 |
| Modelled | r = 0 | r > 0 | 29.90 ** | 17.95 |
|  | r ≤ 1 | r > 1 | 8.03 | 8.18 |
|  |  |  |  |  |

Unsold supplies

This model also factors in the possibility that traders are unwilling to purchase F&V (i.e. due to insufficient downstream demand), meaning farmers must sell to alternative traders within the same market. In Market A, any produce within the distance trader’s inventory coverage that remains unsold after four timesteps flows towards the local trader’s inventory coverage (rather than disappearing from the model/market all together). The same process also occurs from local traders to retailers. However, any unsold retail produce flows out of the retail stock with no destination. This dynamic reflects two key assumptions: (i) market retailers tend to be the least desirable destination for farmers, owing to their lower capacities and often relatively low prices; and (ii) F&V is a perishable item, and therefore cannot be recycled through the market infrastructure indefinitely.

The number of Loop and non-Loop farmers that are unable to sell their produce to their first-choice trader feed-back to the dynamics of ‘Loop trader preference’ (see above). In essence, a trader operating at near-capacity will be unable to guarantee the purchase of a farmer’s supply, leading to a weakening of the trust-based relationship and the increasing likelihood that farmers will supply a different trader in future.

Trader profits and market entry

The number of each type of trader at any point in time is a function of seasonality (see ‘Trader numbers and capacities’) and new entry/exits owing to trading profitability.

The revenue generated by each trader type is equal to the volume of F&V sold downstream (minus 5% wastage *en route*) multiplied by their selling price (i.e. the price the traders receive at the point of sale). For distance traders, the selling price is their expected price (i.e. in the large external market). The selling prices of wholesalers and retailers are internally generated by adding their per unit margin to the price offered to the farmers. Emerging from our SGMB discussions, the margin on any given day is linked to inventory coverage. Therefore, the margins of traders in the model fall linearly from 8 Rs/kg when their inventory coverage equals 0 (i.e. completely supply limited), to 0.5 Rs/kg when their inventory coverage equals 1 (i.e. capacity is saturated).

Various costs of trading detriment trader revenues. Within the baseline scenario, distance traders and local inter-market traders are subject to vehicle costs, market commissions, and sundry other costs (e.g. tolls and bribes). Similar to the on-farm costs of farmers (Module B), trader costs were collected from the value chain surveys and then converted into distribution functions of half-day costs, from which the model randomly samples from. Distance traders in market A face trading costs that average 800 Rs/half-day (excluding F&V purchases), whilst local wholesalers and retailers face trading costs of 200 Rs/half-day and 75 Rs/half-day respectively. In turn, wholesalers and retailers in Market B face trading costs that average 250 Rs/kg and 50 Rs/kg, respectively.

The entry of new traders into each market is based upon the ‘desired capacity’ model of Sterman (2000, p.807). The number of traders that desire to operate in the market is the product of the current number of traders (of each type) and the ‘effect of expected profits on desired capacity’. This latter factor is parameterised as a graphical function (Figure S15), relating the difference between cumulative trader revenues and costs to the desire of new traders to enter the market. Essentially, new traders enter the market once cumulative revenues outweigh cumulative costs and exit the market once cumulative costs outweigh cumulative revenues.

Market commissions

Market commission agents never purchase F&V supplies themselves, they take commission (akin to a finder’s fee) from both sellers and buyers once a transaction has been secured.

Here the model only includes gaddidars as commission generators, which eat into the returns earned by farmers and traders. Based on the SGMB discussions, gaddidars charge distance traders in Market A 8% of the transaction value, and the remaining local traders and farmers 5%.

E.5 Module D: Retail demand

Module D represents the model’s furthest point downstream in the F&V food system, calculating the volumes of F&V purchased by retail consumers in Market A and Market B. Based on our SGMB discussions, Markets A and B are the retail outlets for an initial 15,000 and 10,000 people, respectively. Reflecting the Bihar-wide rate of population growth from 2001-2011 (Census of India, 2011), the consumer populations grow by 2.3%/year. In turn, the average household size is 5.5 persons/household (as per our survey data for Bhojpur district), and one person from each household is assumed to purchase F&V for household consumption twice per week. As a consequence, the number of consumers demanding F&V on any given half-day equals:

$${Retail consumers}_{m,t}=\left( \frac{{Customer population}_{m,t}}{5.5} \right)\times\frac{1}{7}$$

Where *m* represents the market (i.e. A or B) and *t* represents the given half-day.

Based on the market commodity demand model of Sterman (2000, p.812), the volume of F&V demanded by each consumer is inversely related to the retail selling price, whereby a price increase leads to an increase in the demand elasticity and a contraction of consumer purchases (and vice versa). Moreover, retail purchases may be limited by the volume of available F&V within the retailers’ inventory (i.e. each consumer demands more than they can actually purchase). The three key parameters relating consumer demands to retailer prices are as follows:

- **Reference consumer demand:** This parameter refers to the baseline volume of F&V consumers wish to purchase on each half-day. In line with NSSO (2013), consumers dependent on the urban-based Market A are parameterised to consume 177 grams/capita/day, whilst consumers dependent on the more rural Market B are parameterised to consume 159 grams/capita/day (Rich and Dizyee, 2016). Therefore, the reference consumer demands are set to 3.4 kg and 3.1 kg for Market A and Market B, respectively, assuming that (i) retail consumers purchase F&V for their household twice per week (SGMB data), and (ii) households average 5.5 members (as per household survey data).
- **Reference retail price:** As per Sterman (2000)**,** the reference price is the price used to initialize the model given a linear demand curve, with the slope of the demand curve changing as prices deviate from the reference price. As prices rise above the reference price, the elasticity of demand increases (and vice versa). Therefore, the reference price acts as a threshold, beyond which prices are generally considered to be relatively unaffordable – triggering a decline in the per consumer demands from the reference consumer demand. Based on SGMB discussions, the reference price is set at 20 Rs/kg in both Market A and Market B.
- **Demand elasticity** relates the rate of change in consumer demands to the change in retail prices. Here the model uses expenditure elasticities for F&V in rural and urban areas of Bihar from secondary data from the Department of Horticulture, Government of Bihar (Kumari and Singh, 2016). Therefore, the value for ‘urban vegetables’ is used for Market A retailers (0.96), while the value for ‘rural vegetables’ is used for Market B retailers (0.88).

As mentioned above, a scenario may arise where consumer demands temporarily exceed the volume of F&V available to retailers (i.e. abrupt switch in farmer supplies to the alternative market). In such an event, all of the F&V available to the retailers will be sold and spread evenly amongst the consumers on that given half-day (i.e. the model assumes that all consumers in the same market have equal access, as opposed to a first-come first-serve approach), meaning that the actual volume of F&V purchased by consumers may be less than their baseline demand.

With farmers sourcing the vast majority of their F&V from the market, the current model does not distinguish between the market demands of farming households and non-farming households within the population. Therefore, an alternate version of the model may be set up by adjusting the retail demands for for the demands of farming households who have already consumed some of their own F&V production (see Appendix E.2). To assess the extent to which this alternate model may affect the results presented in Section 3 of the main manuscript, we assume that the 12,087 farming households are split between Markets A and B in a ratio of 3:2 (as per the ratio of the overall consumer households described above). In turn, the reference consumer demands are weighted by the number of farming households and the volume of F&V they consumed from their own production. The model also assumes that like the non-farming population, farming households visit the market twice per week to purchase their remaining F&V demand.

As an illustrative example of the alternate model, suppose that the baseline reference demand on any given half-day in Market B is 3.1 kg/half-day, and there are 3000 non-Loop farming households and 1200 Loop farming households, and 10,000 non-farming households. In turn, Loop households are consuming their own produce at 0.190 kg/household/half-day, and non-Loop households are consuming their own produce at 0.165 kg/household/half-day (Appendix E.2). Therefore, the weighted average retail demand for consumers in Market B purchasing twice per week would equal:

$$Adjusted Market B retail demand in alternate model=$$

$\left( \left( 3.1-7\times0.190 \right)\times\left( \frac{1200}{14,200} \right) \right)+\left( \left( 3.1-7\times0.165 \right)\times\left( \frac{3000}{14,200} \right) \right)+\left( 3.1\times\left( \frac{10,000}{14,200} \right) \right)=$

$2.74$ (kg/consumer/half-day)

Applying this set-up to both Markets A and B, we re-run the 12 future scenarios explored in the main manuscript (Figures 6 and 7). Overall, the mean absolute percentage error (MAPE) of the outcome ratios in the alternate model versus the original model is 0.652%. Furthermore, running a two-tailed student’s t-test finds no significant difference between the means of the two sets of outcome ratios (t = -0.121, df = 141.9, p = 0.904). Therefore, we are confident that the alternate model set-up has a minimal impact upon the dynamics and implications of the different scenarios explored.

E.6 Module E: Farmer revenues & costs

The final module collects all of the individual revenues and costs generated by Loop and non-Loop farmers in order to (a) feedback into the various decision-making processes and preferences driving F&V flows downstream, and (b) assess the impacts of different scenarios on the livelihood outcomes of farmers. A selection of the key outcomes and costs calculated within Module E are listed below (apply for both Loop and non-Loop farmers):

- Loop and non-Loop revenues from Market A and Market B (weighted by trader)
- Loop and non-Loop daily per unit (i.e. Rs/kg) revenues from Market A and Market B (weighted by trader)
- Average transport and marketing costs per Loop and non-Loop farmer in Market A and Market B (weighted by trader)
- Percentage of marketable supplies by each farmer population to Markets A and B that were successfully sold
- Per unit revenue of Loop pathways relative to non-Loop pathways (weighted by market)

Investments in land expansion

Farmers are able to invest their profits back into their F&V production activities. Reflecting discussions in the SGMB sessions, the model attempts to capture the investment of F&V profits into (a) more land for cultivation, and (b) improved inputs (e.g. seeds and pesticides) for enhanced F&V yields.

The seasonal allocation of agricultural land for horticultural production is described in Module B; however, the model also makes it possible for farmers to invest financially to increase the area of land that can be cultivated. The key driver is the cumulative profit made by Loop farmers (and non-Loop farmers for non-Loop land areas). Further conditions and assumptions need to be met before farmers invest:

- It is assumed that the minimum unit of purchasable land is 0.1 katha (~0.003 hectares), which according to our household survey data, costs an average of Rs 7600 in Bhojpur district;
- The willingness of farmers to invest in land is proportional to their land investment rate, which is a decimal between 0 and 1 representing the proportion of their cumulative profits they are willing to invest. Both Loop and non-Loop values are set at 0.2.
- Farmers may only invest in land at the start of each season if their seasonal profits from horticulture are growing (i.e. their cumulative profit at the end of the most recent season is higher than at the season before).

Therefore, Loop farmers with Rs 80,000 in cumulative profits will be willing to spend 20% on land purchases. The Rs 16,000 available to purchase land corresponds to an additional land area of 0.21 katha (0.1 [katha] x 16000/7600). As the model does not differentiate between individual farmers, the above scenario would mean every Loop farmer expands their land by the same area. Therefore, if the above example happens with 2000 Loop farmers, then the area of land under F&V cultivation will expand by 421 katha.

Investment in higher yielding inputs

The structure of this investment is essentially the same as the investment in land, whereby the following conditions must be met to trigger investment:

- The willingness of farmers to invest in higher yielding inputs is proportional to their yield investment rate. Similarly, both Loop and non-Loop values are set at 0.2.
- Farmers may only invest in inputs at the start of each season if their seasonal profits from horticulture are growing.

From here, a graphical function converts the investment rate into a yield boost, whereby a 20,000 Rs investment produces a 1% boost in yield above the base seasonal rates. This investment-yield conversion is currently based on the modeller’s intuition and is therefore subject to sensitivity analysis to understand the implications of the variable and its uncertainties on model dynamics (Appendix C). The yield changes are exported to their respective land and yield submodules in Module B.

Trust in the aggregation scheme

As introduced in Module A, Loop adoption is a function of the trust in the aggregation scheme to produce farmer-facing benefits over non-aggregation pathways. Essentially, this metric is self-reflective, whereby Loop farmers compare their per unit marketing returns (Rs/kg) and guaranteed sales under Loop on any given half-day with the equivalent figures from one month ago. If the farmers find that aggregation is underperforming relative to the previous month, then the stock of trust in Loop will decline, producing weaker Loop adoption and stronger Loop dis-adoption (i.e. farmers switching back to being non-Loop farmers).

Utility of the aggregation scheme

In reality, Loop and non-Loop farmers are able to discuss the benefits of aggregation within their social groups. Therefore, the second feedback on aggregation adoption and dis-adoption is a direct comparison of the performance of Loop (guaranteed sales and per unit marketing returns) relative to non-Loop. The structure for this part of the model was inspired by Grabowski et al. (2019), where they used system dynamics modelling to explore the utility of technology adoption in pigeonpea cultivation systems in Malawi. The following equation calculates the utility of Loop returns relative to non-Loop supply pathways at time *t*:

$${Loop profit utility}_{t}=\left( \frac{{Loop per unit profits}_{t}}{{LOOP per unit profits}_{t}+{nonLOOP per unit profits}_{t}} \right)-0.5$$

The logic here is that if Loop returns exceed non-Loop returns, then utility > 0. Alternatively, if non-Loop returns exceed Loop returns, then return utility < 0, having a negative effect on Loop utility relative to non-Loop. Loop utility from guaranteed sales uses the same structure, just with returns replaced by the percentage of Loop supply on any given day that was successfully sold to the preferred trader at the market (i.e. ‘guaranteed sales’). Finally, the Loop utility stock is driven by a biflow that sums and smooths the two utilities over the last month.

Transport costs and service charges recovered

Farmers participating in the aggregation scheme on any given day must cover the costs of (a) market transportation, (b) the aggregator’s commission, and (c) a service charge recovered by the NGO Digital Green used to fund and sustain operations.

Informed by Loop dashboard data and SGMB discussions, the aggregation transport cost to Market A is parameterised as 1300 Rs, and the equivalent figure for Market B is 1800 Rs (the markets are both assumed to be 10 km away from the farming population). In turn, the average aggregation capacity is 2000 kg, meaning average transportation costs equal 0.65 Rs/kg and 0.90 Rs/kg for Market A and Market B, respectively. Aggregators then receive an additional 0.1 Rs/kg (i.e. 10 ‘paisa’), before an extra 10% service charge is recovered.

Therefore, as an illustration, total aggregation cost to Market A:

$\left( \left( \frac{1300}{2000} \right)+0.1 \right)\times1.1=0.825 Rs/kg$, of which 0.075 Rs/kg is recovered as the service charge

Total aggregation cost to Market B:

$\left( \left( \frac{1800}{2000} \right)+0.1 \right)\times1.1=1.1 Rs/kg$, of which 0.1 Rs/kg is recovered as the service charge

In comparison, non-Loop transport costs are streamlined using figures from our SGMB discussions, varying randomly between 1.00-1.50 Rs/kg on any given half-day.

| **Table S6: Model variables informed by graphical function timeseries. See Table S1 for the data sources.** | | |
| --- | --- | --- |
| **Variable** | **Graphical function coordinates (x = time, y = variable output)** | **Module** |
| Seasonal farmer market supply frequency (days) | GRAPH(TIME) (1, 10.0), (152, 10.0), (394, 18.0), (610, 18.0), (882, 10.0), (1124, 18.0), (1340, 18.0), (1612, 10.0), (1854, 18.0), (2070, 18.0), (2342, 10.0), (2584, 18.0), (2800, 18.0), (3072, 10.0), (3314, 18.0), (3530, 18.0), (3802, 10.0), (4044, 18.0), (4260, 18.0), (4532, 10.0), (4774, 18.0), (4990, 18.0), (5262, 10.0), (5504, 18.0), (5720, 18.0), (5992, 10.0), (6234, 18.0), (6450, 18.0), (6722, 10.0), (6964, 18.0), (7180, 18.0), (7452, 10.0), (7694, 18.0), (7910, 18.0), (8182, 10.0) | A |
| Seasonal F&V yields  (kg/katha/half-day) | GRAPH(TIME) (1, 0.22), (152, 0.22), (394, 0.13), (610, 0.14), (882, 0.22), (1124, 0.13), (1340, 0.14), (1612, 0.22), (1854, 0.13), (2070, 0.14), (2342, 0.22), (2584, 0.13), (2800, 0.14), (3072, 0.22), (3314, 0.13), (3530, 0.14), (3802, 0.22), (4044, 0.13), (4260, 0.14), (4532, 0.22), (4774, 0.13), (4990, 0.14), (5262, 0.22), (5504, 0.13), (5720, 0.14), (5992, 0.22), (6234, 0.13), (6450, 0.14), (6722, 0.22), (6964, 0.13), (7180, 0.14), (7452, 0.22), (7694, 0.13), (7910, 0.14), (8182, 0.22) | B |
| Price of staples | GRAPH(TIME) (1, 16.67), (63, 16.63), (123, 16.40), (185, 16.13), (247, 16.33), (303, 16.07), (365, 15.93), (425, 15.73), (487, 15.63), (547, 15.70), (609, 16.93), (671, 16.77)… (7976, 16.77)  [Note: The first 12 timesteps are repeated over the following ten years] | B |
| External market F&V price | GRAPH(TIME) (1, 40.0), (63, 39.0), (123, 24.0), (185, 16.0), (247, 24.0), (303, 25.0), (365, 17.0), (425, 30.0), (487, 28.0), (547, 33.0), (609, 35.0), (671, 38.0)… (7976, 38.0)  [Note: The first 12 timesteps are repeated over the following ten years] | C |
| Distance wholesalers in Market A | GRAPH(TIME) (1, 60), (184, 70), (364, 30), (730, 50), (914, 70), (1094, 30), (1460, 50), (1644, 70), (1824, 30), (2190, 50), (2374, 70), (2554, 30), (2920, 50), (3104, 70), (3284, 30), (3650, 50), (3834, 70), (4014, 30), (4380, 50), (4564, 70), (4744, 30), (5110, 50), (5294, 70), (5474, 30), (5840, 50), (6024, 70), (6204, 30), (6570, 50), (6754, 70), (6934, 30), (7300, 50), (7484, 70), (7664, 30), (8030, 50) | C |
| Local wholesalers in Market A | GRAPH(TIME) (1, 80), (184, 120), (364, 60), (730, 80), (914, 120), (1094, 60), (1460, 80), (1644, 120), (1824, 60), (2190, 80), (2374, 120), (2554, 60), (2920, 80), (3104, 120), (3284, 60), (3650, 80), (3834, 120), (4014, 60), (4380, 80), (4564, 120), (4744, 60), (5110, 80), (5294, 120), (5474, 60), (5840, 80), (6024, 120), (6204, 60), (6570, 80), (6754, 120), (6934, 60), (7300, 80), (7484, 120), (7664, 60), (8030, 80) | C |
| Retailers in Market A | GRAPH(TIME) (1, 100), (184, 110), (364, 80), (730, 100), (914, 110), (1094, 80), (1460, 100), (1644, 110), (1824, 80), (2190, 100), (2374, 110), (2554, 80), (2920, 100), (3104, 110), (3284, 80), (3650, 100), (3834, 110), (4014, 80), (4380, 100), (4564, 110), (4744, 80), (5110, 100), (5294, 110), (5474, 80), (5840, 100), (6024, 110), (6204, 80), (6570, 100), (6754, 110), (6934, 80), (7300, 100), (7484, 110), (7664, 80), (8030, 100) | C |
| Local wholesalers in Market B | (1, 3), (184, 5), (364, 3), (730, 3), (914, 5), (1094, 3), (1460, 3), (1644, 5), (1824, 3), (2190, 3), (2374, 5), (2554, 3), (2920, 3), (3104, 5), (3284, 3), (3650, 3), (3834, 5), (4014, 3), (4380, 3), (4564, 5), (4744, 3), (5110, 3), (5294, 5), (5474, 3), (5840, 3), (6024, 5), (6204, 3), (6570, 3), (6754, 5), (6934, 3), (7300, 3), (7484, 5), (7664, 3), (8030, 3) | C |

Appendix references

Bass, F.M., 1969. A New Product Growth for Model Consumer Durables. Management Science 15, 215–227. https://doi.org/10.1287/mnsc.15.5.215

Basu, J., 2006. Cointegration and Market Integration: An Application to the Potato Markets in Rural West Bengal, India, in: International Association of Agricultural Economists (IAAE) - 2006 Annual Meeting, August 12-18, 2006, Queensland, Australia. p. 16.

Behura, D., Pradhan, D., 1998. Cointegration and Market Integration: An Application to the Marine Fish Markets in Orissa. Indian Journal of Agricultural Economics 53, 344–350.

Census of India, 2011. District Census Handbook: Bhojpur.

Chapman, A., Darby, S., 2016. Evaluating sustainable adaptation strategies for vulnerable mega-deltas using system dynamics modelling: Rice agriculture in the Mekong Delta’s An Giang Province, Vietnam. Science of The Total Environment 559, 326–338. https://doi.org/https://doi.org/10.1016/j.scitotenv.2016.02.162

Cooper, G.S., Rich, K.M., Shankar, B., Rana, V., Ratna, N.N., Kadiyala, S., Alam, M.J., Nadagouda, S.B., 2021. Identifying ‘win-win-win’ futures from inequitable value chain trade-offs: A system dynamics approach. Agricultural Systems 190, 103096. https://doi.org/https://doi.org/10.1016/j.agsy.2021.103096

Dickey, D.A., Fuller, W.A., 1979. Distribution of the Estimators for Autoregressive Time Series With a Unit Root. Journal of the American Statistical Association 74, 427–431. https://doi.org/10.2307/2286348

Digital Green, 2017. Digital Green’s LOOP: Pooling Technology and Extension Networks for Market Access. New Delhi, India.

Engle, R.F., Granger, C.W.J., 1987. Co-Integration and Error Correction: Representation, Estimation, and Testing. Econometrica 55, 251–276. https://doi.org/10.2307/1913236

Fafchamps, M., 1992. Cash Crop Production, Food Price Volatility, and Rural Market Integration in the Third World. American Journal of Agricultural Economics 74, 90–99. https://doi.org/10.2307/1242993

FAO, 2016. Handbook on Agricultural Cost of Production Statistics. Rome, Italy.

Ghosh, M., 2000. Cointegration Tests and Spatial Integration of Rice Markets in Indi. Indian Journal of Agricultural Economics 55, 616–626.

Grabowski, P., Schmitt Olabisi, L., Adebiyi, J., Waldman, K., Richardson, R., Rusinamhodzi, L., Snapp, S., 2019. Assessing adoption potential in a risky environment: The case of perennial pigeonpea. Agricultural Systems 171, 89–99. https://doi.org/https://doi.org/10.1016/j.agsy.2019.01.001

Jha, R., Murthy, K.V.B., Sharma, A., 2008. Market Integration in Wholesale Rice Markets in India, in: Jha, R. (Ed.), The Indian Economy Sixty Years After Independence. Palgrave Macmillan UK, London, pp. 233–246. https://doi.org/10.1057/9780230228337_13

Johansen, S., Juselius, K., 1990. Maximum likelihood estimation and inference on cointegration - with applications to the demand for money. Oxford Bulletin of Economics and Statistics 52, 169–210. https://doi.org/https://doi.org/10.1111/j.1468-0084.1990.mp52002003.x

Kumar, P., Kumar, A., Parappurathu, S., S S Raju, D., 2011. Estimation of Demand Elasticity for Food Commodities in India. Agricultural Economics Research Review 24.

Kumari, M., Singh, R.G., 2016. Demand supply and trade prospects of major fruits and vegetables in Bihar. International Journal of Agricultural Science and Research 6, 269–278.

Maestre, M., Poole, N., Henson, S., 2017. Assessing food value chain pathways, linkages and impacts for better nutrition of vulnerable groups. Food Policy 68, 31–39. https://doi.org/https://doi.org/10.1016/j.foodpol.2016.12.007

Mohapatra, S., Singh, J., Kumar, S., 2018. Cointegration among Major Cauliflower Markets in Punjab. Indian Journal of Economics and Development 14, 330. https://doi.org/10.5958/2322-0430.2018.00138.5

Neuwirth, C., Hofer, B., Schaumberger, A., 2016. Object view in spatial system dynamics: a grassland farming example. Journal of Spatial Science 61, 367–388. https://doi.org/10.1080/14498596.2015.1132641

NHB, 2018. ‘MIS Weekly Report’ dataset [WWW Document]. National Horticultural Board, Ministry of Agriculture & Farmer’s Welfare (GoI). URL http://www.nhb.gov.in/OnlineClient/Weekly.aspx (accessed 4.29.19).

NHB, 2015. Horticulture Crops Estimates for the Year 2013-14 and 2014-15 [WWW Document]. National Horticultural Board, Ministry of Agriculture & Farmer’s Welfare (GoI).

NIFTEM, 2013. Area, Production and Productivity of Fruits and Vegetables in Different States of the Country.

NSSO, 2013. Household Consumer Expenditure, NSS 68th Round Sch1.0 Type 1: July 2011—June 2012. New Delhi, India.

Pfaff, B., Zivot, E., Stigler, M., 2016. Package “urca” [WWW Document]. CRAN Repository. URL https://cran.r-project.org/web/packages/urca/urca.pdf (accessed 5.15.21).

Phillips, P.C.B., Perron, P., 1988. Testing for a Unit Root in Time Series Regression. Biometrika 75, 335–346. https://doi.org/10.2307/2336182

R, 2013. R: A language and environment for statistical computing. Vienna, Austria.

Rani, R., Singh, R., Tewari, H., Singh, S.K., Singh, P.K., 2017. Integration of major Indian maize markets : a cointegration analysis. International Journal of Agricultural and Statistical Sciences 13, 601–606.

Reardon, T., Echeverria, R., Berdegué, J., Minten, B., Liverpool-Tasie, S., Tschirley, D., Zilberman, D., 2019. Rapid transformation of food systems in developing regions: Highlighting the role of agricultural research & innovations. Agricultural Systems 172, 47–59. https://doi.org/https://doi.org/10.1016/j.agsy.2018.01.022

Rich, K.M., Dizyee, K., 2016. Policy options for sustainability and resilience in potato value chains in Bihar: a system dynamics approach, NUPI Working Paper.

Saran, S., Gangwar, L.S., 2008. Analysis of Spatial Cointegration amongst Major Wholesale Egg Markets in India. Agricultural Economics Research Review 21.

Sexton, R.J., Kling, C.L., Carman, H.F., 1991. Market Integration, Efficiency of Arbitrage, and Imperfect Competition: Methodology and Application to U.S. Celery. American Journal of Agricultural Economics 73, 568–580. https://doi.org/10.2307/1242810

Sterman, J.D., 2000. Business Dynamics: Systems Thinking and Modeling for a Complex World. Irwin/McGraw-Hill, New York City, USA.

Tingre, A., Bhopale, A., 2019. Price Stability and Co-integration Analysis of Tomato for Major Markets of Maharashtra. International Journal of Pure & Applied Bioscience 7, 510–514. https://doi.org/http://dx.doi.org/10.18782/2320-7051.7467

USDA, 2019. India: Grain and Feed Annual Report. Washington DC, USA.

1. Household survey reference period: Rabi 2017-2018 (October – February), Zaid 2018 (March – May), and Kharif 2018 (June – September). [↑](#footnote-ref-1)
2. Of the 47 different F&V types Loop supplied to markets in Bhojpur district between October 2017 and September 2018, the top ten by proportion were: (i) Bottle gourd (22.2%); (ii) Cauliflower (13.3%); (iii) Tomato (9.23%); (iv) Brinjal (9.07%); (v) Lady finger (7.88%); (vi) Sponge gourd (5.43%); (vii) Long bean (5.22%); (viii) Pointed gourd (2.38%); (ix) Mango (2.21%); (x) Chilli (2.10%). [↑](#footnote-ref-2)
3. As state-wise brinjal production and yield were not available from NHB (2015), the equivalent statistics for the growing year 2012-2013 were obtained from NIFTEM (2013). [↑](#footnote-ref-3)
4. In Bihar, one katha is equal to approximately 1/80 hectacre. [↑](#footnote-ref-4)
5. The NHB dataset is available: <http://www.nhb.gov.in/OnlineClient/MISDailyReport.aspx> [↑](#footnote-ref-5)
